# Immediate and Delayed Response of Simulated Human Atrial Myocytes to Clinically-Relevant Hypokalemia

**DOI:** 10.3389/fphys.2021.651162

**Published:** 2021-05-26

**Authors:** Michael Clerx, Gary R. Mirams, Albert J. Rogers, Sanjiv M. Narayan, Wayne R. Giles

**Affiliations:** ^1^Centre for Mathematical Medicine and Biology, School of Mathematical Sciences, University of Nottingham, Nottingham, United Kingdom; ^2^Department of Medicine and Cardiovascular Institute, Stanford University, Stanford, CA, United States; ^3^Department of Physiology and Pharmacology, University of Calgary, Calgary, AB, Canada

**Keywords:** hypokalemia, mathematical modeling, inwardly rectifying K^+^ current, sodium potassium (Na^+^/K^+^-ATPase) pump, action potential repolarization, atrial fibrillation (AF), renal dialysis, plasma potassium levels

## Abstract

Although plasma electrolyte levels are quickly and precisely regulated in the mammalian cardiovascular system, even small transient changes in K^+^, Na^+^, Ca^2+^, and/or Mg^2+^ can significantly alter physiological responses in the heart, blood vessels, and intrinsic (intracardiac) autonomic nervous system. We have used mathematical models of the human atrial action potential (AP) to explore the electrophysiological mechanisms that underlie changes in resting potential (V_r_) and the AP following decreases in plasma K^+^, [K^+^]_o_, that were selected to mimic clinical hypokalemia. Such changes may be associated with arrhythmias and are commonly encountered in patients (i) in therapy for hypertension and heart failure; (ii) undergoing renal dialysis; (iii) with any disease with acid-base imbalance; or (iv) post-operatively. Our study emphasizes clinically-relevant hypokalemic conditions, corresponding to [K^+^]_o_ reductions of approximately 1.5 mM from the normal value of 4 to 4.5 mM. We show how the resulting electrophysiological responses in human atrial myocytes progress within two distinct time frames:

(i) Immediately after [K^+^]_o_ is reduced, the K^+^-sensing mechanism of the background inward rectifier current (I_K1_) responds. Specifically, its highly non-linear current-voltage relationship changes significantly as judged by the voltage dependence of its region of outward current. This rapidly alters, and sometimes even depolarizes, V_r_ and can also markedly prolong the final repolarization phase of the AP, thus modulating excitability and refractoriness.

(ii) A second much slower electrophysiological response (developing 5–10 minutes after [K^+^]_o_ is reduced) results from alterations in the intracellular electrolyte balance. A progressive shift in intracellular [Na^+^]_i_ causes a change in the outward electrogenic current generated by the Na^+^/K^+^ pump, thereby modifying V_r_ and AP repolarization and changing the human atrial electrophysiological substrate.

In this study, these two effects were investigated quantitatively, using seven published models of the human atrial AP. This highlighted the important role of I_K1_ rectification when analyzing both the mechanisms by which [K^+^]_o_ regulates V_r_ and how the AP waveform may contribute to “trigger” mechanisms within the proarrhythmic substrate. Our simulations complement and extend previous studies aimed at understanding key factors by which decreases in [K^+^]_o_ can produce effects that are known to promote atrial arrhythmias in human hearts.

## Introduction

Detailed knowledge of plasma electrolyte (e.g., K^+^, Na^+^, Ca^2+^, and Mg^2+^) levels, and an understanding of the consequences of even small changes in one or more of them, are needed for optimal clinical management of various chronic diseases (Franse et al., 2000; Ahmed et al., 2007; Barrett Bowling et al., 2010; El-Sherif and Turitto, 2011; Alderman et al., 2012; Wang et al., 2013; Collins et al., 2017; Krogager et al., 2020). Examples of the translational implications of changed electrolyte levels can be found in renal physiology/pathophysiology and dialysis (Genovesi et al., 2008; Krueger et al., 2011; Zimmerman et al., 2012; Vincenti et al., 2014), in case management of hypertension (Wrong, 1961; Krogager et al., 2020), and in clinical cardiac arrhythmia management (Weaver and Burchell, 1960; Zaza, 2009; Goyal et al., 2012). During in-hospital acute patient care, plasma levels of Mg^2+^, Ca^2+^, K^+^, and Na^+^ are monitored closely, and plasma K^+^ levels ([K^+^]_o_) can provide essential information for diagnoses and guidance for treatment (Macdonald and Struthers, 2004; Mehta and Emmett, 2017).

Regulatory mechanisms for plasma electrolytes are both robust and precise; and even small changes in divalent cation levels or in [K^+^]_o_ can have significant physiological and clinical consequences. Indeed, large-scale studies have linked such electrolyte changes to fluid imbalances and resulting congestion, reduced muscle cramping, and lowered incidence of cardiac arrhythmias (Barrett Bowling et al., 2010; El-Sherif and Turitto, 2011; Collins et al., 2017) including atrial fibrillation (AF; Krijthe et al., 2012; Buiten et al., 2014). As a result, close monitoring of electrolyte levels is recommended. Hypokalemia is known to occur in 20 to 30% of hospitalized patients (Paice et al., 1986; Collins et al., 2017), but whether or not prevention or correction of abnormal plasma potassium levels has a beneficial impact on morbidity and mortality has yet to be shown in a large-scale trial (Mehta and Emmett, 2017). A computational review focusing on plasma electrolyte changes, and in particular on hemodialysis effects, was published by Passini et al. (2014).

In this study we focus on one potentially important plasma electrolyte alteration; the electrophysiological consequences of small, but significant, decreases in plasma K^+^ levels (*hypokalemia*) in the human atrium. It is well established that small reductions (1–2 mM) in [K^+^]_o_ can occur during renal dialysis (Zimmerman et al., 2012; Vincenti et al., 2014), treatment for hypertension (Krogager et al., 2020), diuretic therapies (Osadchii, 2010), or after intense or chronic exercise (Paterson, 1997). Our goal is to gain a detailed understanding of the short- and long-term effects of moderate hypokalemia on human atrial electrophysiological parameters including the resting potential (V_r_), action potential (AP) waveform, refractory period dynamics and conduction velocity (CV). Previously, we have studied some of the effects of *hyper*kalemia on human atrial electrophysiological properties (Nygren and Giles, 2000) and reviewed hypokalemia in the ventricle (Trenor et al., 2018).

Before discussing our methods and results, we briefly review the normal atrial [K^+^]_o_ level, the critical [K^+^]_o_-sensing mechanisms in atrial myocytes, and some important past experimental results on hyper- and hypo-kalemic effects on atrial electrophysiology.

### Normal or Physiological Levels of [K^+^]_o_

In most *in vitro* physiological experimental settings, [K^+^]_o_ is set by the superfusate at 5.4 mM. In contrast, a recent detailed electrolyte analysis of plasma from healthy adult human establishes the mean of this important electrolyte parameter to be around 4.2 mM (Collins et al., 2017). Analyzing both a control group (*n* = 339,297) and a large and geographically varied cohort of patients (*n* = 911,689) with heart failure, chronic kidney disease, and/or diabetes mellitus this study found mortality was lowest in the group with potassium levels from 4 to 5 mM (Collins et al., 2017; Mehta and Emmett, 2017). This was in agreement with an earlier study (Macdonald and Struthers, 2004) that reported lowest mortality for [K^+^]_o_ levels of 4.5 to 5.5 mM. Indeed, the paper by Collins et al. defines “normal” levels as 4–4.5 mM, with “moderate hypokalemia” and “moderate hyperkalemia” given as 3.5–4 mM and 4.5–5 mM, respectively. Based on this information, it appears that many experimental studies of cardiovascular physiology (and pathophysiology) have been carried out under somewhat hyperkalemic conditions (5.4 mM instead of 4–4.5 mM). One reason for this is that much more stable electrophysiological recordings can be obtained in 5.4 than in 4 mM [K^+^]_o_.

### [K^+^]_o_-Sensing Mechanisms in Human Atrial Myocytes

#### Inwardly Rectifying K^+^ Channels

This distinction between 5.4 and 4 mM [K^+^]_o_ is important, since all skeletal muscles and most cardiac tissues exhibit a mechanism for sensing and then rapidly transducing changes in [K^+^]_o_ into electrophysiological responses (altered V_r_ or AP waveform). This mechanism is finely tuned to [K^+^]_o_ levels between 3 and 8 mM (Bailly et al., 1998; Bouchard et al., 2004; Burns et al., 2004; Trenor et al., 2018). These very quickly developing [K^+^]_o_-dependent changes arise mainly from altered current flow through a subset of the inwardly rectifying K^+^ channels, Kir2.1, 2.2, and 2.3 (Melnyk et al., 2002; Anumonwo and Lopatin, 2010) that exhibit a highly non-linear current-voltage relationship (Dhamoon and Jalife, 2005; Hibino et al., 2010). The corresponding transmembrane K^+^ current, denoted I_K1_ in cardiac myocytes, can strongly regulate the resting potential and alter the final phase of repolarization of the AP (Shimoni et al., 1992; Anumonwo and Lopatin, 2010; Weiss et al., 2017).

#### Na^+^/K^+^ Pump-Mediated Regulation of Electrophysiology and Contraction

However, it is also well known that the changes in [K^+^]_o_ that are frequently encountered in clinical settings can significantly alter Na^+^/K^+^ pump current (Glitsch, 2001; Burns et al., 2004). K^+^ binding at the Na^+^/K^+^ external (plasma) site has a dissociation constant, K_d_, (or half-maximal concentration, K_0.5_) of approximately 1.5 to 2 mM in both mammalian ventricular tissue (Nakao and Gadsby, 1989; Glitsch, 2001) and in human atrial myocytes (Workman et al., 2003), with the response saturating at approximately 8 mM. Thus, even relatively small changes in [K^+^]_o_ can significantly alter the electrogenic current I_NaK_ (Blanco and Mercer, 1998). In cardiac myocytes, in most physiological settings, this outwardly directed current can hyperpolarize V_r_ by approximately 5 mV (Workman et al., 2003) and altered Na^+^/K^+^ pump activity may also produce changes in the AP waveform or duration (Glitsch, 2001; Workman et al., 2003; Sanchez et al., 2012). In addition, and perhaps more importantly, maintained and relatively long-term changes in Na^+^/K^+^ pump activity can significantly alter intracellular Na^+^ concentration, [Na^+^]_i_ (Glitsch, 2001).

### Electrophysiology of Human Atrial Myocytes

The availability of human atrial tissue (usually excised segments of the right atrial appendage) from open heart surgical procedures has enabled detailed electrophysiological studies of these preparations in both healthy and diseased conditions (Gelband et al., 1972; Ten Eick and Singer, 1979). A number of well-known papers provide important background on human atrium responses to both hypo- and hyper-kalemia for our study. Significant findings include:

i The resting potential in atrial myocytes is largely determined by I_K1_, a current which is highly sensitive to changes in [K^+^]_o_. I_K1_ is sensitive to [K^+^]_o_ via three distinct mechanisms: a conductance decrease with decreased [K^+^]_o_ (Sakmann and Trube, 1984); a shift in the reversal potential; and a change in the voltage-dependence of rectification (see Lu, 2004 for a review).
ii The resting potential is also modulated by the atrial Na^+^/K^+^ pump current (Rasmussen et al., 1984, 1986; Workman et al., 2003), which is highly sensitive to [K^+^]_o_ and [Na^+^]_i_.
iii Although hyperkalemic increases above 5 mM are known to strongly depolarize V_r_, very few studies in human atrium have investigated this effect at [K^+^]_o_ levels in the hypokalemic range. A tissue study by Gelband et al. (1972) shows a sharp change in the [K^+^]_o_ dependence at this point, while Ten Eick and Singer (1979) even show depolarization at very low [K^+^]_o_ (see Supplementary Figure 1).
iv The resting potential, V_r_, at the [K^+^]_o_ level that is considered “normal” in *in vitro* atrial experimental settings (5.4 mM) is somewhat more depolarized than in ventricular myocytes, with values in the –80 to –75 mV range commonly reported (Ten Eick and Singer, 1979; Mary-Rabine et al., 1983; Workman et al., 2001).
v Even moderate changes in V_r_ affect sodium current availability, thereby significantly altering excitability and CV (Whalley et al., 1994; Skibsbye et al., 2016), so that [K^+^]_o_ changes may alter the effectiveness of drugs that target the fast sodium current (Singh and Vaughan Williams, 1971).
vi Electrophysiological properties of the human atrial myocyte change or remodel significantly under long lasting or chronic AF conditions. Two prominent features of the new phenotype are a marked reduction in atrial AP duration and a significant hyperpolarization of V_r_ (Workman et al., 2001).

These findings inspired three questions, which we address using mathematical models of the atrial AP: (i) What is the role of the non-linear (inwardly rectifying) background K^+^ current I_K1_ in regulating the resting potential and AP in hypokalemia? (ii) How do the two outward currents in human atrium that are strongly modulated by [K^+^]_o_ (I_K1_ and I_NaK_) interact in settings that mimic clinical hypokalemia? (iii) What are the main functional changes in human atrial tissue strands that result from short- or long-term hypokalemia?

To address these questions and relate our findings to previous publications that have studied similar problems (Passini et al., 2014; Vincenti et al., 2014), the first part of our mathematical modeling study focuses on the almost immediate changes in the AP, one second after a sudden change in [K^+^]_o_. This initial part is expected to reveal the immediate effects due to changes in I_K1_. Part two consists of a similar analysis done under quasi steady-state conditions (after 15 min), when changes due to the Na^+^/K^+^ pump have developed. In part three, these insights regarding [K^+^]_o_-induced effects, are applied to assess excitability and conduction dynamics in a simulated strand of human atrial myocytes. Previous work by Passini et al. (2014) has shown that replicating electrolyte changes is challenging for human atrial myocyte models. We attempted to mitigate this by performing a multi-model study. Although we restrict ourselves to a single model in the main manuscript, we will refer to supplementary results from the other models throughout.

## Materials and Methods

Simulations were performed using models of single human atrial myocytes and human atrial tissue strands. In total, our study used seven published mathematical models of the human AP, as listed in Table 1. All models and scripts that were used are available for download at: https://github.com/CardiacModelling/AtrialLowK.

**TABLE 1:** Summary of the mathematical models of the human atrial action potential and resting potential that form the basis of this study.

**Model**	**Ancestors**	**Stabilizes**	**V_r_ at 1 Hz**	**Three [K^+^]_o_ effects on I_K1_**	**AP at 2.5 mM [K^+^]_o_**	**[K^+^]_i_ can vary in time**
Nygren et al., 1998	Lindblad et al., 1996 (rabbit atrial)	Yes*	−73 mV	Yes	No	Yes
Maleckar et al., 2008	Nygren et al., 1998	Yes	−79 mV	Yes	Yes	Yes
Koivumäki et al., 2011	Nygren et al., 1998	No	−77 mV	Yes	No	Yes
Courtemanche et al., 1998	Luo and Rudy, 1994 (guinea pig ventricular)	Yes*	−82 mV	No	Initially	Yes
Ni et al., 2017	Courtemanche et al., 1998; Colman et al., 2013	Yes	−77 mV	No	No	No
Grandi et al., 2011	Grandi et al., 2010 (human ventricular), Maleckar et al., 2008 (human atrial), Shannon et al., 2004 (rabbit ventricular)	Yes	−74 mV	Yes	No	No
Voigt et al., 2013	Grandi et al., 2011	Yes	−75 mV	Yes	Initially	No

*Denotes models that stabilize after minor modifications (as described in the text).

### Mathematical Models of the Human Atrial Action Potential

A number of different mathematical models of the human atrial AP and resting potential have been published (for reviews see Wilhelms et al., 2013; Heijman et al., 2016; Vagos et al., 2018). While some of these models include detailed subcellular spatial modeling of intracellular Ca^2+^ homeostasis/buffering, we have used less complex models that have a limited number of “compartments” representing average concentration in the cytoplasm, sarcoplasmic reticulum, and dyadic space, etc.

As the first step in this study, the seven published models that are listed in Table 1 were implemented. CellML 1.0 (Hedley et al., 2001) implementations of the models by Courtemanche et al. (1998); Nygren et al. (1998), and Maleckar et al. (2008) were downloaded from the Physiome Model Repository (Yu et al., 2011) and then converted to Myokit “mmt” format (Clerx et al., 2016). Novel Myokit and CellML implementations were created for the Grandi et al. (2011); Koivumäki et al. (2011), and Ni et al. (2017) models based on code kindly provided by the authors. These three “re-implementations” were verified by comparing the calculated state derivatives of the original implementations with our versions at the default initial conditions. A Myokit implementation of the Voigt et al. (2013) model was provided by the original authors. Myokit and CellML files for all models used in this study are provided in the online repository accompanying paper.

All figures were made using an approach similar to that described by Cooper et al. (2011), where essential variables in each model were annotated with labels and unit information so that simulations could be written in a model-agnostic manner. In some models a number of different currents (e.g., I_CaL_) are coded in such a way that the total current is divided into components according to their ion selectivity or subcellular localization. When plotting these currents, we show the sum of all components. The Nygren et al. and Maleckar et al. models both include a “restricted diffusion” or “cleft” space immediately outside the cell membrane, in which the [K^+^]_o_ profile can differ from the bulk [K^+^]_o_. In simulations with these models, any effects of restricted diffusion within these cleft spaces were removed by assigning the same [K^+^]_o_ values for both the bulk extracellular (or superfusate) volume and the cleft space.

### Single-Cell Simulation Procedures

Single human atrial myocyte AP simulations were performed using Myokit’s CVODE-based (Hindmarsh et al., 2005) simulation engine. All simulations were run with a tolerance setting of 10^–8^ for both absolute and relative errors. All single myocyte models were paced at 1 Hz using a 0.5 ms stimulus. The first stimulus was applied 50 ms after the start of the simulated experiment, the second stimulus at 1,050 ms, and so on.

Before running experiments, the models were conditioned by: (i) setting the stimulus amplitude to twice threshold value required to elicit an AP (determined at baseline [K^+^]_o_); and (ii) pacing each model until the AP was stable from beat to beat. Specifically, each model was paced until the maximum relative change in any state variable was less than 0.001% from beat to beat: max*_i_*|*x*_*i*_[*j*]−*x*_*i*_[*j*−1]|/*r*_*i*_ < 10^−5^. where *x_i_[j]* is the value of the *i*-th state variable at *t* = *j*⋅1,000*ms*, and *r*_i_ is the range (maximum – minimum) of the *i*-th state variable during the first AP. For most models this “stable” condition was reached within a few hundred paces, although >10,000 paces were needed for the Grandi-Pandit-Voigt et al. model. Our implementation of the Koivumäki et al. model never stabilized enough to meet this criterion, so was instead used with a state obtained after > 10,000 paces.

Minor modifications had to be made to the models of Courtemanche et al. and Nygren et al. to ensure stabilization. In both models, the equations for the internal K^+^ concentration [K^+^]_i_ were updated to include the influence of the stimulus current, which has become a standard approach to ensure conservation of charge (Jacquemet, 2007). The Nygren et al. (1998) model contains a constant term Φ_Na,en_ introduced to approximately balance the charge influx corresponding to the stimulus. This extra term is not required when stimulus charges are explicitly accounted for in [K^+^]_i_, and so Φ_Na,en_ was set to zero (Jacquemet, 2007).

### Na^+^/K^+^ Pump I–V Relationships

Some figures show simulated APs along with I–V curves for I_NaK_. These I–V curves were calculated by varying membrane potential but holding other variables affecting I_NaK_ (e.g., the internal sodium concentration) fixed at their values just before the graph was made (i.e., corresponding to the left-most point of the shown AP).

### Simulations of Human Atrial AP Conduction in Simulated Strands

To investigate the effects of low [K^+^]_o_ conditions on AP refractoriness and CV, simulations were carried out based on 1–D strands consisting of electrotonically coupled atrial myocytes. This approach was implemented to approximate the experimental procedure used by Smeets et al. (1986). Simulations were run using a forward-Euler integration scheme implemented in Myokit (Clerx et al., 2016), using a step size of 0.001 ms and a Rush-Larsen approximation for Hodgkin-Huxley gate variables. At each selected time, *t*, the intercellular current flow between any two neighboring atrial myocytes (denoted *i* and *j*) was calculated as *I*_ij_(*t*) = *g*(*V*_i_–*V*_j_) and used to evaluate the state variable derivatives in the model for each cell (with conductance *g* determined as explained below). In this study, 200 myocytes were included, leading to a strand length of 2 cm (assuming a myocyte length of 0.1 mm). Excitation and AP propagation were initiated at 1 Hz by delivering 2 ms stimuli, adjusted to be 2 times the diastolic threshold. These stimuli were applied only to the left-most 5 myocytes of each simulated strand.

To determine the cell-to-cell conductance *g*, all 200 atrial myocyte models were set to the same initial conditions that were used in single myocyte simulations (i.e., the steady-state conditions for a single cell at 1 Hz). Simulations were run from this starting point, and *g* in each strand was varied until a CV of 80 cm/s was obtained (Spach et al., 2007). CV was calculated by estimating the start time of the AP in cells 50 through to 150, and using linear least squares (with the myocyte dimensions given above).

After the stimulus parameters and cell-to-cell conductance were estimated using the procedure above, each simulated strand was preconditioned by: (i) using a [K^+^]_o_ of 5.4 mM, applying the single-cell steady-state to each of the connected cell models; and (ii) simulating 50 APs at a frequency of 1 Hz. This approach was adopted to mimic commonly-used experimental conditions at a heart rate of a healthy subject at rest. To check whether this procedure led to a sufficiently stable system, we then simulated one further AP and calculated the relative change in all state variables before and after this beat. This was found to be in the range of 0.01 to 0.2% in all models (see Supplementary Section 6).

### Short- and Long-Term Effects of Hypokalemia on the Refractory Period and Conduction Velocity

In both the single myocyte and the tissue strand components of this study, the immediate and longer-term effects of step changes to different levels of [K^+^]_o_ were evaluated by analyzing changes in AP waveform, V_r_, and non-linear I–V characteristics at a time point either 1 s or 15 min after the [K^+^]_o_ change.

In the human atrial strand simulations, a standard S_1_/S_2_ paired stimulus protocol was used to determine the functional refractory period (FRP). By convention, the FRP was defined as the shortest time between the two successive stimuli that successfully elicited a second propagating AP with a CV of at least 40 cm/s. Values for the wavelength (WL) of conduction or “wavelength of the cardiac impulse” parameter (Smeets et al., 1986) were obtained as the product of FRP and CV.

## Results

### Immediate Effects

Although several computational models of the human atrial AP and resting potential have been published, none of them have been formulated or validated using data obtained at low (or perhaps even normal) [K^+^]_o_, and predictions for low plasma electrolyte levels from published models are known to vary qualitatively and quantitatively (Passini et al., 2014). We therefore started our study by evaluating seven different published mathematical models of human atrial electrophysiology. A feature-by-feature comparison of these models is given in Table 1, and baseline APs and calcium transients are shown in Supplementary Figure 2. A comparison of the currents active during the AP and in the diastolic phase is shown in Supplementary Figure 3 while Supplementary Section 2.3 compares the models’ restitution characteristics. A detailed description of the I_K1_ and I_NaK_ equations in each model is provided in Supplementary Section 3.

We inspected each model:

i to check whether each exhibited stable resting potential values and characteristic AP waveforms at a 1 Hz heart rate;
ii to ensure a sufficiently strong Na^+^ current to sustain propagated AP responses in our atrial strand simulations; and
iii perhaps most importantly, to determine whether each model could accurately reproduce the three-part dependence of I_K1_ on [K^+^]_o_, via its reversal potential, maximum conductance, and most importantly its rectification.

From the models that fit these criteria, we selected the one by Voigt et al. (2013) for use in the main text – but we will refer to the Supplementary Material throughout for complementary results in the other models.

**FIGURE 1:**
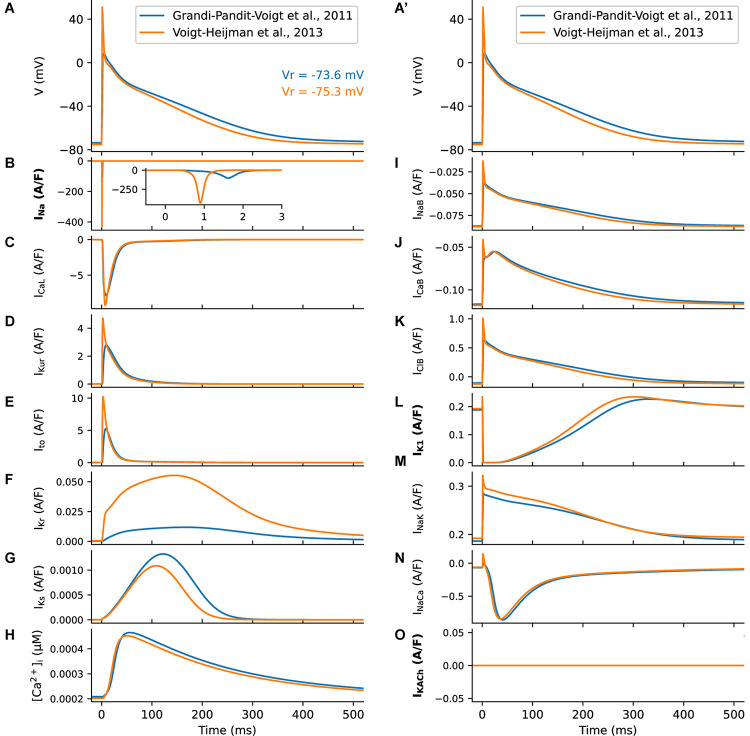
Summary of the human atrial membrane action potential and underlying transmembrane ionic currents. The AP traces **(A, A’)** and underlying currents were computed using the original Grandi et al. (2011) model (blue) and the Voigt et al. (2013) modification. Where the underlying model formulation differs, labels for the respective transmembrane currents have been highlighted in bold. **(B)** Shows marked differences in the Na^+^ currents that generate the AP upstroke, caused by the changed Na^+^ current formulation. **(C)** shows L-type Ca^2+^ currents; **(D)** illustrates the rapidly activating delayed rectifier K^+^ current, I_Kur_; and **(E)** denotes the Ca^2+^-independent transient outward current, I_to_. **(F, G)** show the fast and slow time- and voltage-dependent delayed rectifier K^+^ currents, IK_r_ **(F)** and I_Ks_ **(G)**. **(H)**, illustrates model generated changes in intracellular [Ca^2+^]_i_ that take place during the AP. In both models there are three time-independent or background currents: the Na^+^ current is shown in **(I)**; the background Ca^2+^ current in **(J)** and the background Cl^–^ current in **(K)**. Data depicting the inwardly rectifying K^+^ current, I_K1_ is shown in **(L)**. Although the equations for this current differ between models, only modest differences between the produced current traces are observed. The electrogenic current generated by the Na^+^/K^+^ pump is shown in **(M)**. A second distinct electrogenic current produced by the Na^+^/Ca^2+^ exchanger is illustrated in **(N)**. In the Voigt et al. (2013) model it is also possible to simulate one of the effects of acetylcholine by utilizing a second distinct background K^+^ current, I_KACh_. In this study, we did not make use of this feature and thus this current was turned off as illustrated in **(O)**. Note that the two superimposed APs in **(A, A’)** are identical; this was done to more clearly illustrate the sizes and time courses of the underlying currents in each column relative to the AP waveform.

In Figure 1, we compare the Voigt-Heijman et al. model’s output with that of its predecessor, the Grandi-Pandit-Voigt et al. model. The three currents that were modified in the Voigt-Heijman et al. model are highlighted in bold on the y-axis labels. Both simulations were run at baseline conditions and paced at a 1 Hz frequency. The AP and V_r_ data in Figure 1A confirm that the resulting two AP waveforms are very similar. The values of the resting potential, V_r_, both fall within the range that has been established in human atrial tissue (Gelband et al., 1972; Mary-Rabine et al., 1983; Workman et al., 2001).

Compared to its predecessor, the Voigt-Heijman et al. model produces a much larger transient Na^+^ current, as shown in Figure 1B. This difference is important: It is essential if this model is to be used in our simulations of AP propagation (summarized in Figure 5). A similar modification was used in previous studies using the Grandi-Pandit-Voigt model in a tissue context (Deo et al., 2013; Martins et al., 2014).

The Voigt-Heijman et al. model also generates significantly larger K^+^ currents than those in the Grandi-Pandit-Voigt et al. model (Figures 1D–G), despite the equations for these currents being identical in both models. Thus, the Voigt et al. (2013) model may have a larger safety factor for repolarization, or repolarization reserve (Varró and Baczkó, 2011). As seen in Figure 1M the Na^+^/K^+^ currents are very similar in these two models of the human atrial AP.

One drawback of this model, and of most other human atrial AP models, is that intracellular K^+^ ([K^+^]_i_) is a *fixed parameter.* In principle, this choice could limit the model’s ability to accurately reproduce long-term effects of hypokalemia due to slow intracellular concentration changes. However, none of the simulated experiments in this study would be expected to significantly alter [K^+^]_i_. This is confirmed in Supplementary Figures 15–17, showing at most 2 mM deviation in [K^+^]_i_ for the three models with time-varying [K^+^]_i_ and [K^+^]_o_-sensitive I_K1_ rectification.

An essential property of the biophysical behavior of a strong inward rectifier such as I_K1_ is the effect of [K^+^]_o–_-sensitivity on its conductance and rectification properties, which counteract the effect of hypokalemia on its electrochemical driving force. The magnitude and direction of these three effects is plotted independently in Supplementary Figures 7, 9, while Supplementary Figure 8 compares the [K^+^]_o_-sensitivity of I_K1_ rectification in the used models.

**FIGURE 2:**
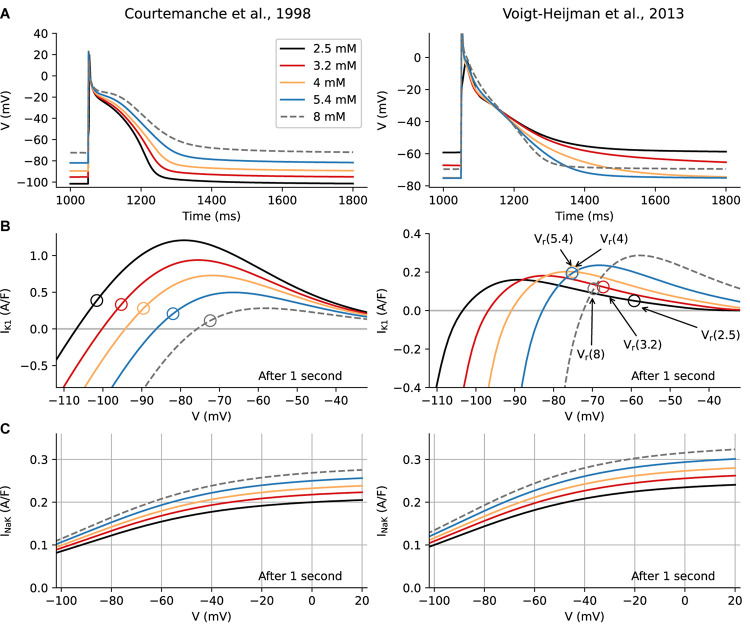
Immediate effects of alterations in [K^+^]_o_ levels on human atrial Action Potential (AP), Resting Potential (V_r_), and underlying ionic currents. Computational results for the Courtemanche et al. model are shown in the left column, and analogous results from the Voigt-Heijman et al. model are shown on the right. Each panel in **Row A** shows five superimposed AP waveforms that illustrate how [K^+^]_o_-induced changes in repolarization affect the AP waveform and the resting potential, V_r_, 1 s after a step change in [K^+^]_o_. Note that in both models, hyperkalemia depolarizes V_r_, but *hypo*kalemic effects are model-dependent. In the Courtemanche et al. model the waveforms for the [K^+^]_o_ levels representing clinical hypokalemia (3.2 and 2.5 mM) show a pronounced hyperpolarization and a shortening of the AP, while in the Voigt-Heijman et al. model hypokalemia depolarizes V_r_ and prolongs the AP. The origins of this difference can be seen in **Row B**, which depicts the I-V curve for I_K1_ at the selected [K^+^]_o_ levels. Note that these are color-coded to match the AP traces in Row A, while open circles denote the resting potential of the myocyte in the corresponding [K^+^]_o_ condition. In the Courtemanche et al. model, the only effect of lowering [K^+^]_o_ is an increase in electrochemical driving force for I_K1_ (which is more larger at more depolarized potentials, causing the appearance of a peak current that moves “left and up”). In the Voigt-Heijman et al. this effect is still present, but the [K^+^]_o_ -sensitivity of I_K1_ rectification and conductance counteracts this effect. Importantly, as described in section “Results” and Supplementary Section 3, the [K^+^]_o_ dependence of I_K1_ gives rise to a complex pattern of changes in the non-linear inwardly rectifying I–V relation for I_K1_. The superimposed I–V curves in each panel of **Row C** illustrate the corresponding I–V curve of electrogenic current, I_NaK_, generated by the Na^+^/K^+^ pump, in each of the [K^+^]_o_ levels that were used for this analysis.

Figure 2 shows a side-by-side comparison of predicted electrophysiological changes after 1 s of exposure to an altered [K^+^]_o_ in the Courtemanche et al. and Voigt-Heijman et al. models. In Figure 2A, the respective baseline AP waveforms and V_r_ levels, and the responses of both to selected changes in [K^+^]_o_ in the range 2.5 to 8 mM, are shown. Note that in the Voigt-Heijman et al. model at [K^+^]_o_ concentrations of less than approximately 4 mM, V_r_ moves in the *depolarizing* direction and the AP *lengthens*. In contrast, in the Courtemanche et al. model the same changes in [K^+^]_o_ produce a progressive *hyperpolarization* of V_r_ and *shortening* of the AP. Information concerning the ionic basis for these changes in V_r_ and the atrial AP waveforms is presented in Figure 2B, where the I–V relationships for the background inwardly rectifying K^+^ current, I_K1_, are shown. It is apparent that the qualitative behavior is very different for the two models, although I_K1_ is significantly affected by [K^+^]_o_ in both. Finally, the I–V curves in Figure 2C show that there are immediate effects on I_NaK_ (via V_r_), but that these are moderate compared to the dramatic changes in I_K1_.

The significantly different pattern of results shown in the two columns of Figures 2A,B should be considered in conjunction with well-known molecular features and biophysical properties of the K^+^ channels that generate I_K1_ in human atrium (see section “Introduction”). In response to decreases in [K^+^]_o_ the outward component of the non-linear I–V curves for I_K1_ not only translates in the hyperpolarizing direction (as would be expected from the increased electrochemical driving force for K^+^) but also “flattens.” While the hyperpolarizing translation leads to an increased current at V_r_ (as seen in the Courtemanche model), the flattening effect is dominant, so that a smaller outward current is generated in low [K^+^]_o_ (Shimoni et al., 1992; Trenor et al., 2018). The superimposed I-V curves in Figure 2B also reveal that, when the effects on rectification and maximum conductance are taken into account, changes in [K^+^]_o_ result in the outward component of the I–V curves exhibiting “cross over” within a range of membrane potentials (–80 to –60 mV). Importantly, this voltage range falls within the normal resting potential and the mid-to-final repolarization phases of the human atrial AP (Bouchard et al., 2004; Dhamoon and Jalife, 2005; Trenor et al., 2018). It is mainly for this reason that in 4 to 2.5 mM [K^+^]_o_, although the electrochemical driving force for K^+^ fluxes has *increased*, the size of the outward current component *decreases* substantially. Thus, very soon after lowering [K^+^]_o_, this very rapid change in I_K1_ is the main reason for a depolarization of V_r_, and significantly lengthens the final phase of repolarization in the ventricle (Shimoni et al., 1992). In marked contrast, when this essential feature of I_K1_ is not incorporated into the model, as in the Courtemanche et al. model, V_r_ progressively hyperpolarizes and the AP shortens in low [K^+^]_o_.

In Supplementary Figure 10, we show the full V_r_-[K^+^]_o_ relationship predicted by the seven models, and compare it with the observations by Gelband et al. (1972) and Ten Eick and Singer (1979). This analysis shows that all models with [K^+^]_o_-sensitive I_K1_ predict a depolarization of V_r_ at severely hypokalemic levels, although the point where the depolarization sets in varies from 4 to 5 mM in the Voigt-Heijman, Grandi-Pandit-Voigt, and Nygren et al. models, to the much lower value of 2 mM in the model by Koivumäki et al. The 2014 paper by Passini et al., recommended using a modified version of the Courtemanche et al. model, in which V_r_ starts depolarizing at around 3 mM [K^+^]_o_.

### Delayed Effects

The computational results presented in Figure 2 revealed rapid changes in the background K^+^ current, I_K1_, in response to decreases in [K^+^]_o_. As noted in the Introduction, we analyze the range of [K^+^]_o_ known to be present in clinical situations, e.g., as a consequence of diuretic treatment for hypertension or heart failure as well as during renal dialysis procedures (Zaza, 2009; Zimmerman et al., 2012). To begin to gain an improved understanding of the ionic basis for atrial arrhythmias associated with hypokalemia, and to relate our findings to previous detailed studies of low [K^+^]_o_ conditions in experimental and clinical settings (Aronsen et al., 2015; Weiss et al., 2017), it was necessary to do corresponding AP simulations at times 10–15 min after altering [K^+^]_o_. This is because electrophysiological changes due to the Na^+^/K^+^ pump would be expected to develop very slowly compared to the ion channel-mediated mechanisms associated with I_K1_. Accordingly, in Parts II and III of our study, electrophysiological changes in both single myocyte and strand models were assessed and compared almost immediately (1 s) after changing [K^+^]_o_ levels, and also 15 min later (as an approximation for quasi steady-state conditions). Our rationale was that, in principle, comparison of these two time points could reveal rapidly-developing consequences of hypokalemia (which might serve as a localized “trigger” for atrial rhythm disturbances) and more slowly-developing and long-lasting alterations to the atrial substrate (that may contribute to proarrhythmia; Zhang et al., 2005; Krogh-Madsen et al., 2012; Pandit and Jalife, 2013).

**FIGURE 3:**
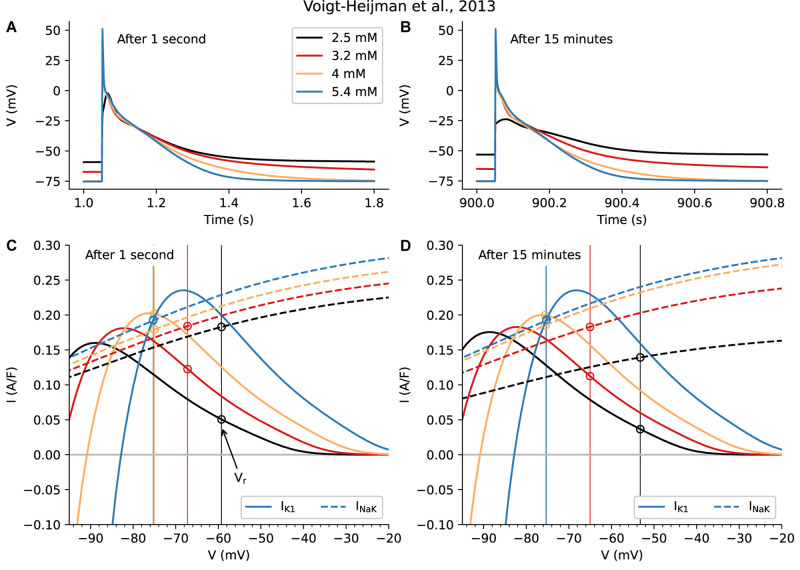
Evaluation of the Short- and Long-Term Physiological Effects of Changes in [K^+^]_o_ on the Background K^+^ Current, I_K1_, and the Na^+^/K^+^ Pump Current, I_P._ Data in the left column **(A, C)** was obtained 1 s after changing from 5.4 mM to the shown levels of [K^+^]_o_; the results in the right column **(B, D)** are 15 min after changes in [K^+^]_o_. The superimposed AP traces in **(A)** are similar to those in Figure 2 and are included here to provide a direct comparison of very rapid short-term changes in [K^+^]_o_ with those that develop in a time-dependent fashion, shown in **(C)**. Note that the overall patterns of changes in V_r_ and AP waveforms are similar when captured at 1 s vs. at 15 min. However, at 15 min in hypokalemic conditions (2.5 and 3.2 mM [K^+^]_o_) V_r_ depolarizes slightly more and the final phase of AP repolarization is slowed markedly. Note also the very small depolarization observed for 2.5 mM after 15 min. **(C, D)** provide a direct comparison of the size and voltage-dependence of I_K1_ and I_NaK_. Side-by-side comparison of these two components reveals that, as expected, there is a time-dependent decrease in I_NaK_ in the two hypokalemic [K^+^]_o_ conditions (3.2 and 2.5 mM). This, and the decrease in the outward current due to I_K1_, are primary reasons that V_r_ *depolarizes* to a new stable level. By contrast, the initial [K^+^]_o_ induced reduction in I_NaK_ seen when switching from 5.4 to 4 mM is almost fully restored after 15 min.

Figure 3 is based on these two sets of calculations, each done using the Voigt-Heijman et al., model, paced at 1 Hz for either 1 s or for 15 min after a sudden [K^+^]_o_ change, as described in Methods. Figure 3A (1 s) and Figure 3B (15 min) show the resulting changes in AP waveforms and in V_r_ at the selected values of [K^+^]_o_. Figures 3C,D show ionic currents due to I_K1_ (continuous lines) and the Na^+^/K^+^ pump current, I_NaK_ (broken lines).

As shown in Figure 3C, when [K^+^]_o_ is decreased, net outward I_NaK_ exhibits an immediate downward shift, confirming that its affinity for [K^+^]_o_ is a significant regulatory variable for Na^+^/K^+^ pump current in human atrium (Workman et al., 2003). The K_D_ for [K^+^]_o_ in the Voigt et al. (2013) model is set at 1.5 mM in accordance with classical experimental analyses (Glitsch, 2001).

The more slowly-developing, time-dependent changes shown in Figure 3D reveal that, for a small change from 5.4 to 4 mM, the initial downward shift (decrease) of I_NaK_ is partially recovered. This recovery occurs as a result of the well-known sensitivity of the Na^+^/K^+^ pump to changes in [Na^+^]_i_ (Glitsch, 2001). For larger decreases in [K^+^]_o_, to 3.2 or 2.5 mM, the Na^+^/K^+^ pump appears not to recover, but instead I_NaK_ is further decreased.

The superimposed data sets in Figures 3C,D can be used to address the fundamental question: What are the differences in the sizes of the low [K^+^]_o_-induced changes in I_K1_ vs. those due to the electrogenic Na^+^/K^+^ pump current, I_NaK_? Beginning with the current changes that occur in the range of the resting membrane potential (−80 to −50 mV), one can see from Figure 3C that the very rapid (within 1 s) changes in I_K1_ are at least as large as those due to the changes in I_NaK_. Thus, very soon after [K^+^]_o_ is decreased, I_K1_ is an important regulator of membrane potential and action potential duration (APD). In combination, the changes are likely to contribute to the “triggering mechanism” for atrial flutter/fibrillation. However, after 15 min the changes in I_NaK_ become more significant.

Second, and equally importantly, in the range of membrane potential that corresponds to the mid- to final repolarization phases of the AP (approx. −30 to −65 mV), the *immediate* current changes due to I_K1_ are larger than those due to I_NaK_. This reveals that the highly non-linear change in I_K1_ is the predominant factor in regulating atrial AP duration in the short term. However, after 15 min the change in I_NaK_ has also become a significant regulatory factor. It is important to recall that both AP duration and the time course of repolarization strongly regulate Ca^2+^ fluxes through L-type Ca^2+^ channels in cardiac preparations having approximately triangular shaped APs (Clark et al., 1996; Sah et al., 2003) such as the human atrial myocardium. In addition, even small changes in V_r_ during the diastolic interval can markedly alter electrogenic current generated by the Na^+^/Ca^+^ exchanger, and thus strongly modulate [Ca^2+^]_i_ (Clark et al., 1996).

Complementary results in the other models are shown in Supplementary Figures 11–17. For the models with [K^+^]_o_-sensitive I_K1_ rectification, they show a similar pattern of results, where the effects of small decreases in [K^+^]_o_ on I_NaK_ are compensated, while larger changes are exacerbated over time.

**FIGURE 4:**
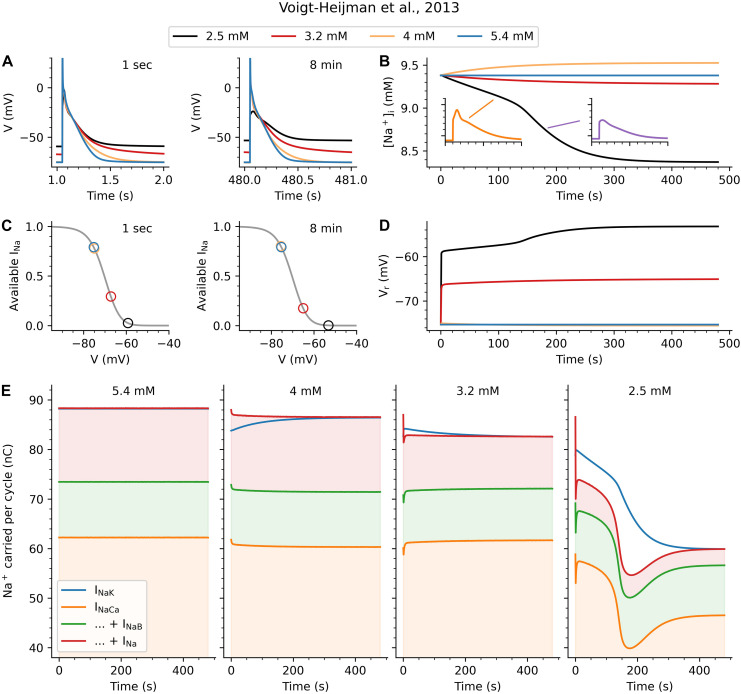
Mechanism underlying longer-term effects of [K^+^]_o_ on V_r_ and AP. **(A)** shows the AP either 1 s (left) or 8 min (right) after a change in [K^+^]_o_ from its baseline value of 5.4 mM. The same pattern of effects as in Figure 3 is observed here at 8 min, indicating that the system has stabilized. **(B)** shows how the [K^+^]_o_ changes can cause either a [Na^+^]_i_ increase (4 mM) or decrease (3.2 and 2.5 mM) over time. While the changes in [Na^+^]_i_ start gradually in all cases, the 2.5 mM hypokalemic condition reveals a secondary effect occurring after around 140 beats. During this rapid transition phase, [Na^+^]_i_ steeply declines and the AP waveform undergoes a qualitative change, from a triangular waveform to something resembling a subthreshold response. **(C)** is based on steady-state inactivation characteristics (gray lines) for I_Na_. It shows the amount of recovered (available) I_Na_ at V_r_ after 1 s or after 8 min (colored circles) for all [K^+^]_o_ conditions (note that the circles for 5.4 mM and 4 mM overlap). **(D)** shows how the changes to V_r_ develop over time, again showing a dramatic increase at around 140 s for 2.5 mM. **(E)** shows the charge carried into and out of the cell by Na^+^ ions during each pacing cycle (AP and diastolic interval). The blue line in each graph indicates I_NaK_, the only outward Na^+^ flux. The orange, green, and red shaded areas shows the influx due to I_NaCa_, I_NaB_, and I_Na_, respectively. The left-most graph illustrates the stable 5.4 mM situation, in which the system is in balance and the sum of inward fluxes equals the outward flux through I_NaK_. The second panel (4 mM) shows an instantaneous reduction in inward flux (through reduced I_NaCa_), and a greater reduction in outward flux (through reduced I_NaK_). Over time, this causes the [Na^+^]_i_ changes seen in **(B)** until the system restabilizes. The dominant instantaneous effect in the third graph (3.2 mM) is a decrease in Na^+^ influx through I_Na_. Again the Na + /K + pump adjusts to compensate and rebalance the system, but now at a reduced [Na^+^]_i_ and somewhat depolarized V_r_. Finally, in the (2.5) case the gradual depolarization of V_r_ grows until no I_Na_ is available for an I_Na_-driven regenerative AP. These mechanisms are described in further detail in the text and in Figure 6.

Slowly-developing effects (minutes) in cell electrophysiology settings may occur through changes in intracellular electrolyte balances. Since the Na^+^/K^+^ pump is a determinant of the internal sodium concentration ([Na^+^]_i_) as well as being sensitive to small [Na^+^]_i_ changes, we investigated the role of [Na^+^]_i_ in the observed long-term changes. Our results are shown in Figure 4, which illustrates the time-dependent changes during the first 8 min after the change in [K^+^]_o_. At the end of this interval the system was seen to stabilize.

Action potentials either 1 s or 8 min after the [K^+^]_o_ change are shown in Figure 4A. The changes in V_r_ are very similar to those illustrated in earlier figures. Figure 4B shows the changes in [Na^+^]_i_ during the 1 Hz AP train, from the start of the simulation (including the very first AP before the graph in Figure 4A). There is a qualitative difference between the response to a small decrease (5.4 to 4 mM), where [Na^+^]_i_ increases, and the larger decreases (to 3.2 and 2.5 mM), where [Na^+^]_i_ *decreases*. A secondary change is seen to occur around 140 beats, when the rate of the progressive decrease in [Na^+^]_i_ suddenly accelerates. The insets in Figure 4B show an AP before and after this time point, indicating a transition from a “normal” AP morphology to an small depolarization, resembling a “subthreshold response.” The basis for this sudden and significant change can be seen in the next panels. Figure 4C consists of two plots of the steady-state voltage-dependence of I_Na_ inactivation (gray lines). These curves are the same in both plots, and were obtained by multiplying the corresponding relationships for slow and fast inactivation together. The color-coded circles indicate the location of V_r_, 1 s and 8 min after the change to [K^+^]_o_, showing whether I_Na_ is available or inactivated at rest. As can be seen, I_Na_ availability is almost entirely unaffected for the change to 4 mM. In contrast, the depolarized V_r_ at lower [K^+^]_o_ values causes a significant reduction in the amount of available I_Na_. As Figure 4D shows, the progressively depolarizing V_r_ for these hypokalemic conditions leads to a slowly decreasing amount of available I_Na_. In the 2.5 mM [K^+^]_o_ experiment, this ultimately results in a loss of inward I_Na_ current and a failure to depolarize or generate an AP.

Finally, Figure 4E shows the effects of the changes in [Na^+^]_i_ and I_NaK_ on the Na^+^ homeostasis in the atrial myocyte. The left-most column shows the situation when [K^+^]_o_ is unchanged and the system is in balance. The orange, green, and red shaded areas show the charges carried into the myocyte by Na^+^ influx during each pacing cycle (AP and diastolic interval) for the Na^+^/Ca^2+^ exchanger (I_NaCa_), the background Na^+^ current (I_NaB_), and the fast Na^+^ current (I_Na_). At steady-state, the sum of these three Na^+^ influxes is balanced by the efflux of Na^+^ through I_NaK_ almost exactly (the model contains a separate slowly activating Na^+^ current, I_NaL_, omitted here for clarity) so that the blue line for I_NaK_ and the red line for the sum of the inward fluxes overlap.

The second column in Figure 4E shows effects of a sudden [K^+^]_o_ change from 5.4 to 4 mM, to which the Na^+^/K^+^ pump can adequately respond: While the immediate effect is a reduction of I_NaK_ (the blue line dips), this triggers a feedback response wherein reduced I_NaK_ causes an [Na^+^]_i_ accumulation which increases I_NaK_ until the system is once again in balance (the blue and red lines overlap).

A perhaps more unexpected effect is seen in the third column of Figure 4E, which illustrates the restoration of Na^+^ balance after a step change to 3.2 mM [K^+^]_o_. Recall from Figure 3 that the immediate effects of this step-change include a reduction of I_K1_ and a depolarization of V_r_. In this setting, the dominant and immediate effect is the reduction of Na^+^ influx through I_Na_, due to the reduced I_Na_ availability produced by the depolarization of V_r_. Again the Na^+^/K^+^ pump, acting as one element in a [Na^+^] homeostasis feedback mechanism, reacts to restore the balance. In this setting, reducing pump current and Na^+^ efflux, leads to the slow progressive increase in V_r_ seen in Figure 4D.

Finally, the fourth column of Figure 4E shows the effects of a reduction to 2.5 mM [K^+^]_o_. Initially, effects are similar to the 3.2 mM case: I_K1_ decreases, leading to V_r_ depolarization and a large and rapid decrease in Na^+^ influx through I_Na_; followed by a steady decrease in [Na^+^]_i_ as the Na^+^/K^+^ pump reduces its efflux to match. In this scenario, however, the resulting V_r_ depolarization is so large that AP excitation fails, causing the significant shifts seen starting around 100 beats. Although Na^+^ balance is eventually restored the atrial myocyte has lost its excitability.

While we did not repeat this detailed analysis for all seven models, very similar patterns of results can be seen in the other models in Supplementary Figures 11–17. Changes to [Na^+^]_i_ differ in direction according to the size of the [K^+^]_o_ decrease, and there is often a sudden qualitative change a few minutes into the experiment. As expected, the models without [K^+^]_o_-sensitive I_K1_ rectification show a very different, monophasic [Na^+^]_i_-response. This monophasic response is also shown by the model by Maleckar et al., which hyperpolarizes at 3.2 and 2.5 mM [K^+^]_o_ (see Supplementary Figure 10).

### Strand Simulations

The third part of this study consisted of a series of computations aimed at revealing some of the important contributing factors for either (i) triggering atrial arrhythmias or (ii) producing long-lasting electrophysiological changes in multicellular atrial syncytium (often referred to as the substrate) in the setting of hypokalemia. It is known that both hyper- and hypo-kalemia are associated with initiation or prolongation of rhythm disturbances in both the ventricles and atria of mammalian hearts (Gettes et al., 1962; Weiss et al., 2017). For these studies, a model system (described in detail in section “Materials and Methods”) was formulated on the basis of a one-dimensional strand of 200 identical Voigt et al. (2013) human atrial myocyte models. Stimulus protocols (described in section “Materials and Methods”) and analyses were applied to determine the dynamics of the FRP, alterations in CV and related changes in conduction WL. Simulations were stopped at either 1 s or 15 min following changes to the same levels of [K^+^]_o_ that were used previously in single myocyte studies (Figures 1–4). A 1 Hz stimulus train was applied.

**FIGURE 5:**
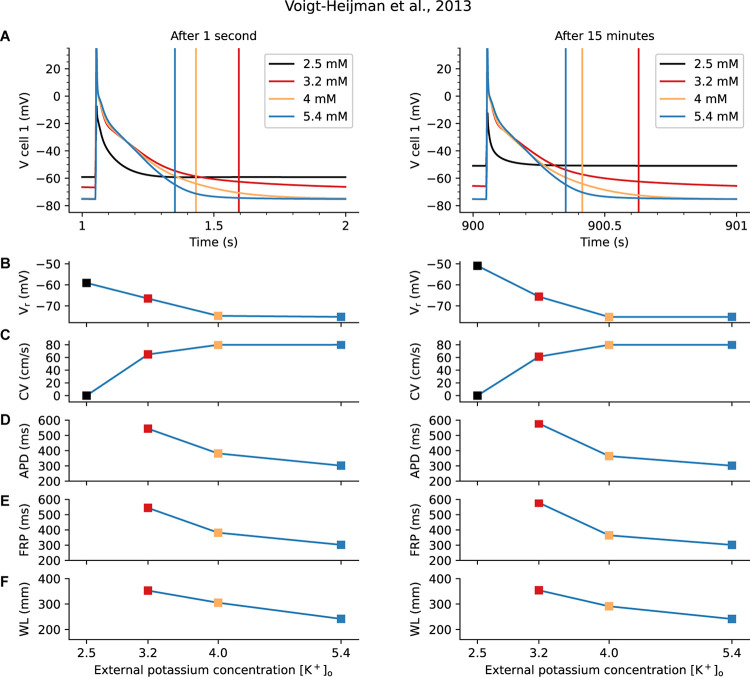
Human Atrial Tissue “Strand” Model for Evaluation of Effects of Hypokalemia on Excitability, Conduction Velocity and Wave Length of Conduction. The Voigt-Heijman et al. model of the human atrial myocyte was used as the basis for constructing a 1-D, 200 myocyte atrial strand. Standard steady-state pacing and extra stimulus protocols (S_1_–S_2_) were applied, and the results are shown either immediately (1 s) after altering [K^+^]_o_ or under quasi steady-state conditions (after 15 min). The separate panels in **Row A** show individual AP waveforms in the first myocyte in the simulated strand, at the [K^+^]_o_ levels that were the focus of this study. The vertical lines in these graphs indicate the functional refractory periods (FRP) that were estimated from the tissue strand response after either 1 s (left) or 15 min (right). These results, and the corresponding V_r_, CV, APD, and WL values are presented in **Rows B–F** below. Note that in 2.5 mM [K^+^]_o_ the small AP failed to support conduction, and so no APD, FRP, or WL could be determined. See section “Results” for more details.

The design of these studies and most aspects of the data analysis were guided by an extensive experimental study concerning the effects of [K^+^]_o_ on rabbit atrial tissues by Smeets et al., 1986. Results obtained at 1 s and 15 min after [K^+^]_o_ changes are illustrated in Figure 5. In Figure 5A the V_r_ values and AP waveforms formed in response to selected [K^+^]_o_ levels are shown as measured in the first myocyte in the simulated human atrial tissue strand. The vertical lines indicate the FRP at each [K^+^]_o_ level. At 2.5 mM [K^+^]_o_ the atrial tissue strand generates only a very small regenerative AP. It fails to support propagation/conduction, so that no FRP can be shown. The reasons for this discrepancy were not pursued in detail. The complete results of these simulations are summarized in Figures 5B–F, in terms of: the resting potential **(5B)**, CV **(5C)**, and (where possible) the APD **(5D)**, FRP **(5E)**, and WL **(5F)**. In agreement with the Smeets et al. study, the CV is decreased at lower [K^+^]_o_ levels, in both the 1 s and 15 min settings. However, the observed elongation of the APD in these simulations leads to a much increased FRP at low [K^+^]_o_, resulting in an increased WL of conduction. This finding is in contrast to the decrease in WL reported by Smeets et al.

Complementary results in generated by the other models are shown in Supplementary Section 6. Since the Voigt-Heijman et al. model results after 15 min did not differ qualitatively from the results after 1 s, we did not run the computationally expensive 15 min fiber simulations for all models. As before, the results differ dramatically for the models with and without [K^+^]_o_-dependent I_K1_ rectification, which show decreases in both APD and FRP. We note, however, that none of the models tested showed the expected prolonged APD with FRP shortening. Finally, results relating to single-cell excitability are shown in Supplementary Section 7.

## Discussion

The computational work in this paper has illustrated how hypokalemia can lead to an increased deviation of V_r_ from the potassium reversal potential, even resulting in depolarization at lower [K^+^]_o_. To produce this effect, models of the AP and V_r_ under hyper- or hypokalemic conditions *must* include the three [K^+^]_o_ effects on the inwardly rectifying background K^+^ current, I_K1_, which is expressed in both the left and right atrium of mammalian hearts (Voigt et al., 2010). In the human atrium, I_K1_, is generated mainly by the K^+^ channel transcripts, Kir2.1 and Kir2.3 (Melnyk et al., 2002). These K^+^ transcripts, alone or in combination, can sense and react to changes in [K^+^]_o_ with high affinity and very rapid response times. Recognition and understanding of these properties provides the basis for understanding the direct and indirect effects of clinically-relevant hypokalemia (Macdonald and Struthers, 2004; Krijthe et al., 2012; Mehta and Emmett, 2017). Specifically, validated mathematical models must be able to accurately reproduce the [K^+^]_o_-dependent modulation of the highly non-linear I-V relationship (voltage-dependent rectification) generated by “strong inward rectifier” transcripts such as Kir2.1/2.3 (Lu, 2004; Dhamoon and Jalife, 2005; Anumonwo and Lopatin, 2010; Hibino et al., 2010) in all myocytes from human atrium (Figure 2) and ventricles (Bouchard et al., 2004; Trenor et al., 2018).

**FIGURE 6:**
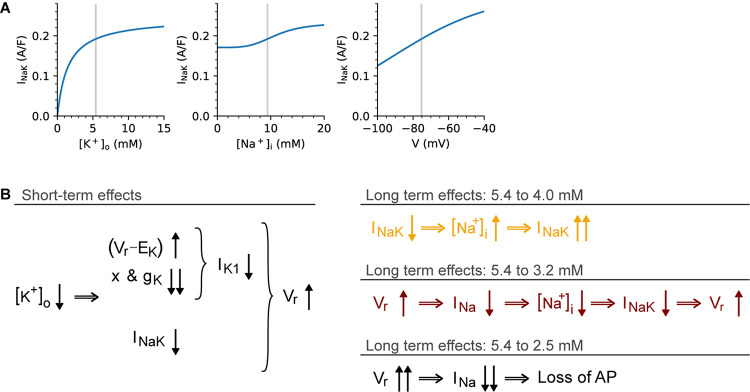
Qualitatively different long-term effects of different [K^+^]_o_ changes. This diagram summarizes the origins of the contrasting short- and long-term effects seen in Figure 4. **(A)** illustrates the sensitivity of I_NaK_ to [K^+^]_o_ (left), [Na^+^]_i_ (center), and V_r_ (right), as determined from the Voigt-Heijman model under baseline conditions. The schematic in **(B)** shows the immediate (left) and delayed (right) effects of [K^+^]_o_ changes. The short-term effects are similar in all cases: a step change in [K^+^]_o_ causes an increased electrochemical driving force for I_K1_ at potentials near V_r_, this causes an increase in I_K1_. However, this effect is overshadowed by the I_K1_ decrease due to the effects of [K^+^]_o_ on I_K1_ rectification and maximum conductance. In response to lower [K^+^]_o_, I_NaK_ shows an instantaneous decrease, and together these effects cause a depolarization of V_r_ in all tested [K^+^]_o_ levels. In the long-term, however, the effect is size-dependent: For small decreases (5.4 to 4 mM), the effect is dominated by the initial reduction in I_NaK_, which leads to an increased Na^+^ influx. This, in turn, causes [Na^+^]_i_ to rise progressively and I_NaK_ to increase until Na^+^ balance is restored. For larger decreases in [K^+^]_o_ (5.4 to 3.2 mM) the effects of V_r_ depolarization on I_Na_ “availability” become dominant. The depolarized V_r_ reduces I_Na_ availability, so that both peak current and Na^+^ influx through I_Na_ is decreased, causing a reduction in [Na^+^]_i_. This in turn further reduces I_NaK_ until its Na^+^ efflux once again matches the (reduced) influx. In this case, balance is restored, but at a depolarized V_r_ level, which markedly reduces excitability. Finally, for the largest reduction in [K^+^]_o_ studied (5.4 to 2.5 mM), this same pattern develops, but at a faster rate until the depolarization of V_r_ reaches a point where I_Na_ availability is too low to support an I_Na_-driven depolarization.

Based on our application of five models that include [K^+^]_o_-dependent I_K1_ rectification, we saw that even small decreases in [K^+^]_o_ can very rapidly produce a significant lengthening of the AP duration. This effect occurs almost immediately (within 1 s or 1 heartbeat), and is mediated mostly through I_K1_ and V_r_. At both the single myocyte level and in our 200 myocyte 1-D strand of atrial tissue, this hypokalemia-induced change in the AP waveform significantly lengthened the refractory period and the WL for conduction (Figure 5). A further insight gained from our simulations is that even small decreases in [K^+^]_o_, e.g., 4 to 3.2 mM; or 3.2 to 2.5 mM can alter the outward electrogenic current produced by the Na^+^/K^+^ pump, and that this interacts with V_r_, [Na^+^]_i_, and I_Na_ in ways that are predictable in the short term (seconds), but much more complex in the longer term (minutes). This was explored in detail in Figure 4. Notably, qualitatively different effects on the AP and V_r_ were observed when changing [K^+^]_o_ from 5.4 mM to 4, 3.2, and 2.5 mM, as summarized in Figure 6. While the analysis in Figures 4, 6 is more strongly model-dependent than our previous results, we believe they show a pattern of changes that is shared by other models that include [K^+^]_o_-dependent I_K1_ rectification, as can be seen in Supplementary Figures 11–17.

Finally, we showed that these effects persist in strands of connected myocyte models (Figure 5), although we did not observe the reduced refractory period seen with prolonged APD by, e.g., Smeets et al. (1986).

### Relationship to Previous Basic Science Studies

The significant physiological and pathophysiological effects in the mammalian heart of changes in plasma K^+^ are the basis of previous mechanism-oriented studies of both hypo- and hyperkalemia (Gettes et al., 1962; Kléber, 1983; for review see Weiss et al., 2017). We briefly discuss how our work fits in with earlier work, starting with the relationship between V_r_ and [K^+^]_o_.

A common line of reasoning holds that the resting potential in atrial myocytes is dictated by the reversal potential for potassium ions, E_K_, and is strongly regulated I_K1_. But it has also been well established that V_r_ increasingly deviates from E_K_ as [K^+^]_o_ is lowered from hyperkalemic to normo- and hypokalemic conditions. For example, Figure 4 in a study by Vaughan Williams (1959) shows an increasing deviation from E_K_ in rabbit ventricle, starting at values as high as 6 or 7 mM. Similar examples in human atria can be seen in Figure 2 of Gelband et al. (1972) and Table 1 of McCullough et al. (1987). Ten Eick and Singer (1979) showed depolarization at 2 mM for healthy human atrial cells, an effect which was already present at 4 mM for diseased (partially depolarized) cells. Figures 2, 3 in McCullough et al. (1990) show a depolarization when lowering [K^+^]_o_ from 7 to 4 mM in cells from diseased human ventricle, and perform experiments to show that these cells can exist at one of two stable states (with a V_r_ of either −80 or −45 mV). Recent work by Ma et al. (2011) provides further review of what they call “paradoxical depolarization,” and later work by the same group links this to Kir channels (Zuo et al., 2017).

In our Supplementary Figure 10 we show predictions from all seven models for the relationship between V_r_ and [K^+^]_o_. We demonstrate that all human atrial models that incorporate [K^+^]_o_-dependent I_K1_ rectification predict this effect – although at a variety of potassium levels, with most models predicting a depolarization starting at approximately 4 mM. Howewver, the model by Maleckar et al. stays closer to E_K_ until approximately 1.5 mM. Interestingly, the models predict a relatively smooth V_r_-[K^+^]_o_ relationship after 1 s of hypokalemia, but after 15 min most models display a sharp cut-off instead, corresponding to the observation of two stable V_r_ levels in, e.g., Gelband et al. (1972) and McCullough et al. (1990). This variability in model predictions may be expected based on Supplementary Figure 3, which displays the relative contribution of ionic currents at V_r_. In most models, this consists of I_K1_ and I_NaK_ (outward), balanced by the (inward) Na^+^/Ca^2+^-current I_NaCa_ and several “background currents.” Compared to, e.g., I_Kr_ and I_Na_ these currents have perhaps not received as much attention as they warrant from the modeling community, which is further corroborated by the fact that both the I_K1_ and I_NaK_ formulations in all models used in this study date back to the early or mid 1990s. This has been partly ascribed to the difficulty of measuring these relatively small “diastolic” currents (Bueno-Orovio et al., 2014). In this respect, it would be useful to perform new experiments measuring V_r_ under different hypokalemic conditions, e.g., in right-atrial appendage samples and perhaps in the presence of pharmaceutical blockers such as Ouabain. Such experiments could resolve uncertainties regarding the V_r_-[K^+^]_o_ relationship we highlighted in this study, but also have the potential to add to the relatively sparse data record on the balance between I_NaK_ and other diastolic currents.

Many previous studies have interpreted both short- and long-term effects of low [K^+^]_o_ on cardiac muscle mainly in terms of its established inhibitory effects on the Na^+^/K^+^ pump (Aronsen et al., 2015; Weiss et al., 2017). This interpretation is logical since it is known that an essential regulatory site on the external surface of this integral membrane protein complex binds K^+^ with high affinity (Glitsch, 2001). Thus, a decrease in [K^+^]_o_ to 3.2 mM from 4 or 5.4 mM can significantly reduce the activity of the Na^+^/K^+^ pump, and therefore result in a gradual increase in [Na^+^]_i._ This change in the electrochemical driving force for Na^+^ alters Na^+^/Ca^2+^ exchange activity and can produce an increase in [Ca^2+^]_i_ (Pogwizd et al., 2001; Matchkov et al., 2007). Maintained increases in [Ca^2+^]_i_ can contribute to a proarrhythmic substrate by a variety of ionic mechanisms. These are likely to include: (i) inhibitory effects of [Ca^2+^]_i_ on Na^+^ channels (Chadda et al., 2017; Huang, 2017) and/or (ii) on the background K^+^ current, I_K1_ (Nagy et al., 2013) in the mammalian heart. Changes in [Ca^2+^]_i_ can also alter myocyte-to-myocyte electrotonic communication by changing the conductance of the connexins in gap junctions (Matchkov et al., 2007; Varró et al., 2020). It is also important to note that changes in V_r_ in response to alterations in [K^+^]_o_ can strongly modulate the Na^+^/Ca^+^ exchange current and thus alter the AP waveform (Baczkó et al., 2003) and myocyte contractility (Ng et al., 1987; White and Terrar, 1991).

In contrast, not all of the prominent low [K^+^]_o_ effects that we have identified can be fully explained by Na^+^/K^+^ pump-based mechanisms. For example, after the change to low [K^+^]_o_, the AP lengthening occurs almost instantaneously. Therefore, this effect can not be due to a partial inhibition Na^+^/K^+^ pump activity. Based on our computational analysis, we agree, however, that at later times after small reductions in [K^+^]_o_ the electrogenic current generated by the Na^+^/K^+^ pump does indeed have an important effect on excitability and perhaps on CV in atrial myocytes and tissue (see Figure 5). As mentioned, this effect is due to a small, but significant, increase in [Na^+^]_i._ The exceptional sensitivity of all known isoforms of the Na^+^/K^+^ pump to changes in [Na^+^]_i_ within the physiological range (approximately 9–11 mM) can alter the associated electrogenic current, and thus change diastolic membrane potential, V_r_ (Glitsch, 2001). This effect appears to be quite prominent in human atrium (Sanchez et al., 2012).

We also acknowledge that in accordance with an essential part of the published working hypothesis for the effects of hypokalemia, there would be expected to be an increase in [Ca^2+^]_i_. However, additional and more detailed analysis of Ca^2+^ homeostasis in the human atrial myocyte is needed to reveal whether this is due to the I_K1_-induced broadening of the AP waveform and related effects on dynamics of the L-type Ca^2+^ current (Clark et al., 1996; Sah et al., 2003; Bouchard et al., 2004); altered release of Ca^2+^ from the sarcoplasmic reticulum (Koivumäki et al., 2011); a change in the ability of the Na^+^/Ca^2+^ exchanger to extrude Ca^2+^ (Pogwizd et al., 2001; Baczkó et al., 2003); or a combination of these effects.

Several studies have addressed the effects of [K^+^]_o_ on CV, or on the maximum upstroke velocity V̇_max_ which is known to correlate with CV. Early studies observed a “biphasic” relationship between [K^+^]_o_ and CV, in which CV peaked at around 9–10 mM in mammalian ventricular cells (Kagiyama et al., 1982; Buchanan et al., 1985) and near 4 mM in sheep Purkinje cells (Dominguez and Fozzard, 1970). Similarly, V̇_max_ was found to decrease with hyperkalemia, but remained relatively unchanged in hypokalemic conditions. All three studies reported a hyperpolarization of V_r_ with reduced [K^+^]_o_. Although effects of hyperkalemia could be explained quite readily by the depolarization of V_r_ and resulting inactivation of I_Na_; the effects of lowering [K^+^]_o_ were more of a puzzle. In this respect it is interesting to note the work of Kishida et al. (1979), who showed that the effects of [K^+^]_o_ are partially voltage-independent, and the follow-up work by Whalley et al. (1994) who demonstrated that the effects of [K^+^]_o_ on CV can be altered significantly by application of sub millimolar concentrations of BaCl_2_, a well-known and selective blocker of I_K1_ (Whalley et al., 1994).

Consistent with this literature, our strand simulations show a decrease in CV with hyperpolarization. Previous work by Nygren and Giles (2000) also showed a peak in CV at around 8 mM. Single-cell results on V̇_max_ are shown in Supplementary Figure 21. In contrast to the experimental studies, these show a “biphasic” response similar to the CV experiments. Interestingly, this effect occurs regardless of whether the models hyperpolarize or depolarize at lowered [K^+^]_o_. The Courtemanche et al. and Ni et al. models both hyperpolarize at low [K^+^]_o_, leading to a very slight increase in I_Na_ availability (Supplementary Figure 23) but their lack of [K^+^]_o_-dependent I_K1_ rectification also causes a significant increase in I_K1_ (Supplementary Figures 13, 14) which as expected reduces both CV and V̇_max_. Conversely, the four models with [K^+^]_o_-dependent I_K1_ rectification that *depolarize* at low [K^+^]_o_ all show a strong reduction in available I_Na_, leading to a reduced CV and V̇_max_ in spite of the reduced I_K1_. The most interesting model in this respect is the one by Maleckar et al., which has [K^+^]_o_-dependent I_K1_ rectification but still hyperpolarizes and so is most similar to the ventricular data sets described above. In this model, the slight hyperpolarization causes a small increase in I_Na_ availability only when stepping from 5.4 to 4 mM [K^+^]_o_ (Supplementary Figure 24), while I_K1_ increases further at 3.2 and 2.5 mM (Supplementary Figure 16). This is likely to be part of the reason why, in this model, although CV keeps decreasing at more negative potentials (Supplementary Figure 20) the V̇_max_ response flattens below 4 mM.

### Relationship to Previous Clinical Findings

Our simulations illustrate how even moderate hypokalemia leads to a reduction in I_K1_, and a resulting increase in APD. This reduction of the atrial myocyte’s ability to stabilize at V_r_ implies an increased susceptibility to variability in the late stages of repolarization and therefore an increased susceptibility to variability in APD. In human atrium (Maleckar et al., 2009b) and in most other mammalian cardiac preparations, variability in APD is known to contribute to initiation and maintenance of atrial rhythm disturbances (Hondeghem et al., 2001; Kim et al., 2002; Fabritz et al., 2003). In fact, APD alternans often precedes initiation of atrial rhythm disturbances in patients (Narayan et al., 2011; Huang, 2017). This reduction in stability at V_r_ is further explored in Supplementary Section 8 where we show a reduction in [K^+^]_o_ leads to an increase in membrane resistance at rest and in the late stages of repolarization, so that small fluctuations in current at this stage can cause large changes in the membrane potential (and thereby in the APD).

There is a general consensus that the [K^+^]_o_ levels in a healthy adult human are closer to 4 to 4.5 mM than the 5.4 mM value typically used in wet lab and simulation experiments (Macdonald and Struthers, 2004; Mehta and Emmett, 2017) and that quite small changes (±1 to 2 mM) can confer a proarrhythmic phenotype in mammalian atria (Zaza, 2009; Krijthe et al., 2012). The associated hypo- or hyperkalemic phenotypes are observed quite frequently in clinical settings and must be monitored and managed effectively. A primary example is the need for optimization of the use of diuretics, and related transient or maintained alterations in [K^+^]_o_. In clinical settings, such as management of hypertension this often results in hypokalemia and related atrial rhythm disturbances (Franse et al., 2000; Zaza, 2009; Wang et al., 2013; Krogager et al., 2020). In addition, patients in the postoperative period following coronary bypass, or in the setting of heart failure, can also present with hypokalemia and associated electrophysiological instability of their atria and ventricles (Ahmed et al., 2007; Barrett Bowling et al., 2010; Severi et al., 2010; Collins et al., 2017). Perhaps the most common situation in which plasma K^+^ is actively manipulated in clinical settings and when it can decrease to hypokalemic levels is during renal dialysis (Genovesi et al., 2008; Zimmerman et al., 2012). It is recognized that some aspects of atrial rhythm disturbances are strongly associated with clinical hypokalemia (Vincenti et al., 2014).

Basic and clinical cardiac electrophysiologists have long attempted to detect and understand [K^+^]_o_-induced rhythm disturbances in mammalian heart. This has been done by monitoring refractoriness, CV and AP duration (Smeets et al., 1986; Nygren and Giles, 2000; Killeen et al., 2007b). Analysis often involves relating these factors in terms of the WL for conduction-that is, the product of CV and AP duration (Jacquemet et al., 2005; King et al., 2013a). Quite recently, based on detailed monophasic AP recordings from mouse hearts, this analysis paradigm (changes in WL for conduction and its restitution) has been updated and placed in a semi quantitative context (Sabir et al., 2007, 2008; Matthews et al., 2013). In brief, this comprehensive analysis provides a relationship between conduction WL restitution and AP duration alternans in the setting of hypokalemia (Killeen et al., 2007a, b). An important underlying physiological principle is that alterations in (i) the amount of inactivation (availability) of the Na^+^ current, (ii) its reactivation during the diastolic period, and (iii) a progressive inhibitory effect on I_Na_ of increases in [Ca^2+^]_i_ (King et al., 2013b; Chadda et al., 2017; Huang, 2017) combine to contribute to a proarrhythmic substrate.

The dynamics of I_Na_ are particularly important in mammalian atria, given that its resting potential is relatively depolarized (e.g., −75 mV) compared to that in the ventricle. As a result, much of this essential rapidly activating inward current is not available or inactivated at diastolic membrane potentials (Sakakibara et al., 1992; Whalley et al., 1994). In fact, a recent study of [K^+^]_o_-induced atrial rhythm disturbances concludes that alterations in I_Na_ that result in its repetitive activation is a major proarrhythmic mechanism (Tazmini et al., 2020). In addition, Pandit and Jalife (2013), when reviewing mechanisms that underlie fibrillation, have suggested that functional linkages of I_K1_ and I_Na_ are likely to be essential for rotor formation and dynamics. Recent work by Sossalla et al. (2010) has shown a significant decrease in peak I_Na_ (but an increase in its late component) in isolated atrial myocytes from patients with AF, which could be further exacerbated by [K^+^]_o_-dependent I_Na_ decrease.

As mentioned previously, increases in [Ca^2+^]_i_ have also been reported to be able to decrease K^+^ currents that are carried by I_K1_ (Nagy et al., 2013). In fact, much earlier studies of the relationship between [K^+^]_o_ and I_Na_ had also drawn attention to K^+^-induced changes in membrane potential and related alterations in the availability and dynamics of the Na^+^ current in atrial tissue (Whalley et al., 1994; Spach et al., 2007).

### Future Considerations

This study was performed to address a specific question and therefore has only a quite limited scope. To put our findings in a broader context, it is interesting to consider other published findings that also provide insights into how I_K1_ can change the human atrial electrophysiological substrate. For example, it is now known that mammalian atrial myocytes include a functional transverse tubule system (Richards et al., 2011; Tazmini et al., 2020). This finding, coupled with previous demonstrations that the K^+^ channels that are responsible for I_K1_ show prominent expression in the transverse tubule system (Clark et al., 2001), give rise to the possibility that in the failing heart (Lichter et al., 2014) a fraction of I_K1_ channel expression is lost due to disease-related decreases in T-tubule density. By analogy to what is known about skeletal muscle, this decrease in I_K1_ density could destabilize the resting potential and perhaps also alter excitability (Riisager et al., 2014). This change may be particularly relevant during relatively high frequency firing that is characteristic of chronic AF (Fraser et al., 2011, c.f. Tazmini et al., 2020).

Functionally significant, reversible changes in [K^+^]_o_ have also been demonstrated in the mammalian cardiovascular system in the setting of maintained exercise (Paterson, 1997). One of the factors responsible for the association of exercise for endurance training with the incidence of AF may be explained by the striking sensitivity and rapid responsiveness of I_K1_ to small changes in [K^+^]_o_, and related effects on AP waveform and refractoriness that are revealed by our simulations (Figures 1, 5).

The antiarrhythmic drug flecainide has also been shown to enhance a background K^+^ current that is generated by a subset of the Kir2.X isoforms that produce I_K1_ (Caballero et al., 2010). This effect may be a significant factor in some of the proarrhythmic actions of flecainide both in ventricle and in supraventricular tissues. Somewhat similarly, nitric oxide can enhance I_K1_ expression in cardiac tissue (Gómez et al., 2009). Thus, changes in intrinsic tissue metabolism, including alterations in intracellular phospholipid levels such as PIP_2_ (Lopatin and Nichols, 1996) may be an important cofactor to consider in future studies concerning the [K^+^]_o_ dependence of atrial physiology and pathophysiology (Gómez et al., 2009). In particular, details of the negative slope region of the I_K1_ I–V curve will need to be studied in detail (Baronas and Kurata, 2014).

Initiation and maintenance of atrial rhythm disturbances is known to be modulated by enhanced production of free radicals that are generated, e.g., during a sterile inflammatory response (Coppini et al., 2019; Varró et al., 2020). This finding may be related to the pattern of results revealed by our computations in two ways: (i) It is known that Na^+^/K^+^ pump activity is reduced significantly by this type of “redox challenge” (Figtree et al., 2012) and to free radical production and in particular enhanced generation of hydrogen peroxide can increase the slowly inactivating or late component of the Na^+^ current (Ward et al., 2006; Pezhouman et al., 2018). Both of these effects would be expected to destabilize the resting potential, and may be proarrhythmic through their ability to reduce the net outward current in diastole.

Finally, it is important to recognize that atrial tissue does *not* consist only or even mainly of myocytes. Rather, both atria in mature mammalian hearts include very significant populations of fibroblasts/myofibroblasts that connect, one to another, to form a syncytium. Fibroblasts and myofibroblasts also form connexin-mediated electrotonic interactions with immediately adjacent atrial myocytes. These functional interactions, especially when combined with the fact that these fibroblasts express the background K^+^ current, I_K1_ (Chilton et al., 2005) have important consequences for our main findings and conclusions. Thus, detailed consideration of electrophysiological effects due to altered plasma K^+^ should also consider changes in the atrial myocyte resting potential or AP waveform that are generated in part through electrotonic interactions with fibroblasts (Maleckar et al., 2009a). Consideration of fibroblast/myocyte electrotonic interactions also requires recognition of the possibility that progressive fibrosis as occurs during healthy aging; or localized changes in the strength of myocyte-to-myocyte coupling could contribute to functional alterations and important electrophysiological changes in the human atrium (Spach et al., 2007; Maleckar et al., 2009a; Huang, 2017; Varró et al., 2020).

### Limitations and Future Directions

Atrial rhythm disturbances are the most common cardiac arrhythmias in the aged population (c.f. Tazmini et al., 2020). Accordingly, a number of different approaches have been developed for attempting to detect, manage and possibly even predict episodes of AF. Mathematical simulations of AF are quite advanced, and have already given rise to interesting insights and controversies. Recent reviews summarize available mathematical modeling approaches and also provide opinions concerning “what’s next” (Wilhelms et al., 2013; Vagos et al., 2018; Grandi et al., 2019). Very informative studies of underlying mechanisms and development and refinement of related mathematical models for AF that are directly relevant to the present study have been published (Severi et al., 2010; Krueger et al., 2011; Passini et al., 2014). With further refinement and validation, computational platforms for simulating atrial rhythm disturbances are beginning to make useful contributions Examples include: drug discovery and repurposing (Courtemanche et al., 1999; Koivumäki et al., 2014; Ni et al., 2017, 2020); detailed study of the genetic and molecular causes of arrhythmias (Pandit and Jalife, 2013; Colman et al., 2017; Zhang et al., 2020); and safety pharmacology initiatives (Mirams et al., 2012; Yang et al., 2020). Our findings provide important insights and criteria for selection of robust methods and rational approaches for ongoing or future computational studies of atrial rhythm disturbances.

However, this study also highlights several issues with numerical modeling of atrial cell and tissue electrophysiology which will need to be considered in more detail. In the short-term (1 s) simulations we identified strong interactions between opposing effects: in I_K1_, increases in electrochemical driving force are balanced by changes to its conductance and the voltage-dependence of its rectification. Such a situation, where large opposing effects partially cancel each other to create a smaller effect, requires a highly accurate parameterization. As a result, even small errors in modeling and parameterization of I_K1_ can result in large effects. At diastole, V_r_ is determined by the precise balance between I_K1_, I_NaK_, I_NaCa_, and several “background currents.” Formulations for these currents are typically inherited from older models or added without too much justification, making this an area of AP modeling that requires additional attention.

The longer term effects of hypokalemia were dominated by the interplay of I_K1_, I_NaK_, I_Na_, V_r_, and [Na^+^]_i_. Importantly, even though the changes in [K^+^]_o_ were small, three *qualitatively different* results were seen. This adds to the functional significance of this study, because it shows that such results are *possible* as even a result of very small changes in [K^+^]_o_. However, predicting *exactly which* effects will occur at which [K^+^]_o_ level requires a model that has precisely the right balance between ionic current conductances and that has accurate sensitivities to internal and external electrolytes. Earlier studies have shown that human atrial AP models fall short in this respect (Passini et al., 2014). We have attempted to remedy model-specific effects by repeating the main results in multiple models. However, given the importance of I_K1_ and I_NaK_ in this respect it is worth noting that the seven AP models all inherit these formulations from the same sources, so that one could argue we only really compare 2 or 3 models. Finally, it may not be possible to get the balance between the opposing diastolic currents exactly right without measuring several quantities simultaneously in the same cell – which is technically highly challenging (Whittaker et al., 2020). The complexity of the low [K^+^]_o_ response offers a challenging but also a promising perspective as a relatively simple but highly revealing experimental maneuver to be used in experimental characterization and model building.

## Data Availability Statement

The datasets presented in this study can be found in online repositories. The names of the repository/repositories and accession number(s) can be found below: https://github.com/CardiacModelling/AtrialLowK.

## Author Contributions

MC was responsible for implementation, evaluation, and updates in all of the models of the human atrial action potential that were utilized in this study and carried out all of the simulations and constructed figures. AR and SN provided guidance concerning current clinical investigations of atrial fibrillation, as well as manuscript editing. MC, GM, and WG were involved in the design of the project and wrote the manuscript. All authors have reviewed and approved the final version of this manuscript.

## Conflict of Interest

The authors declare that the research was conducted in the absence of any commercial or financial relationships that could be construed as a potential conflict of interest.
