# Supplementary material for: Immediate and Delayed Response of Simulated Human Atrial Myocytes to Clinically-Relevant Hypokalemia
Source: Front Physiol. 2021 May 26;12:651162. doi: 10.3389/fphys.2021.651162 (PMC8188899; doi:10.3389/fphys.2021.651162)
Supplement: Supplementary file 1 [file Data_Sheet_1.PDF]

Supplement: Immediate and delayed response of simulated human atrial myocytes to clinically relevant hypokalemia

Clerx M, Mirams GR, Rogers AJ, Narayan SM, WR Giles

March 15, 2021

Contents

1 V_r versus $[\mathbf{K}^+]_o$ in human atrial tissue **2**

2 Baseline model comparison **3**
2.1 Action potentials and calcium transients . . . 3
2.2 Relative current contributions . . . 4
2.3 Restitution characteristics . . . 5

3 External potassium in equations for $I_{\mathbf{K1}}$ and $I_{\mathbf{NaK}}$ **8**
3.1 Grandi-Pandit-Voigt and Voigt-Heijman models . . . 8
3.2 Courtemanche and Ni models . . . 11
3.3 Nygren, Maleckar, and Koivumäki models . . . 11

4 V_r versus $[\mathbf{K}^+]_o$: model predictions **14**

5 Immediate and delayed effects of $[\mathbf{K}^+]_o$ changes **15**

6 Strand simulations **22**

7 Single-cell excitability **25**
7.1 Upstroke velocity $\dot{V}_{\mathrm{max}}$ . . . 25
7.2 I_{Na} and strength-duration curves . . . 26
7.3 Membrane resistance estimates . . . 29

8 Single-cell stability **32**
8.1 R_m vs. $[\mathrm{K}^+]_o$ . . . 32
8.2 Quasi-instantaneous I-V relationships . . . 35

References **38**

1 V_r versus $[K^+]_o$ in human atrial tissue

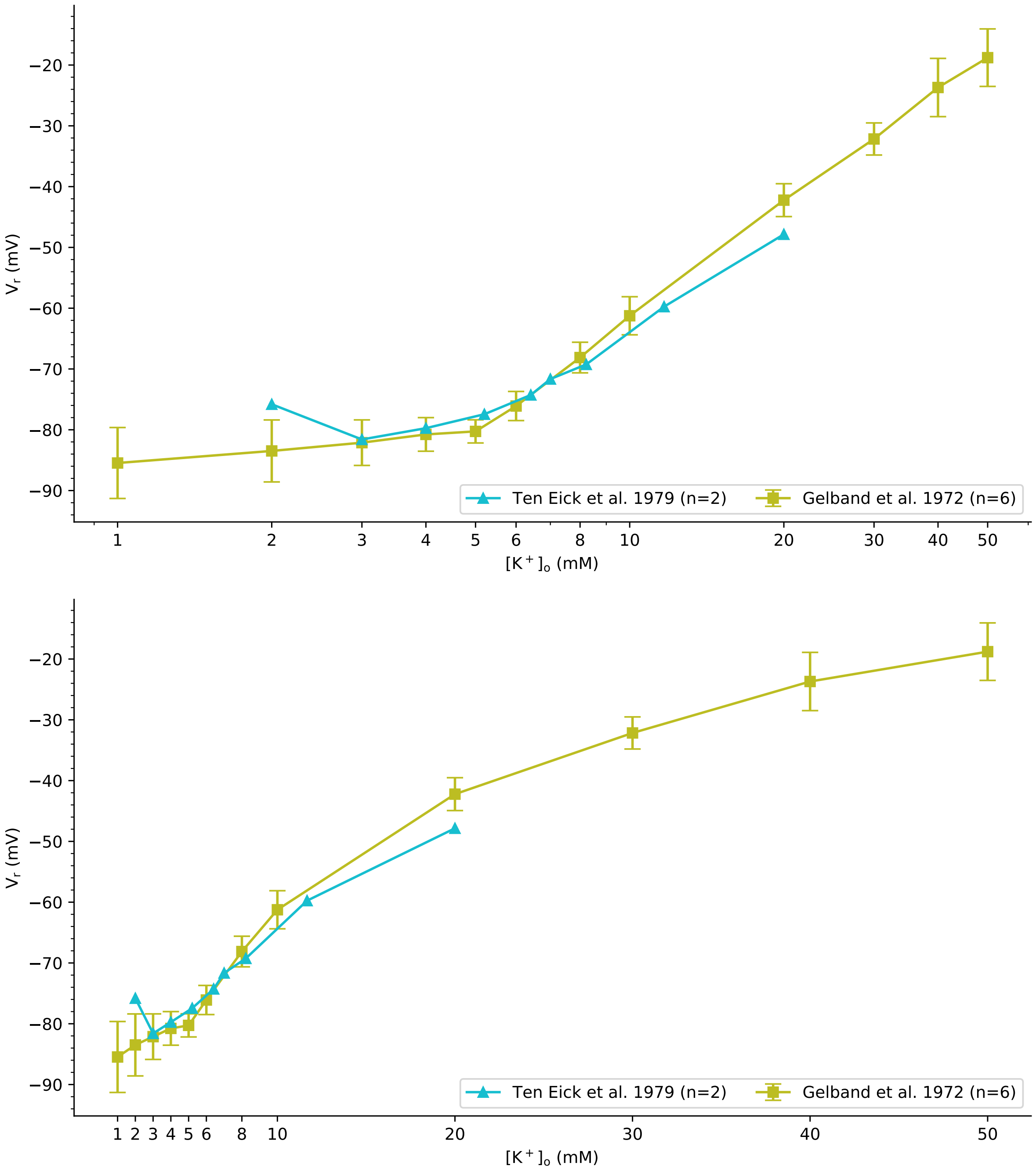

Figure S1: **Resting potential V_r versus external $[\mathbf{K}^+]_o$ levels in excised human atrial tissue**, digitized from the studies by Gelband et al. (1972) and Ten Eick and Singer (1979). The same data are shown with a logarithmic scale on the x-axis (top) and a linear scale (bottom). The Gelband et al. (1972) data was recorded 30 minutes after a change to bath potassium, while the Ten Eick and Singer (1979) experiments were performed "after it was determined that diastolic potential had stabilized", which "usually took no more than 8-10 minutes". Points from Gelband et al. (1972) are shown as mean and standard deviation.

2 Baseline model comparison

The figures in this section show some basic comparisons between the models used in this study. For a much more thorough comparison, see Wilhelms et al. (2013) and Passini et al. (2014).

2.1 Action potentials and calcium transients

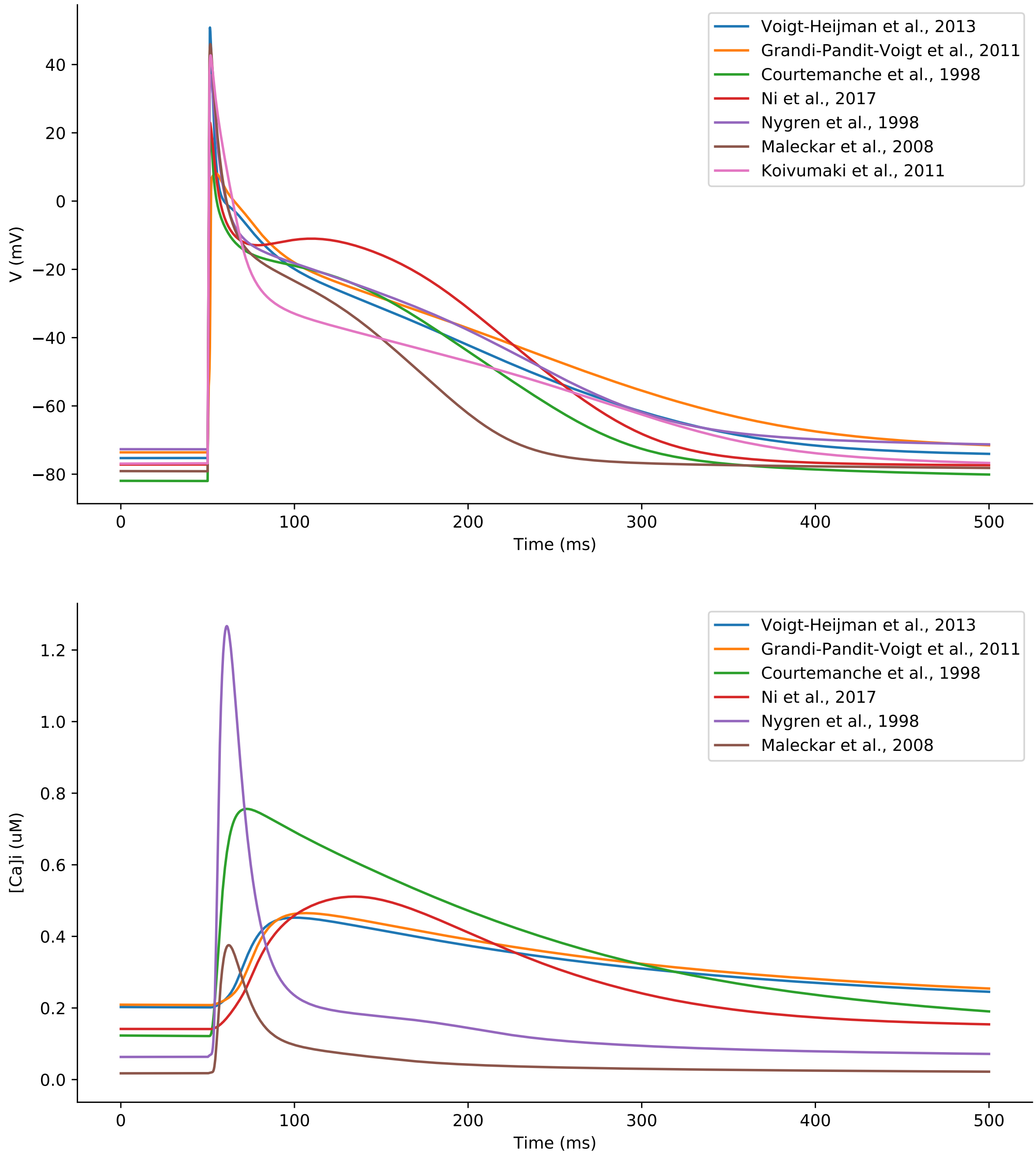

Figure S2: Action potential (AP) and calcium transient (CaT) traces in models of human atrial cell electrophysiology. The model by Koivumäki et al. (2011) has a more complex representation of the internal calcium handling, so that no single "internal calcium" trace can be shown.

2.2 Relative current contributions

The plots below show the 'relative contribution' of the underlying ion currents to the net ionic current. For each selected time t, these plots divide the currents into outward (positive) and inward (negative) components, and then plot the fraction of the total positive or negative current that each current contributes.

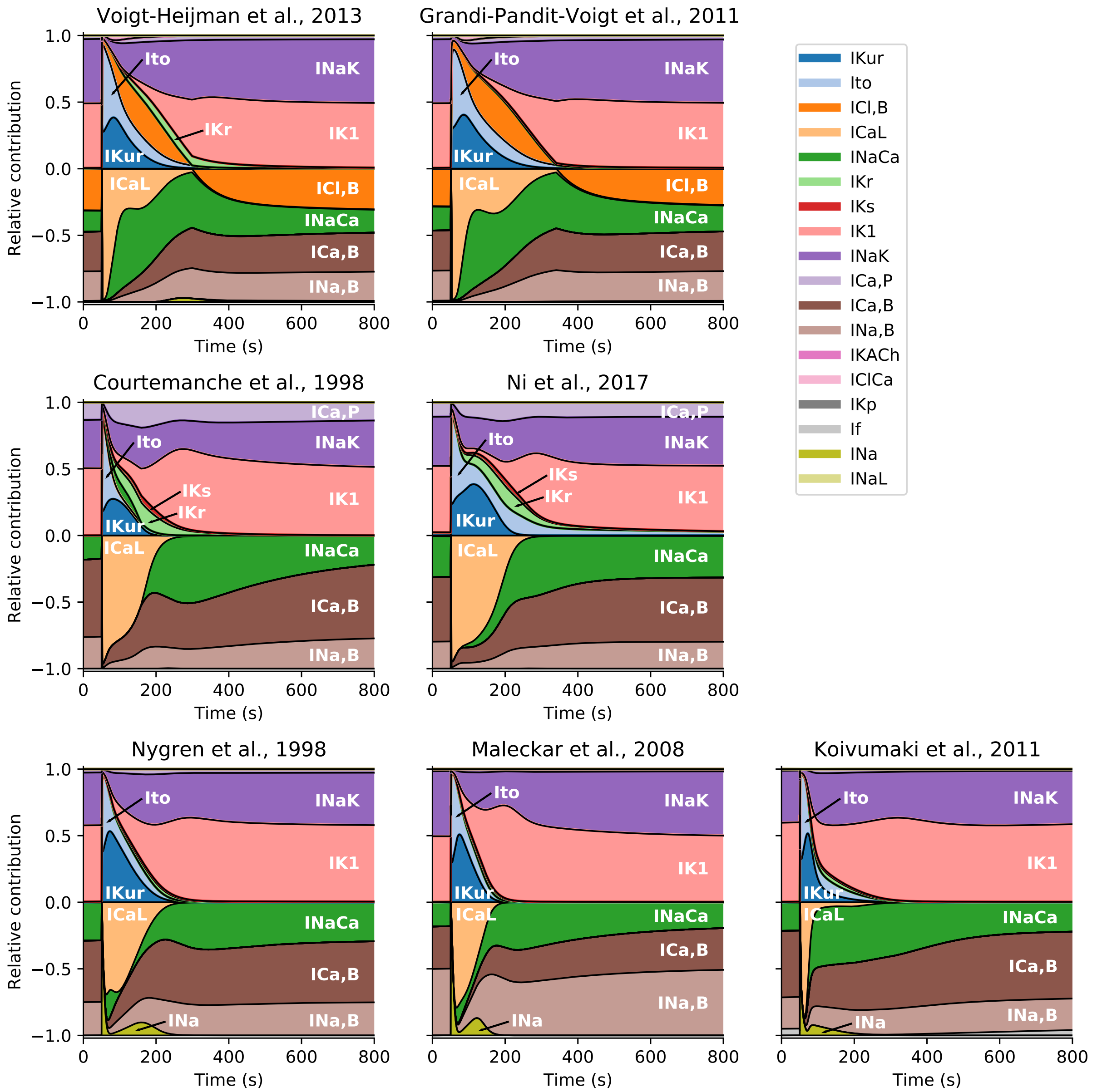

Figure S3: The relative contributions of different currents to the action potential (1Hz pacing, baseline model settings).

2.3 Restitution characteristics

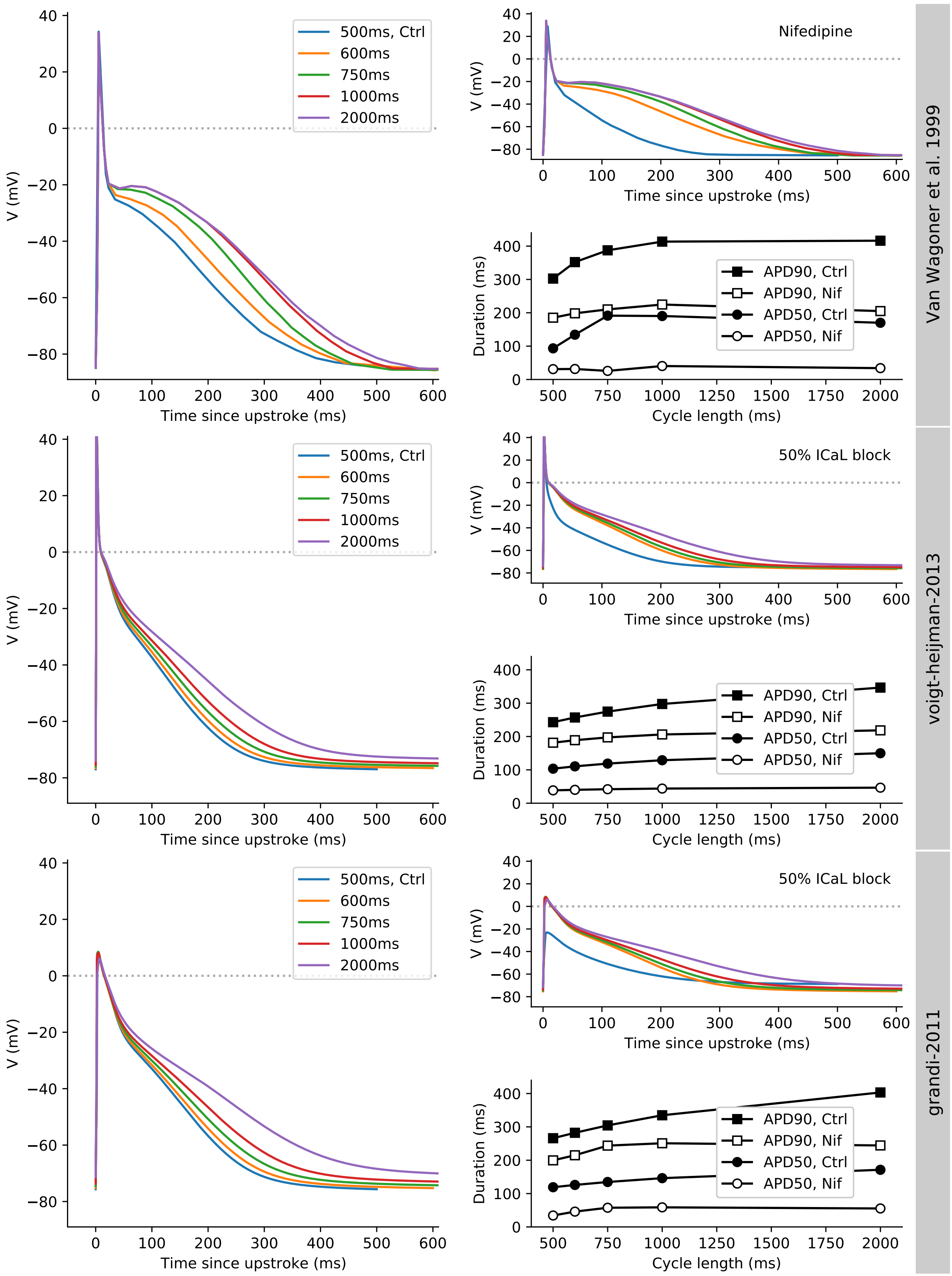

Figure S4: Restitution characteristics in the data from Van Wagoner et al. (1999) and the models by Voigt et al. (2013) and Grandi et al. (2010).

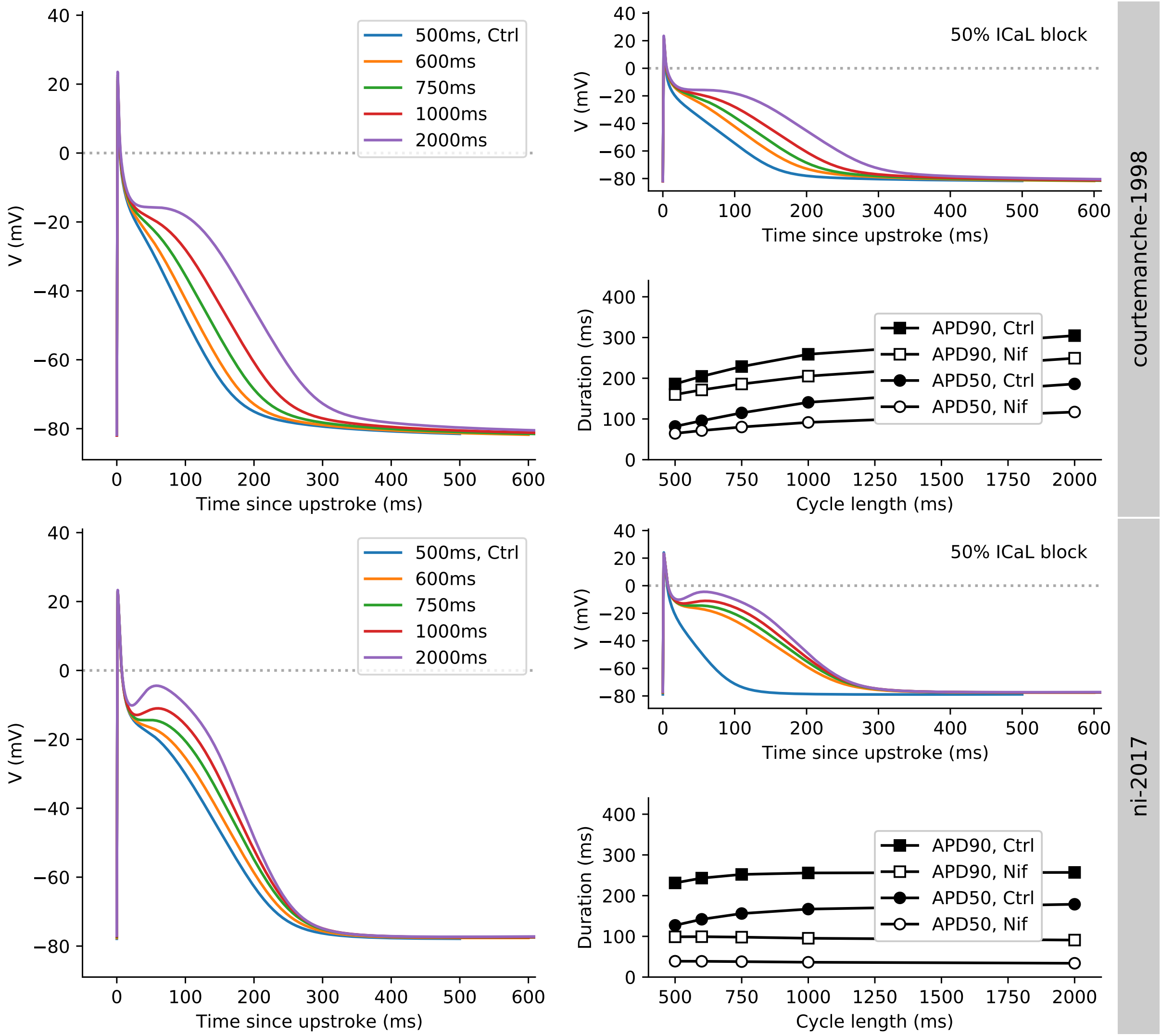

Figure S5: Restitution characteristics in the models by Courtemanche et al. (1998) and Ni et al. (2017).

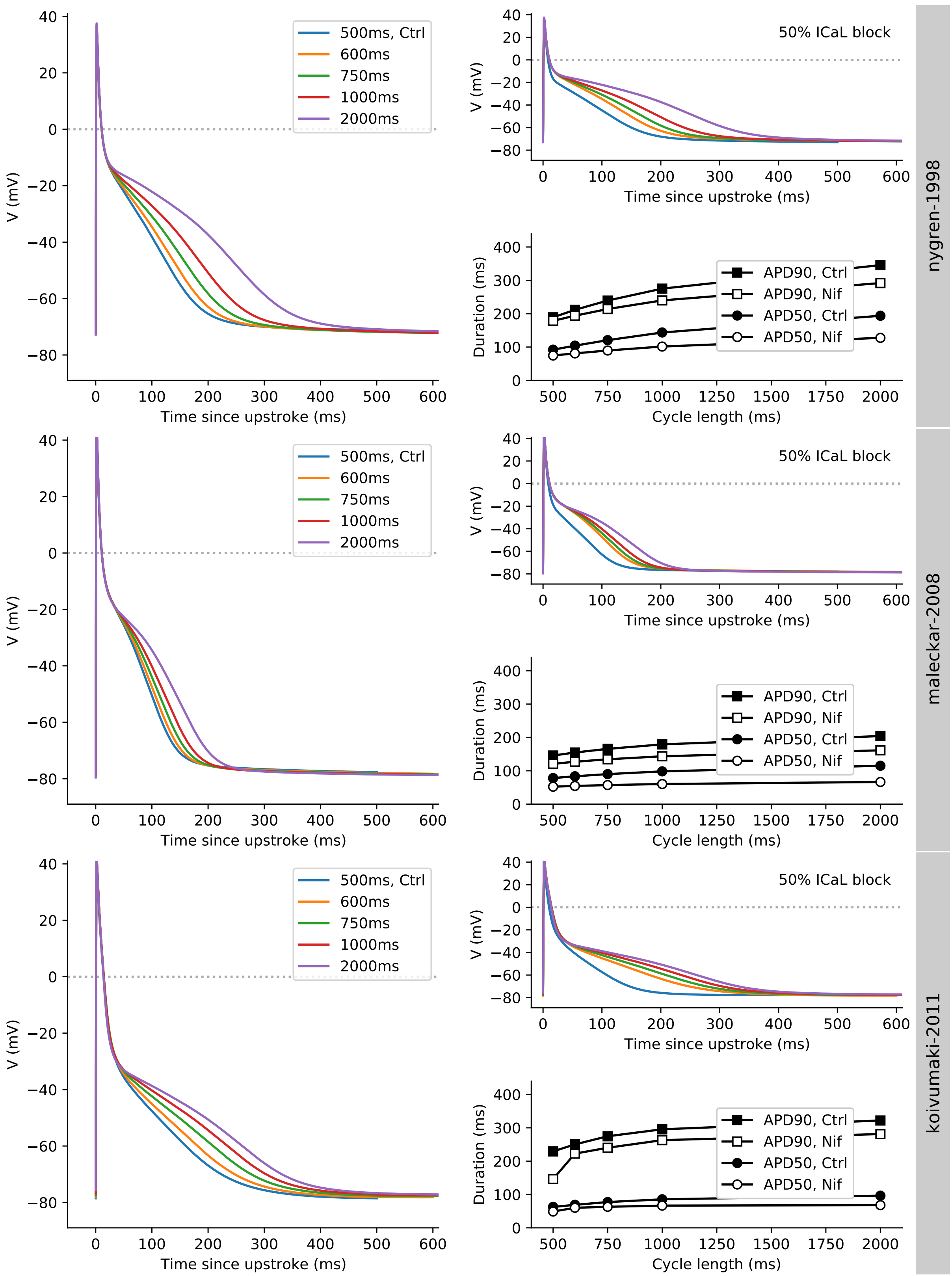

Figure S6: Restitution characteristics in the models by Nygren et al. (1998), Maleckar et al. (2008), and Koivumäki et al. (2011).

3 External potassium in equations for I_{K1} and I_{NaK}

3.1 Grandi-Pandit-Voigt and Voigt-Heijman models

The atrial model by Voigt et al. (2013) is based on the model by Grandi et al. (2011). This in turn is based on the human ventricular model by Grandi et al. (2010) which is based on a rabbit model by Shannon et al. (2004) that inherits several currents from the Guinea-pig models by Luo and Rudy (1994) and Luo and Rudy (1991). In both atrial models, the internal potassium is fixed to a constant value.

The Voigt et al. (2013) I_{K1} model is given by:

$$I_{\text{K1}} = g_{\text{K1}} \cdot x_\infty \cdot (V - E_{\text{K}}) \tag{S1}$$

$$g_{\text{K1}} = 2.1 \cdot 0.0525\sqrt{[\text{K}^+]_o/5.4} \tag{S2}$$

$$E_{\text{K}} = \frac{RT}{F}\log\frac{[\text{K}^+]_o}{[\text{K}^+]_i} \tag{S3}$$

$$x_\infty = a/(a+b) \tag{S4}$$

$$a = \frac{0.1 + 0.9/[1 + ([\text{Na}^+]_{\text{SL}}/7)^2]}{1 + \exp[0.2385(V - E_{\text{K}} - 59.215)]} \tag{S5}$$

$$b = \frac{0.49124\exp[0.08032(V - E_{\text{K}} + 5.476)] + \exp[0.06175(V - E_{\text{K}} - 594.31)]}{1 + \exp(-0.5143(V - E_{\text{K}} + 4.753))} \tag{S6}$$

where E_{K} is the reversal potential for Potassium, $[\text{K}^+]_o$ and $[\text{K}^+]_i$ are the external and internal potassium concentrations, and R, T, and F are the gas constant, temperature (in K) and Faraday constant respectively.

The variable x_∞ here represents the *rectification*. It can also be thought of as the steady-state of a very fast gating process, so that x_∞ represents the channel *kinetics*.

The I_{K1} formulation in Grandi et al. (2011) is similar in all but two equations:

$$g_{\text{K1}} = 0.0525\sqrt{[\text{K}^+]_o/5.4} \tag{S7}$$

$$a = \frac{1.02}{1 + \exp[0.2385(V - E_{\text{K}} - 59.215)]} \tag{S8}$$

The original Luo and Rudy (1991) formulation uses a as in Grandi et al. (2011), but with a slightly different scaling on the conductance term:

$$g_{\text{K1}} = 0.6047\sqrt{[\text{K}^+]_o/5.4} \tag{S9}$$

Note how there are three sources of $[\text{K}^+]_o$ dependence in these equations:

- In the driving term $(V - E_{\text{K}})$
- In the maximum conductance g_{K1}, via a square-root scaling
- In the kinetics/rectification variable x_∞, via an apparent dependence on the reversal potential. Please see Lu (2004) for a detailed explanation (which also suggests it might be better to model it as an explicit function of $[\text{K}^+]_o$, but not $[\text{K}^+]_i$).

These three effects are illustrated and compared in Figure S7.

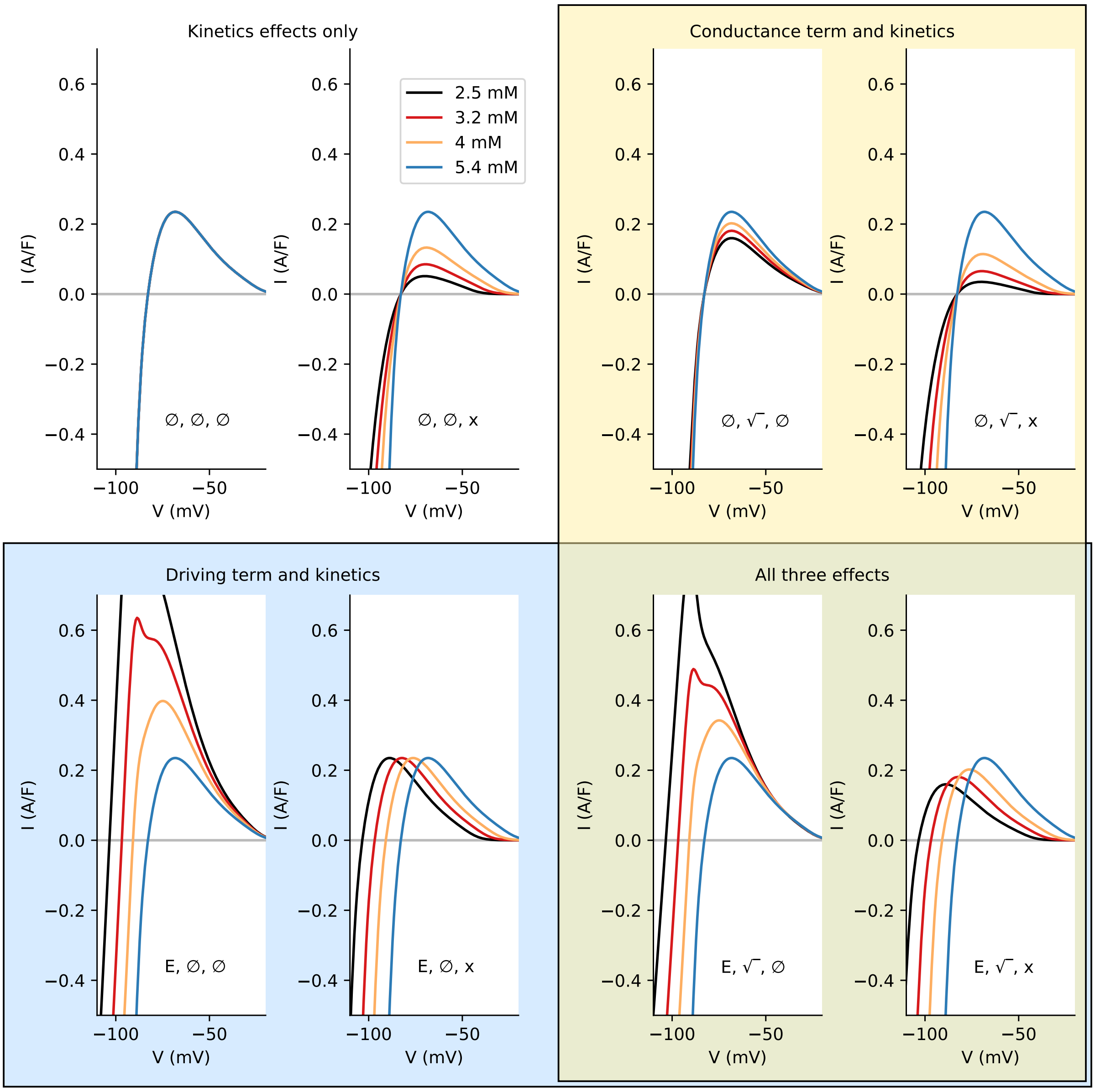

Figure S7: **$[K^+]_o$-effects on I_{K1} in the model by Voigt et al. (2013).** In this figure, the $[K^+]_o$-dependence of I_{K1} is split into three parts: *driving term* ("E") effects are only shown on the lower row; *maximum conductance* ("$\sqrt{}$") effects are only shown in the right half of the figure; and *rectification* ("x") effects are shown in the right but not the left panel within every quadrant. Comparing the top and bottom rows, we can see that the effect of $[K^+]_o$ on the driving term is profound, causing a large leftward shift of the curve, and a strong increase in current at low potentials. Comparing the left and right columns, we see that the square-root dependence on the conductance is a much more subtle effect. Comparing the left and right panels within each quadrant, we observe that the "cross-over" effect is only present if the $[K^+]_o$ dependence of the (instantaneous) kinetics is taken into account.
The second graph in the lower-left panel shows an interesting effect: because the $[K^+]_o$-dependence in the kinetics/rectification variable x_∞ is always via a term $V - E_K$, incorporating both types of $[K^+]_o$-dependence results in a simple translation of the IV-curve along the x-axis. The same effect can be seen in the Nygren model (Figure S9).

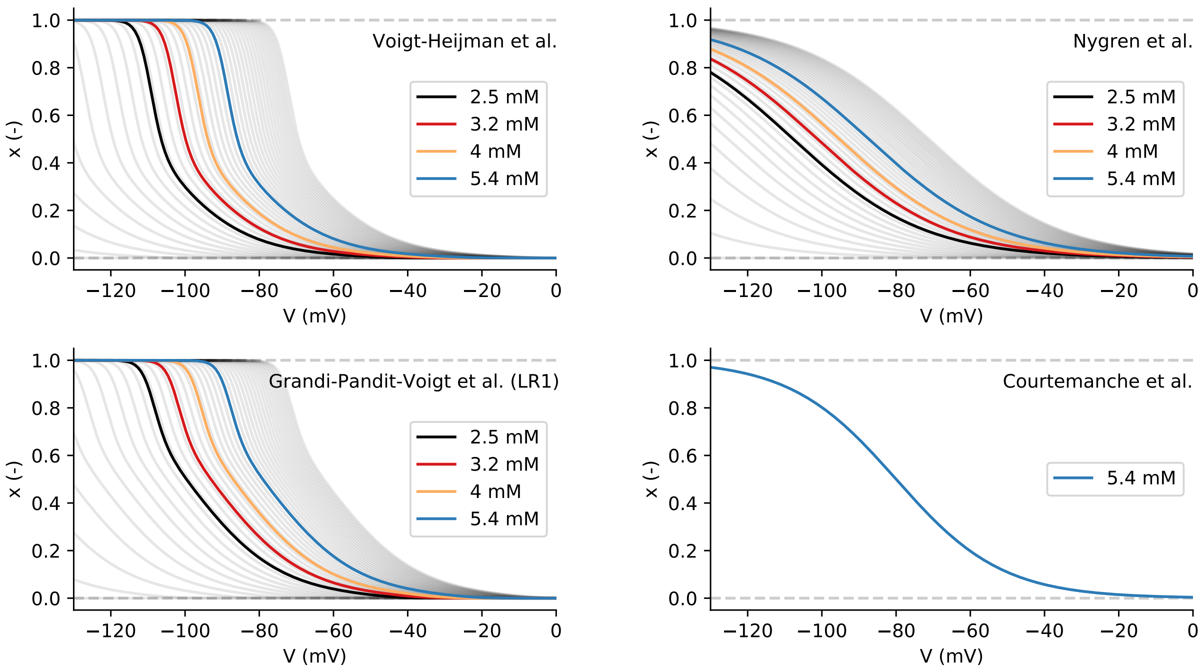

Figure S8: **$[K^+]_o$-effects on I_{K1} rectification**. In this figure, the $[K^+]_o$-dependence of the kinetics/rectification variable is shown, for the I_{K1} models by Voigt et al. (2013), Grandi et al. (2011), Nygren et al. (1998), and Courtemanche et al. (1998). The four potassium levels used in the study are shown in the usual colour-coding. The grey lines in the background show a progression from 0mM to 10mM in 40 equidistant steps.

I_{NaK} equations

I_{NaK} in the model by Voigt et al. (2013) is inherited from Grandi et al. (2011) without modification, but can be traced back to the work by Luo and Rudy (1994). The full equations are

$$\sigma = \frac{1}{7}\left[\exp\left(\frac{[Na^+]_o}{67.3}\right) - 1\right] \tag{S10}$$

$$f_{NaK} = \frac{1}{1 + 0.1245 \cdot \exp(-0.1V_f) + 0.0365 \cdot \sigma \cdot \exp(-V_f)}, \qquad V_f = \frac{VF}{RT} \tag{S11}$$

$$I_{NaK} = 1.26 \cdot f_{NaK} \cdot \frac{1}{1 + (11/[Na^+]_i)^4} \cdot \frac{[K^+]_o}{[K^+]_o + 1.5} \tag{S12}$$

where we have simplified slightly: in the full model there are two I_{NaK} currents, one into the subsarcolemmal subspace and one into the dyadic subspace. The original Luo and Rudy (1994) equations differ only in the final equation:

$$I_{NaK} = 1.5 \cdot f_{NaK} \cdot \frac{1}{1 + (10/[Na^+]_i)^{1.5}} \cdot \frac{[K^+]_o}{[K^+]_o + 1.5} \tag{S13}$$

So compared to Luo and Rudy (1994) we see a rescaling, but also a change in power from 1.5 to 4, and a slight change in the affinity for sodium.

3.2 Courtemanche and Ni models

The atrial model by Ni et al. (2017) is based on the model by Colman et al. (2013, 2017) which is based on the model by Courtemanche et al. (1998). In the code available online, the internal potassium is fixed to a constant value.

The atrial model by Courtemanche et al. (1998) is based in part on the ventricular model by Luo and Rudy (1994). Internal potassium in this model varies over time.

The I_{K1} equations in Ni et al. (2017) are identical to those in Courtemanche et al. (1998). This is a fairly simple formulation, with a single $[\mathrm{K}^+]_o$-dependence via the driving term:

$$I_{\mathrm{K1}} = g_{\mathrm{K1}} \cdot x_\infty \cdot (V - E_{\mathrm{K}}) \tag{S14}$$
$$g_{\mathrm{K1}} = 0.09 \tag{S15}$$
$$E_{\mathrm{K}} = \frac{RT}{F} \log \frac{[\mathrm{K}^+]_o}{[\mathrm{K}^+]_i} \tag{S16}$$
$$x_\infty = \frac{1}{1 + \exp(0.07(V + 80))} \tag{S17}$$

I_{NaK} equations

The $\mathrm{I}_{\mathrm{NaK}}$ formulation in Courtemanche et al. (1998) is inherited from Luo and Rudy (1994), with only a minor rescaling:

$$I_{\mathrm{NaK}} = 0.6 \cdot f_{\mathrm{NaK}} \cdot \frac{1}{1 + (10/[\mathrm{Na}^+]_i)^{1.5}} \cdot \frac{[\mathrm{K}^+]_o}{[\mathrm{K}^+]_o + 1.5} \tag{S18}$$

The Ni et al. (2017) rescales further, but also changes a power from 1.5 to 4:

$$I_{\mathrm{NaK}} = 1.28 \cdot 1.4 \cdot 0.6 \cdot f_{\mathrm{NaK}} \cdot \frac{1}{1 + (10/[\mathrm{Na}^+]_i)^4} \cdot \frac{[\mathrm{K}^+]_o}{[\mathrm{K}^+]_o + 1.5} \tag{S19}$$

3.3 Nygren, Maleckar, and Koivumäki models

The atrial model by Nygren et al. (1998) is based in part on the rabbit atrial model by Lindblad et al. (1996). In this model, internal potassium is allowed to vary over time. As described in the main text, we stabilized the model's long-term behaviour by adding the stimulus current to the list of currents affecting the internal potassium concentration, and removed the term $\Phi_{\mathrm{Na,en}}$ (see Jacquemet, 2007). This model assumes there is a 'cleft space' surrounding the myocyte, and lets the reversal potentials and the effects on I_{K1} depend on the dynamically modelled concentration on potassium in this space.

The I_{K1} formulation in this model is quite different from the equations in Lindblad et al. (1996), and is given by:

$$I_{\mathrm{K1}} = g_{\mathrm{K1}} \cdot x_\infty \cdot (V - E_{\mathrm{K}}) \tag{S20}$$
$$g_{\mathrm{K1}} = 0.03 \left([\mathrm{K}^+]_c\right)^{0.4457} \tag{S21}$$
$$E_{\mathrm{K}} = \frac{RT}{F} \log \frac{[\mathrm{K}^+]_c}{[\mathrm{K}^+]_i} \tag{S22}$$
$$x_\infty = \frac{1}{1 + \exp\left[1.5\frac{F}{RT}(V - E_K + 3.6)\right]} \tag{S23}$$

where we have converted g_{K1} to units nS/pF. Like the Grandi et al. (2011) and Voigt et al. (2013) models, this model displays three sources of external potassium concentration dependence of I_{K1}. A dissection

of the three influences is shown in Figure S9.

The atrial model by Maleckar et al. (2008) is based on the model by Nygren et al. (1998). In this model, internal potassium is allowed to vary over time. Like Nygren et al. (1998), this model assumes there is a 'cleft space' surrounding the myocyte, and lets the reversal potentials and the effects on I_{K1} depend on the dynamically modelled concentration on potassium in this space. Its I_{K1} is adapted from Nygren et al. (1998) with only a very moderate rescaling:

$$g_{\mathrm{K1}} = 0.031 \left([\mathrm{K}^+]_c\right)^{0.4457} \tag{S24}$$

The atrial model by Koivumäki et al. (2011) is also based on the model by Nygren et al. (1998), but with considerable changes to the model geometry. In particular, it no longer has the external varying 'cleft' potassium concentration, and has several internal 'spaces' where the calcium and sodium (but not potassium) concentrations differ. In this model, internal potassium is allowed to vary over time. Its I_{K1} is adapted from Nygren et al. (1998) with a slight rescaling:

$$g_{\mathrm{K1}} = 0.034425 \left([\mathrm{K}^+]_o\right)^{0.4457} \tag{S25}$$

I_{NaK} equations

The Nygren et al. (1998) equation for $\mathrm{I}_{\mathrm{NaK}}$ is rescaled from Lindblad et al. (1996), and has a slightly different affinity for potassium. It is given by

$$I_{\mathrm{NaK}} = 0.708253 \cdot \frac{[\mathrm{K}^+]_c}{[\mathrm{K}^+]_c + 1} \cdot \frac{[\mathrm{Na}^+]_i^{1.5}}{[\mathrm{Na}^+]_i^{1.5} + 11^{1.5}} \cdot \frac{V + 150}{V + 200} \tag{S26}$$

Similarly, I_{NaK} in Maleckar et al. (2008) is only slightly rescaled

$$I_{\mathrm{NaK}} = 0.6855 \cdot \frac{[\mathrm{K}^+]_c}{[\mathrm{K}^+]_c + 1} \cdot \frac{[\mathrm{Na}^+]_i^{1.5}}{[\mathrm{Na}^+]_i^{1.5} + 11^{1.5}} \cdot \frac{V + 150}{V + 200} \tag{S27}$$

The I_{NaK} formulation is re-used from Nygren et al. (1998) without modification.

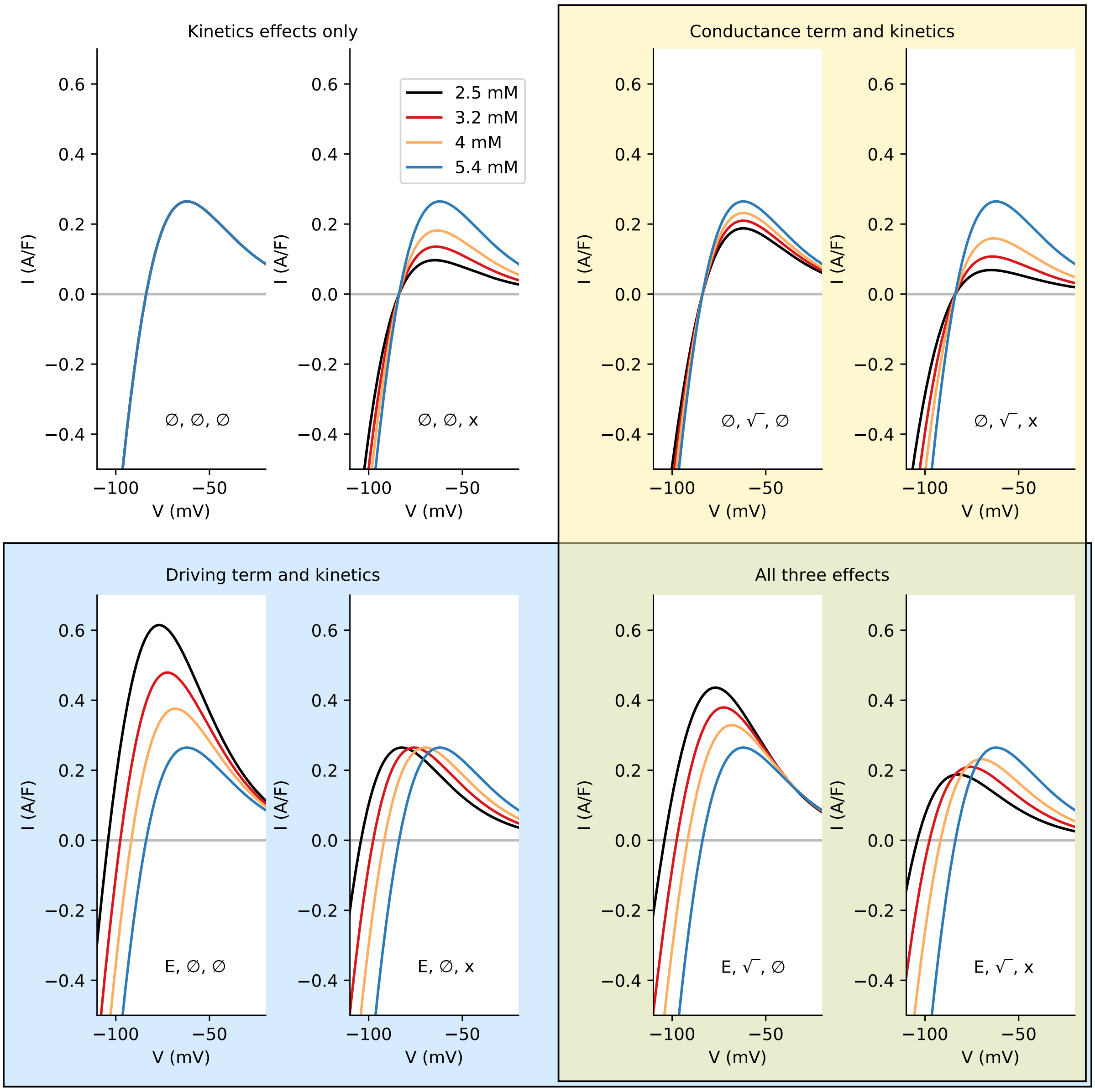

Figure S9: **$[K^+]_o$-effects on I_{K1} in the model by Nygren et al. (1998).** In this figure, the $[K^+]_o$-dependence of I_{K1} is split into three parts. The figure is similar to the one previously shown for the model by Voigt et al. (2013).

4 V_r versus $[\mathrm{K}^+]_o$: model predictions

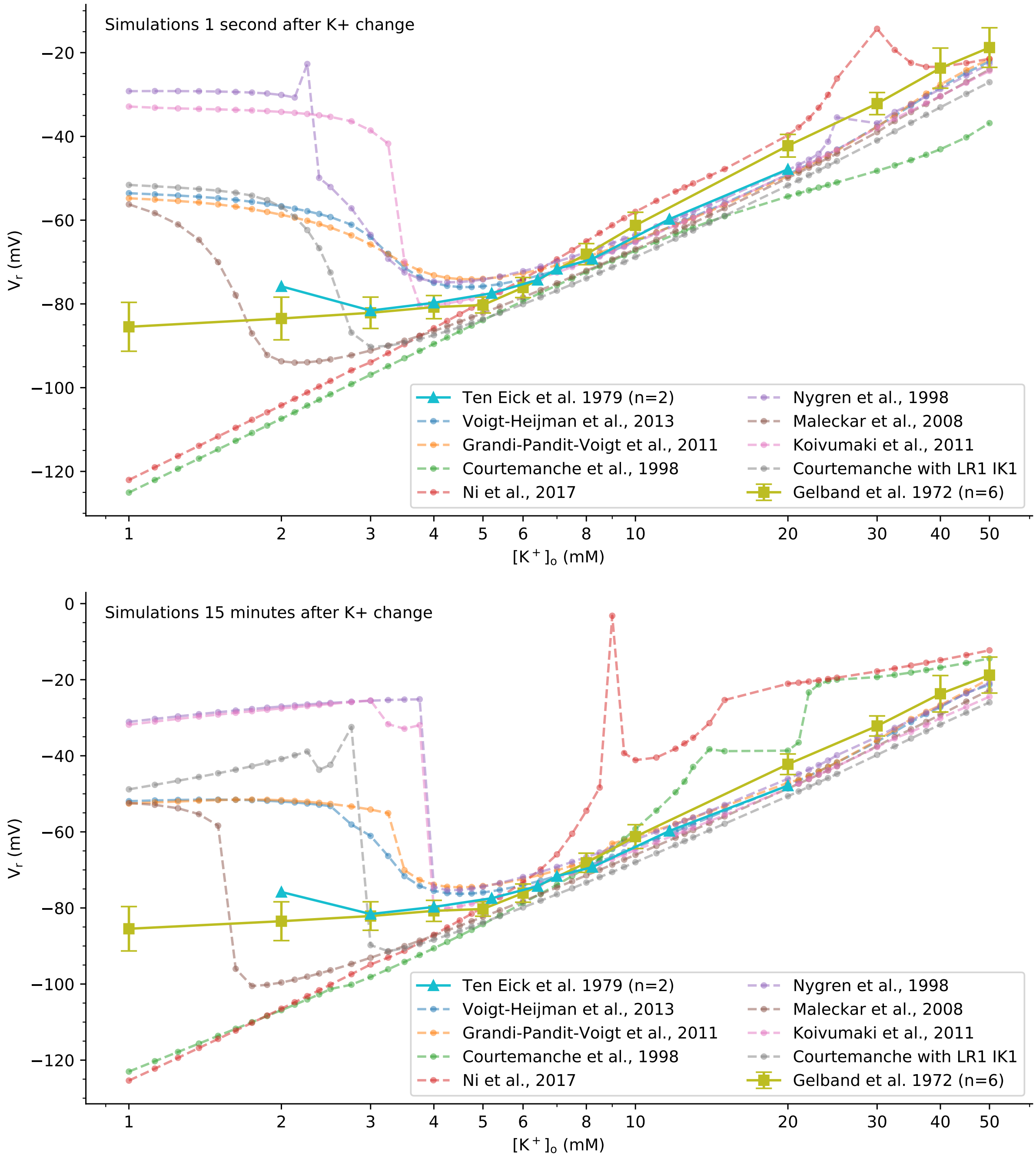

Figure S10: V_r **-** $[\mathbf{K}^+]_o$ **relationships in experiments compared with model predictions**. Predicted V_r-$[\mathrm{K}^+]_o$ relations, either 1 second or 15 minutes after a change in $[\mathrm{K}^+]_o$. All seven models are shown, plus a modified version of the Courtemanche et al. model that uses the I_{K1} formulation from Luo and Rudy (1991). Note that both experimental data sets were obtained after allowing the diastolic potential to stabilize, so that the simulations in the lower panel should match the data more closely. Interestingly, after 1 second a relatively smooth V_r-$[\mathrm{K}^+]_o$ curve is predicted, but after 15 minutes V_r has either stabilized near its "normal" value, or fully depolarized.

5 Immediate and delayed effects of $[K^+]_o$ changes

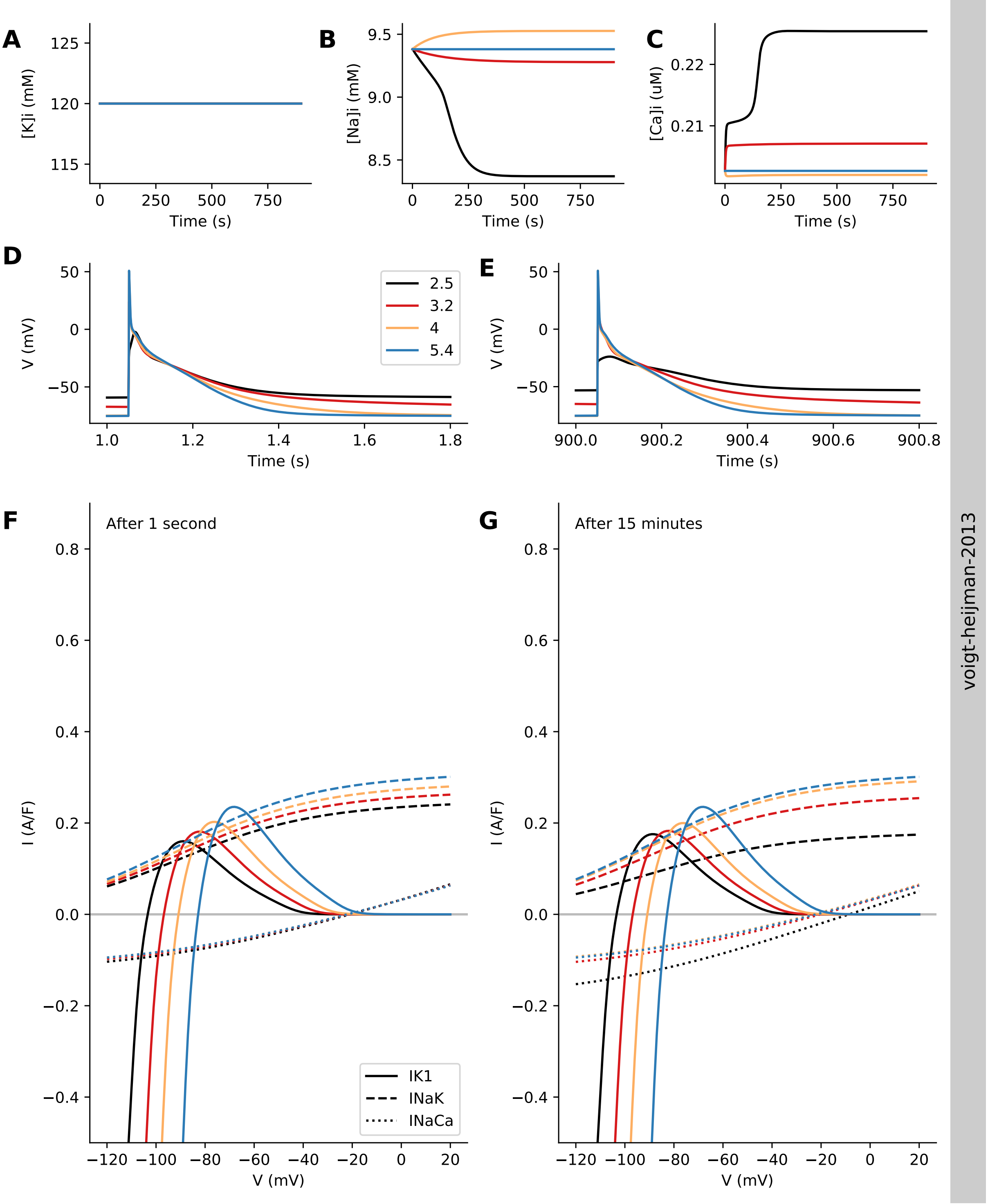

Figure S11: Immediate and delayed effects of a change in $[K^+]_o$ in the model by Voigt et al. (2013). The data in the top row was recorded once per beat, in the resting state, so transient changes are not shown.

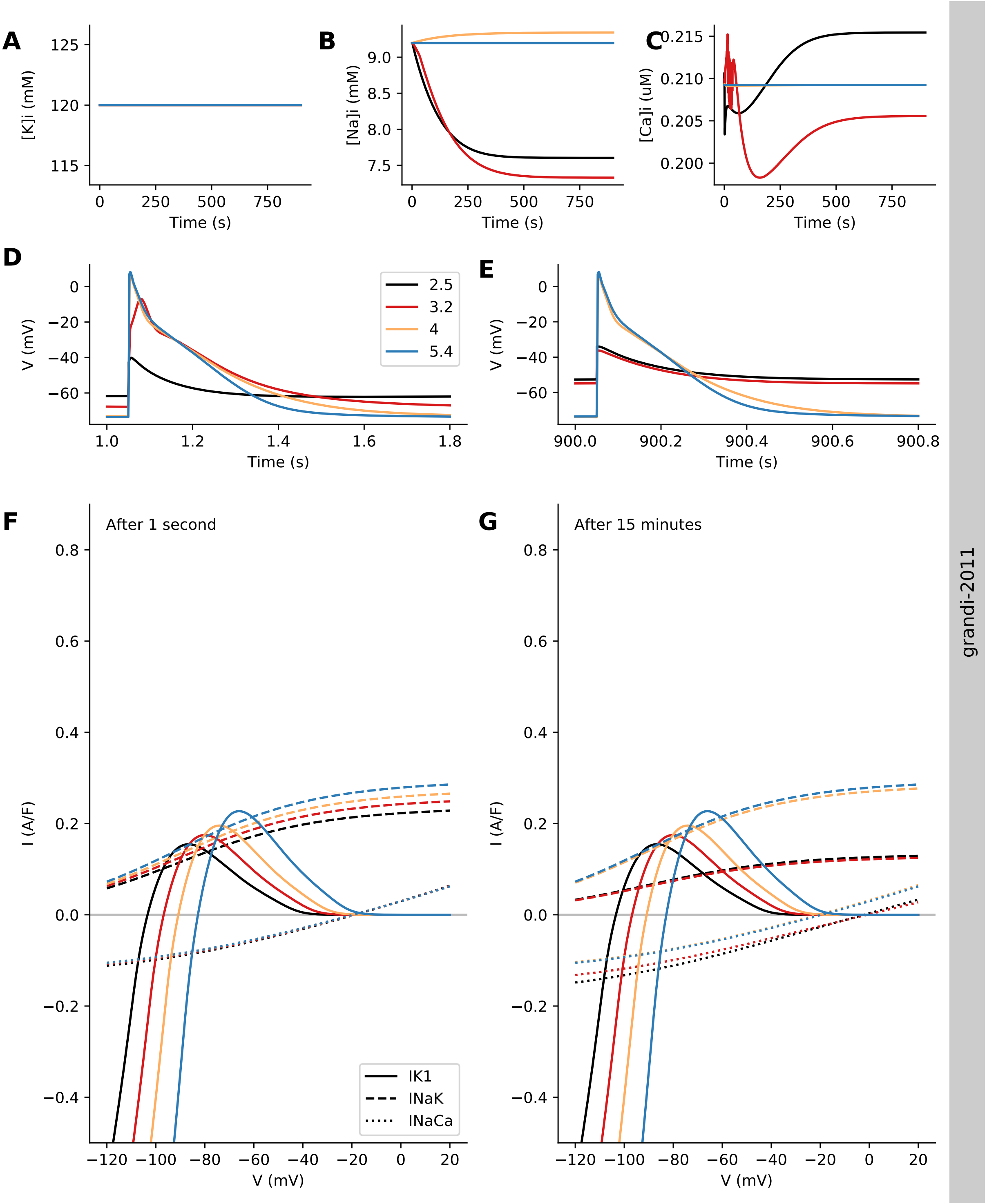

Figure S12: Immediate and delayed effects of a change in $[K^+]_o$ in the model by Grandi et al. (2011). The data in the top row was recorded once per beat, in the resting state, so transient changes are not shown.

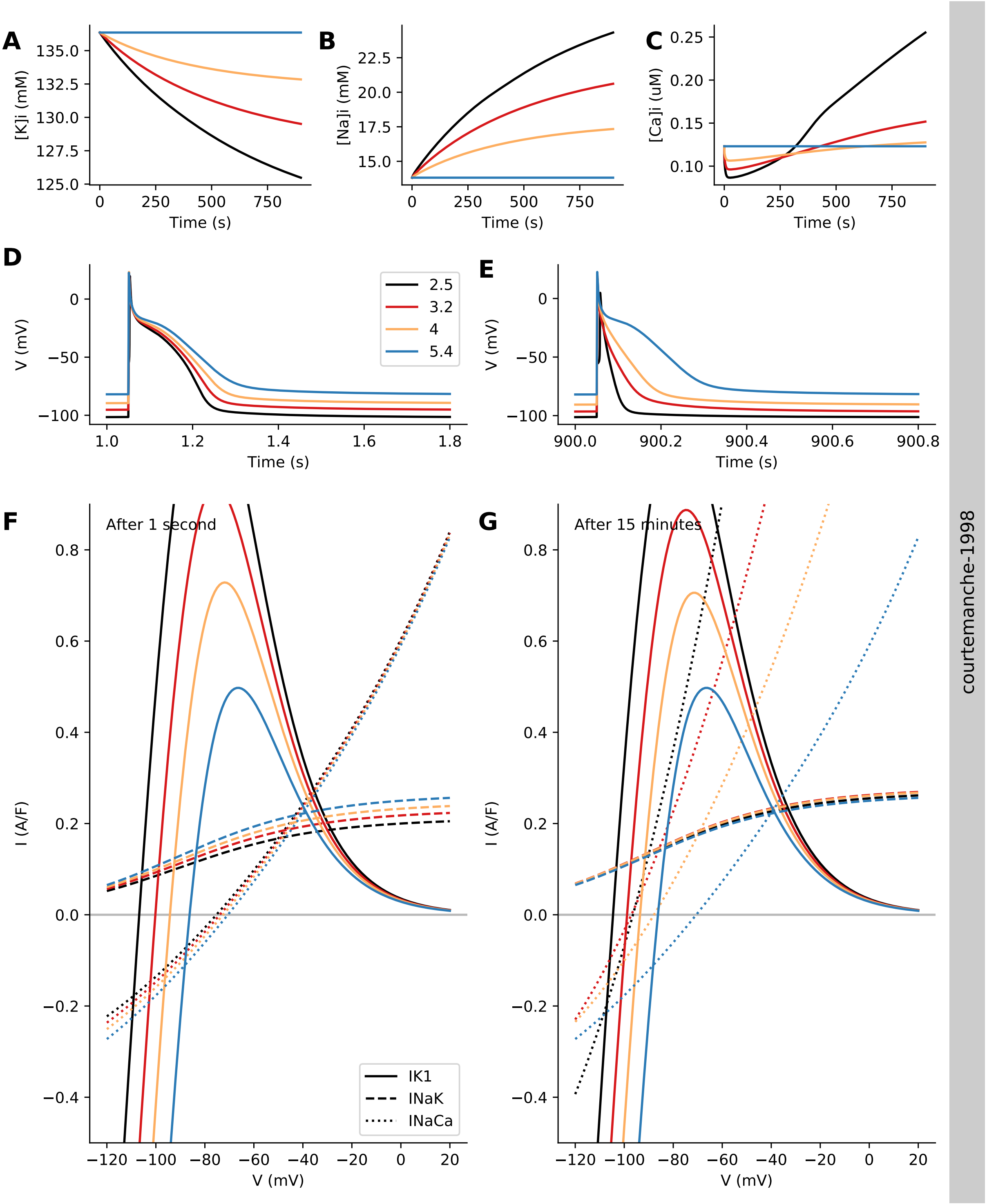

Figure S13: Immediate and delayed effects of a change in $[K^+]_o$ in the model by Courtemanche et al. (1998). The data in the top row was recorded once per beat, in the resting state, so transient changes are not shown.

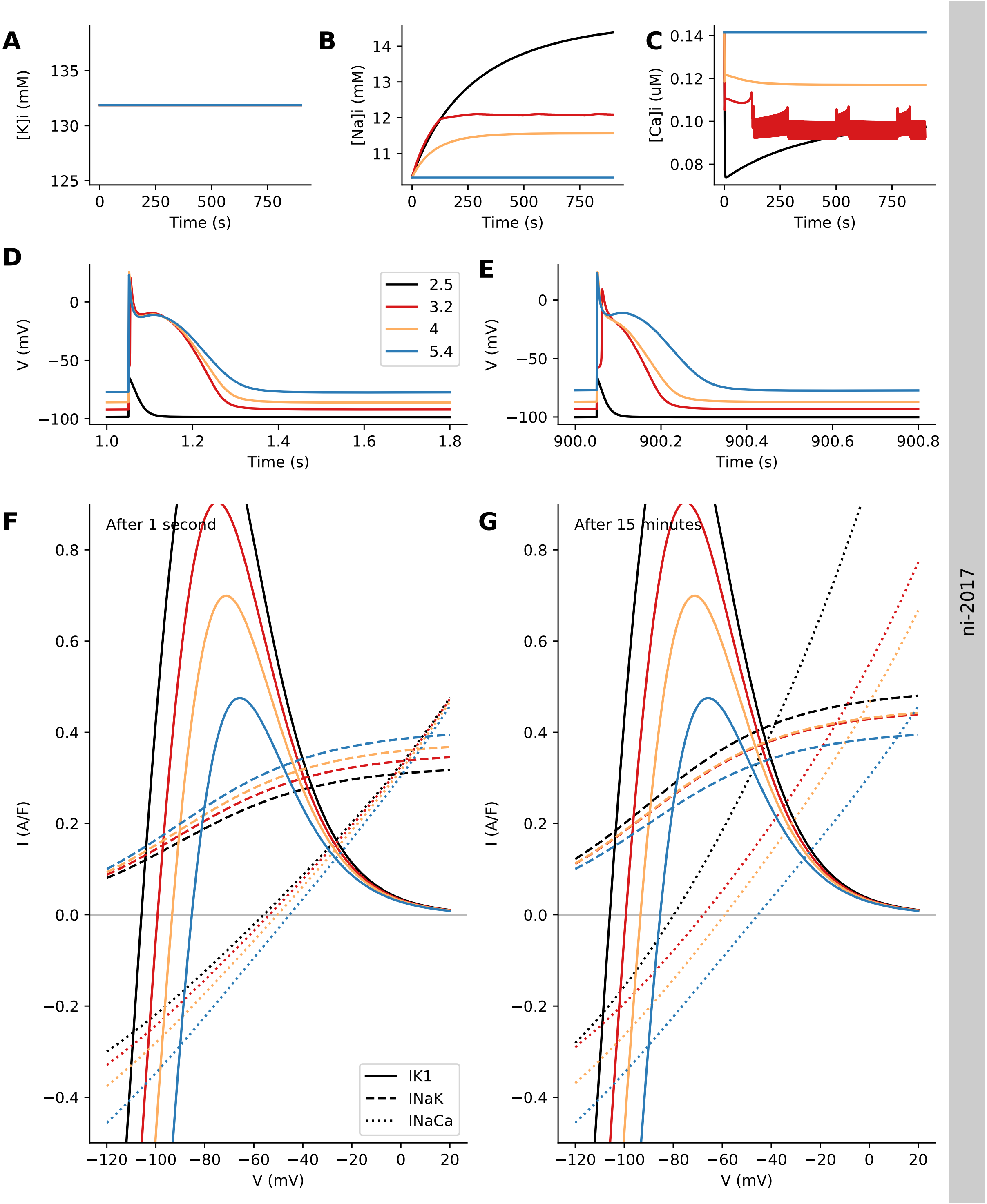

Figure S14: Immediate and delayed effects of a change in $[K^+]_o$ in the model by Ni et al. (2017). The data in the top row was recorded once per beat, in the resting state, so transient changes are not shown.

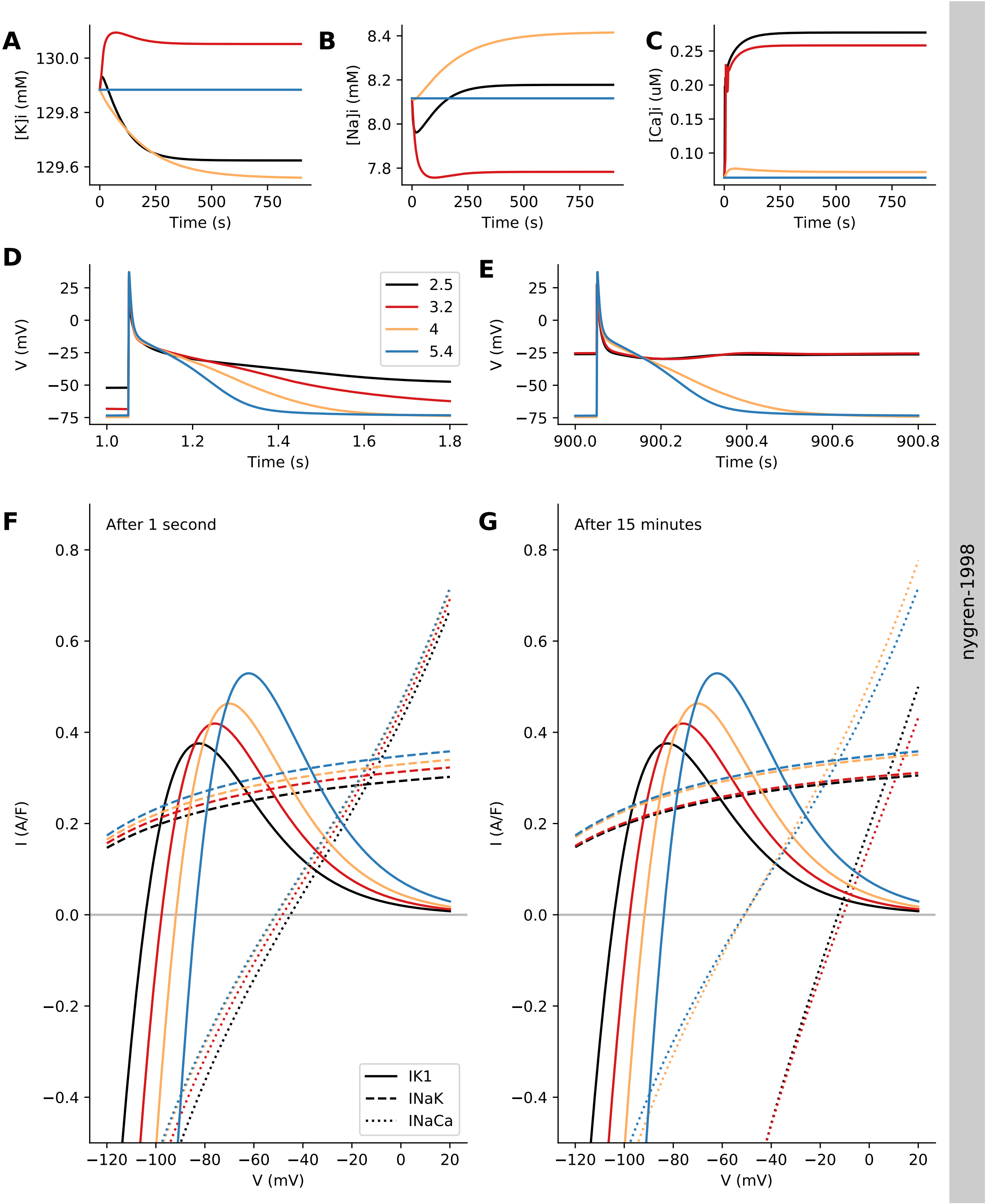

Figure S15: Immediate and delayed effects of a change in $[K^+]_o$ in the model by Nygren et al. (1998). The data in the top row was recorded once per beat, in the resting state, so transient changes are not shown.

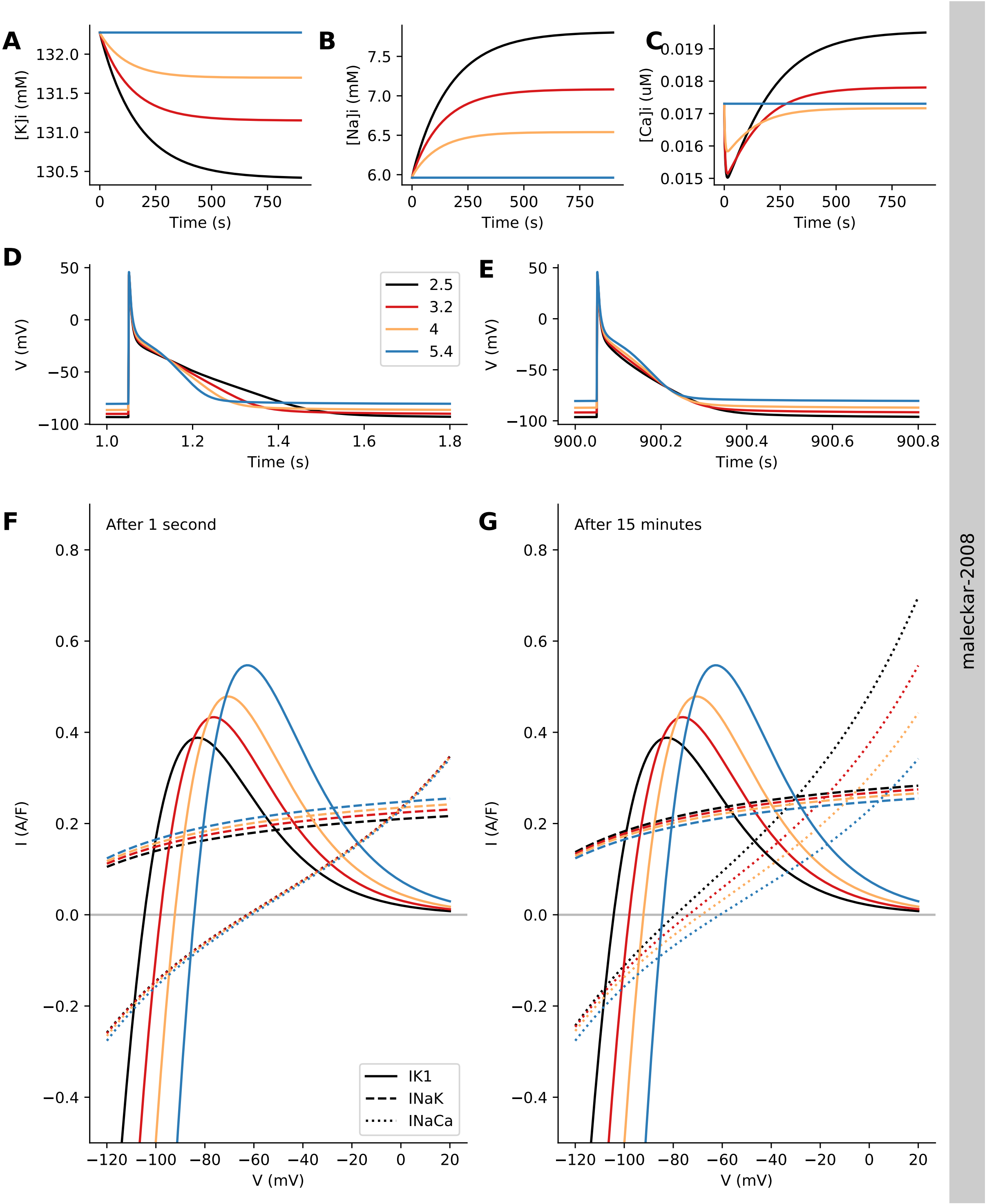

Figure S16: Immediate and delayed effects of a change in $[K^+]_o$ in the model by Maleckar et al. (2008). The data in the top row was recorded once per beat, in the resting state, so transient changes are not shown.

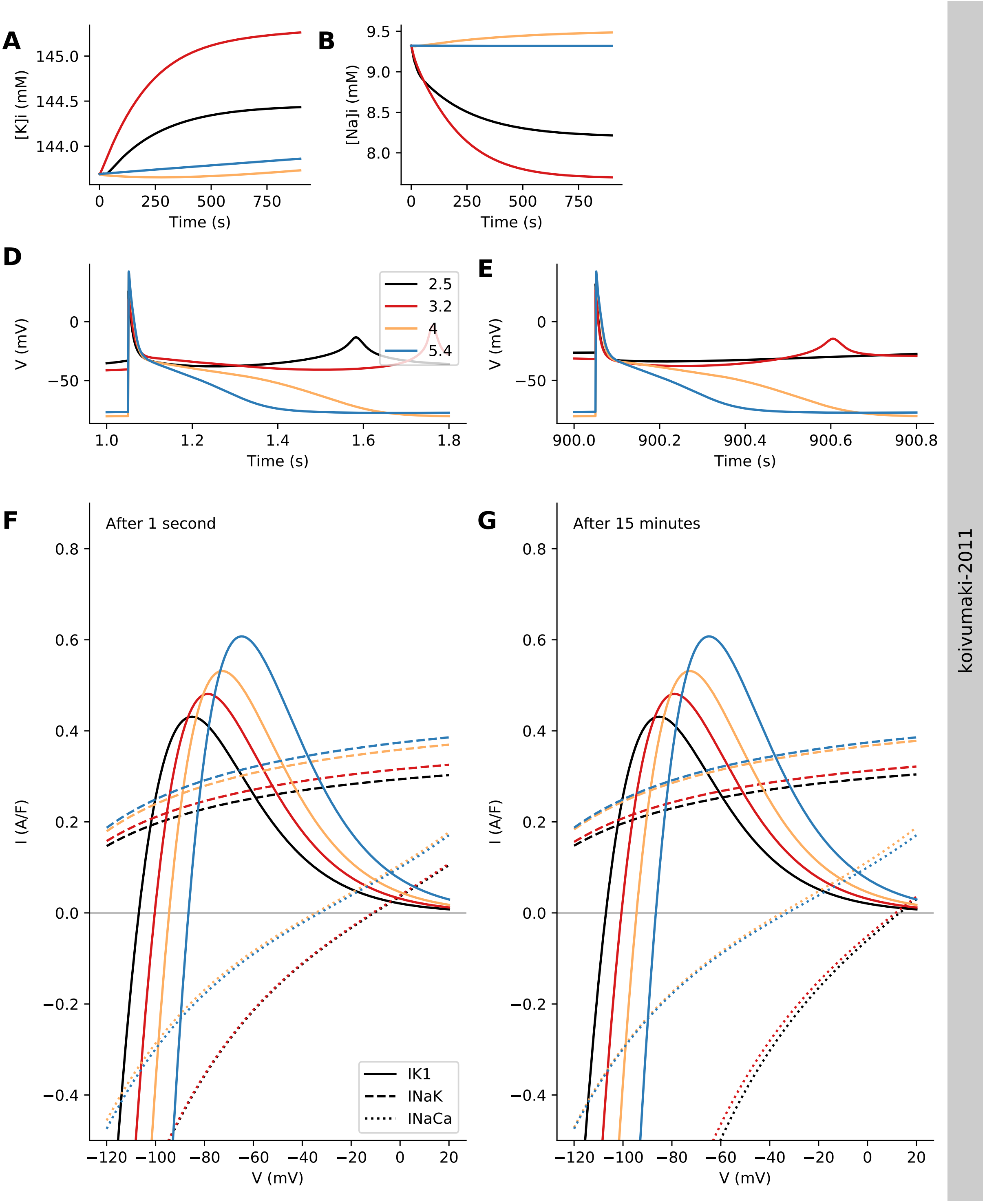

Figure S17: Immediate and delayed effects of a change in $[K^+]_o$ in the model by Koivumäki et al. (2011). The data in the top row was recorded once per beat, in the resting state, so transient changes are not shown. This model has a complex series of internal Calcium spaces, so no plot of 'bulk' $[Ca^{2+}]_i$ could be shown.

6 Strand simulations

As described in the main text, strand simulations were initialized using the single-cell 'steady-state' and then paced for a further 50 beats to allow for any changes in steady-state due to electrotonic coupling. To asses whether a new 'steady-state' had been reached, we simulated 1 more beat and compared the state variables before and after. The table below shows the maximum relative difference $e_{\max}$ in the state variables, where $x_i(n)$ indicates the value of the i-th state variable at the end of beat n.

$$e_{\max} = \max_i |(x_i(51) - x_i(50))/x_i(50)| \cdot 100\% \tag{S28}$$

Model	$e_{\max}$	Model	$e_{\max}$
Voigt et al. (2013)	0.023%	Nygren et al. (1998)	0.087%
Grandi et al. (2010)	0.012%	Maleckar et al. (2008)	0.21%
Courtemanche et al. (1998)	0.061%	Koivumäki et al. (2011)	0.016%
Ni et al. (2017)	0.058%		

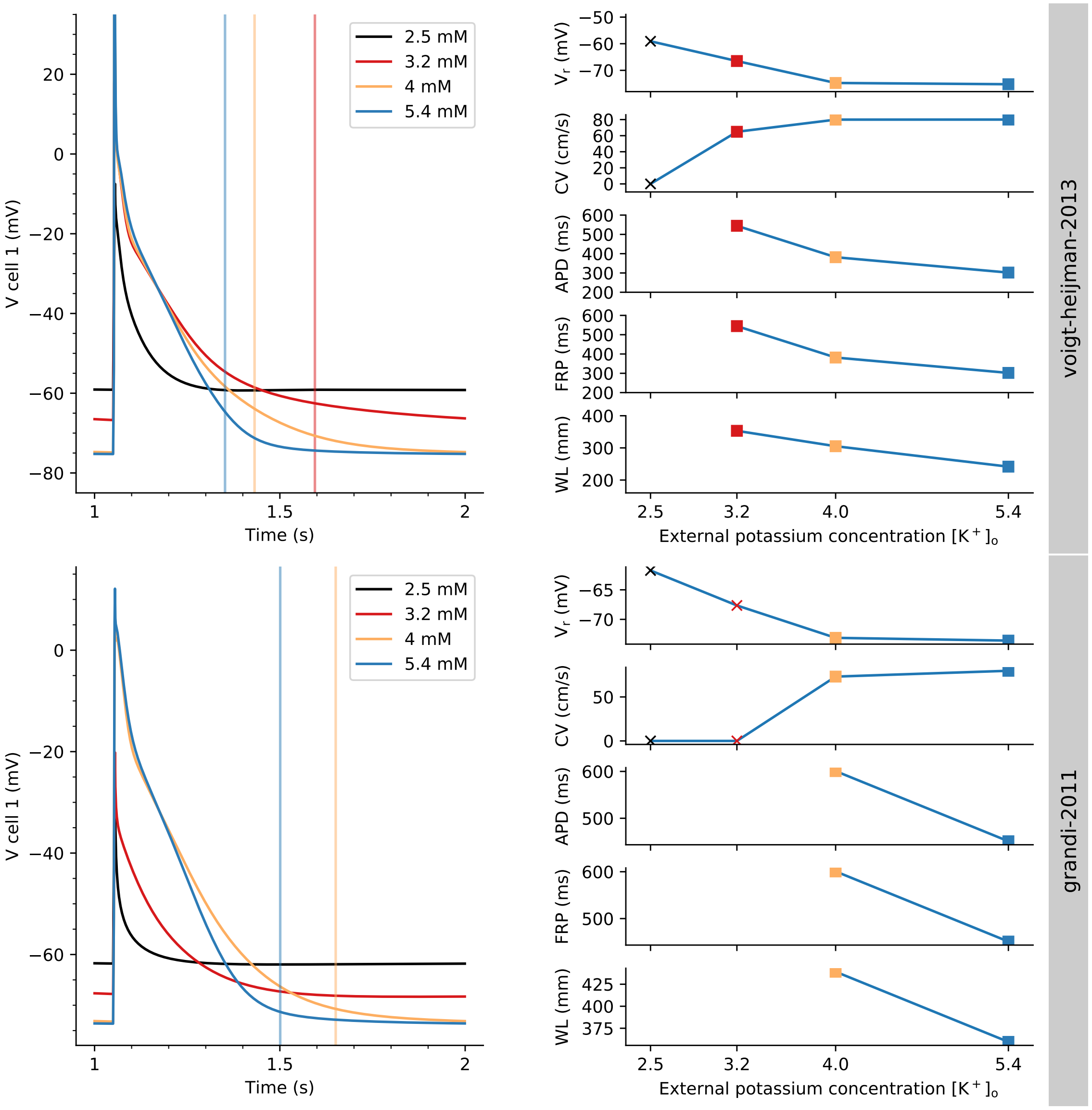

Figure S18: Strand simulation results using the models by Voigt et al. (2013) and Grandi et al. (2011). Crosses are used instead of squares where strands failed to de- or repolarize.

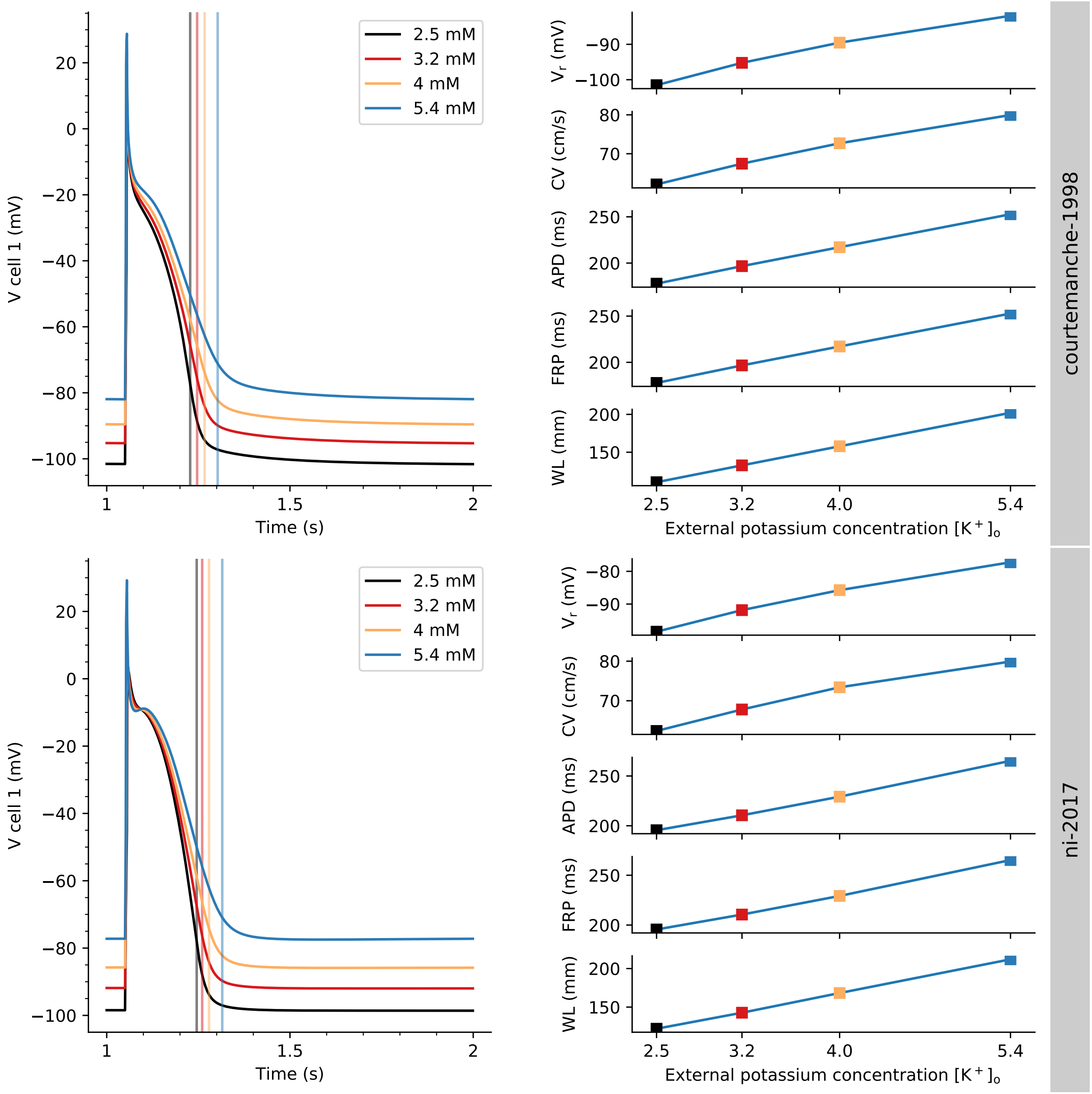

Figure S19: Strand simulation results using the models by Courtemanche et al. (1998) and Ni et al. (2017).

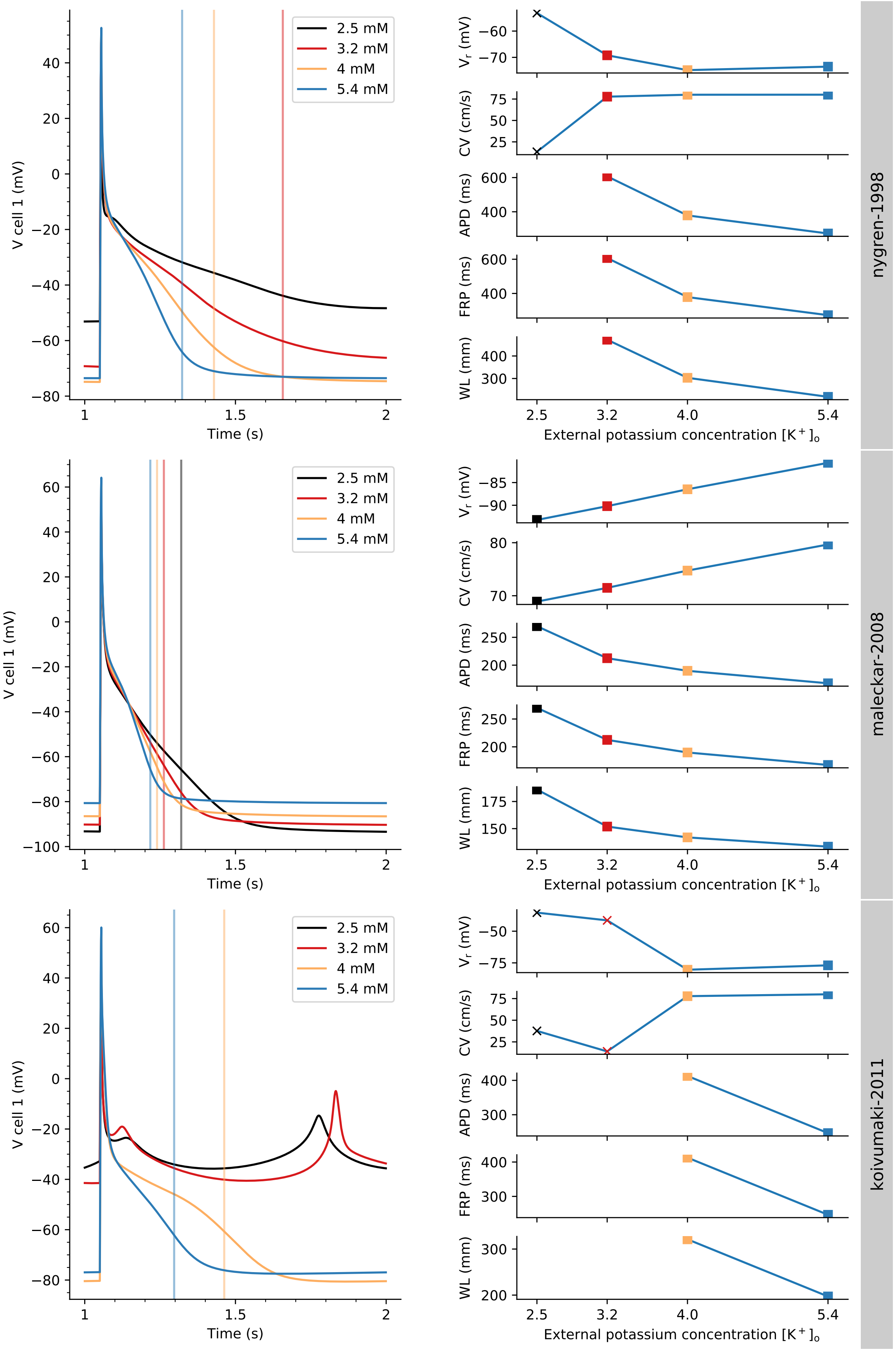

Figure S20: Strand simulation results using the models by Nygren et al. (1998), Maleckar et al. (2008). Crosses are used instead of squares where strands failed to de- or repolarize.

7 Single-cell excitability

7.1 Upstroke velocity $\dot{V}_{\mathbf{max}}$

To complement our studies on excitability and conduction in simulated strands, we investigated single-cell excitability properties. First, we show maximum upstroke velocity, which may be expected to correlate with the conduction velocity in strands.

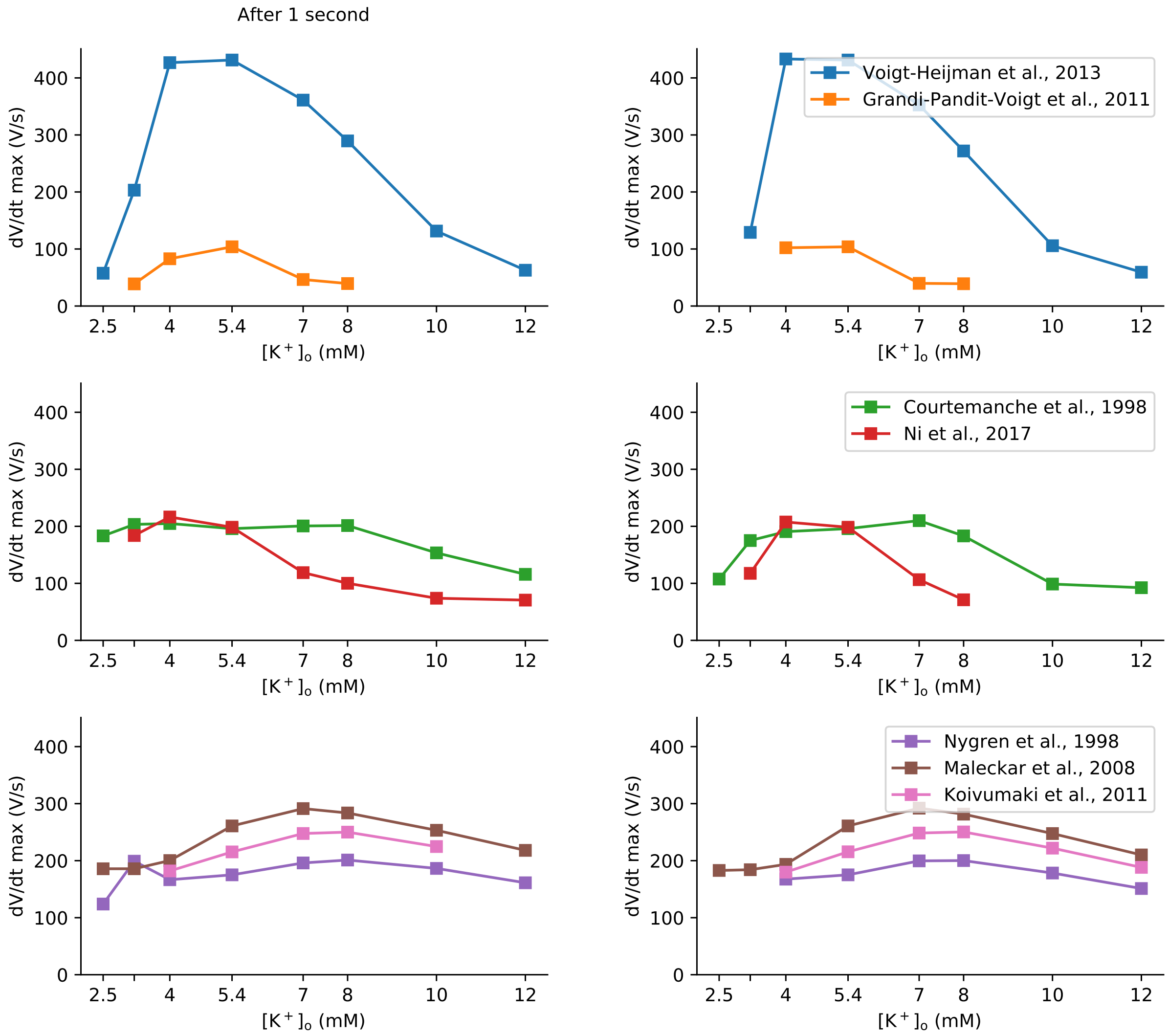

Figure S21: Maximum upstroke velocity ($\dot{V}_{\text{max}}$) versus $[K^+]_o$ in all seven models, 1 second and 15 minutes after a decrease in $[K^+]_o$. Data is only shown for concentrations at which cell models formed a 'proper' AP. Similar to the conduction velocities calculated from strand simulations, we see a general decrease in upstroke velocity under hypokalemic conditions. This decreasing trend is visible for models that depolarize at lowered $[K^+]_o$, but also for models that hyperpolarize.

7.2 I_{Na} and strength-duration curves

To further investigate the excitability of single cells, I_{Na} availability at the resting potential was plotted 1 second and 15 minutes after a change to the external potassium level. Strength-duration (SD) curves were estimated in single-myocyte simulations by first pre-pacing to a steady state at 1Hz (see above), and then using a bisection search to find the smallest stimulus amplitude that would still elicit an action potential at the tested duration.

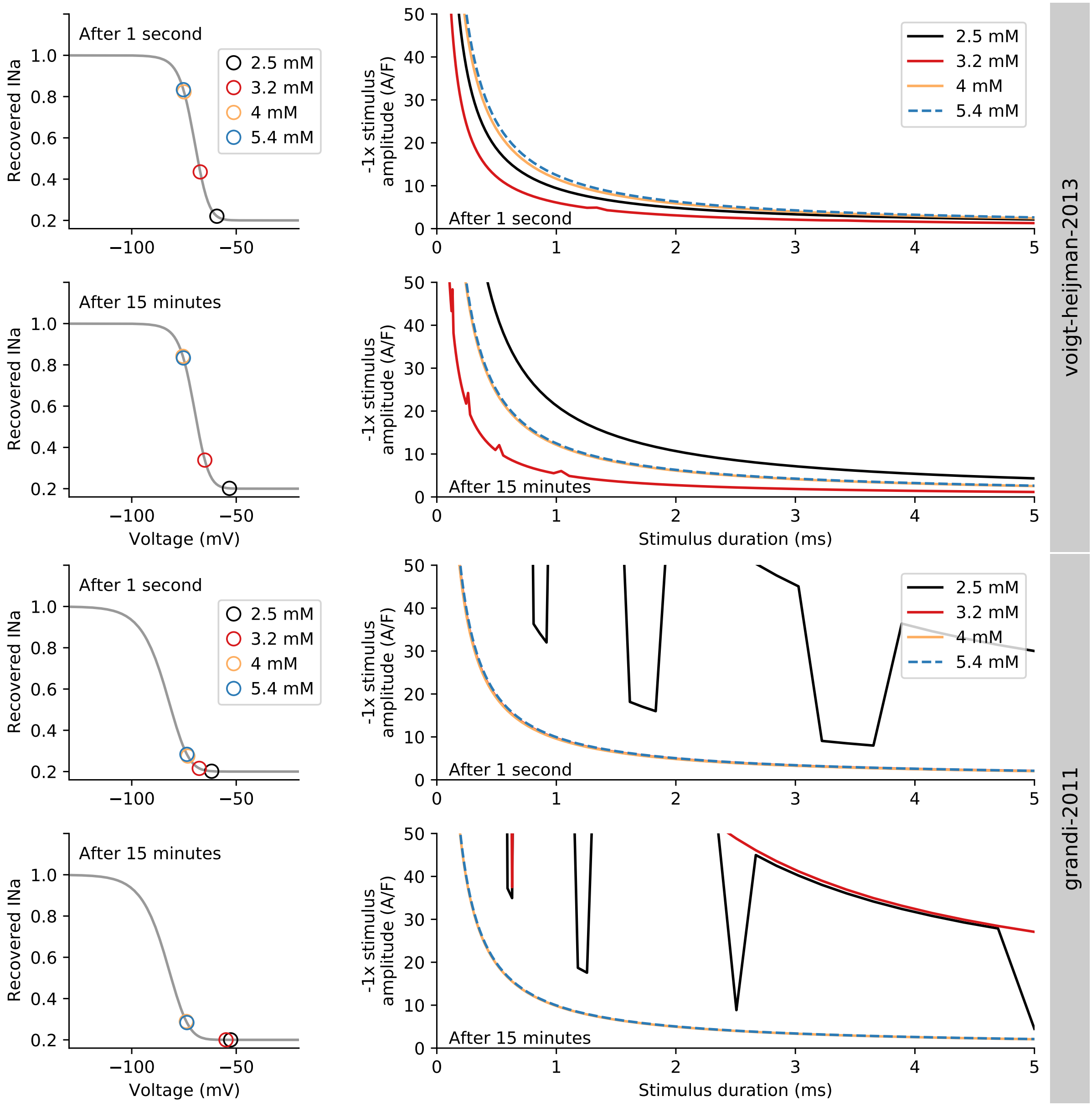

Figure S22: I_{Na} availability and SD curves for Voigt et al. (2013) and Grandi et al. (2011). Both models depolarize under hypokalemic conditions, causing a reduction in I_{Na} availability in the short-term. However, the relationship between $[\mathrm{K}^+]_o$ and excitability is not linear: the depolarisation at 3.2mM in Voigt et al. (2013) makes the model more excitable, while the effect is reversed at even lower $[\mathrm{K}^+]_o$. The Grandi et al. (2011) has very little available I_{Na} at its normal resting potential, and shows an erratic SD response for 2.5mM.

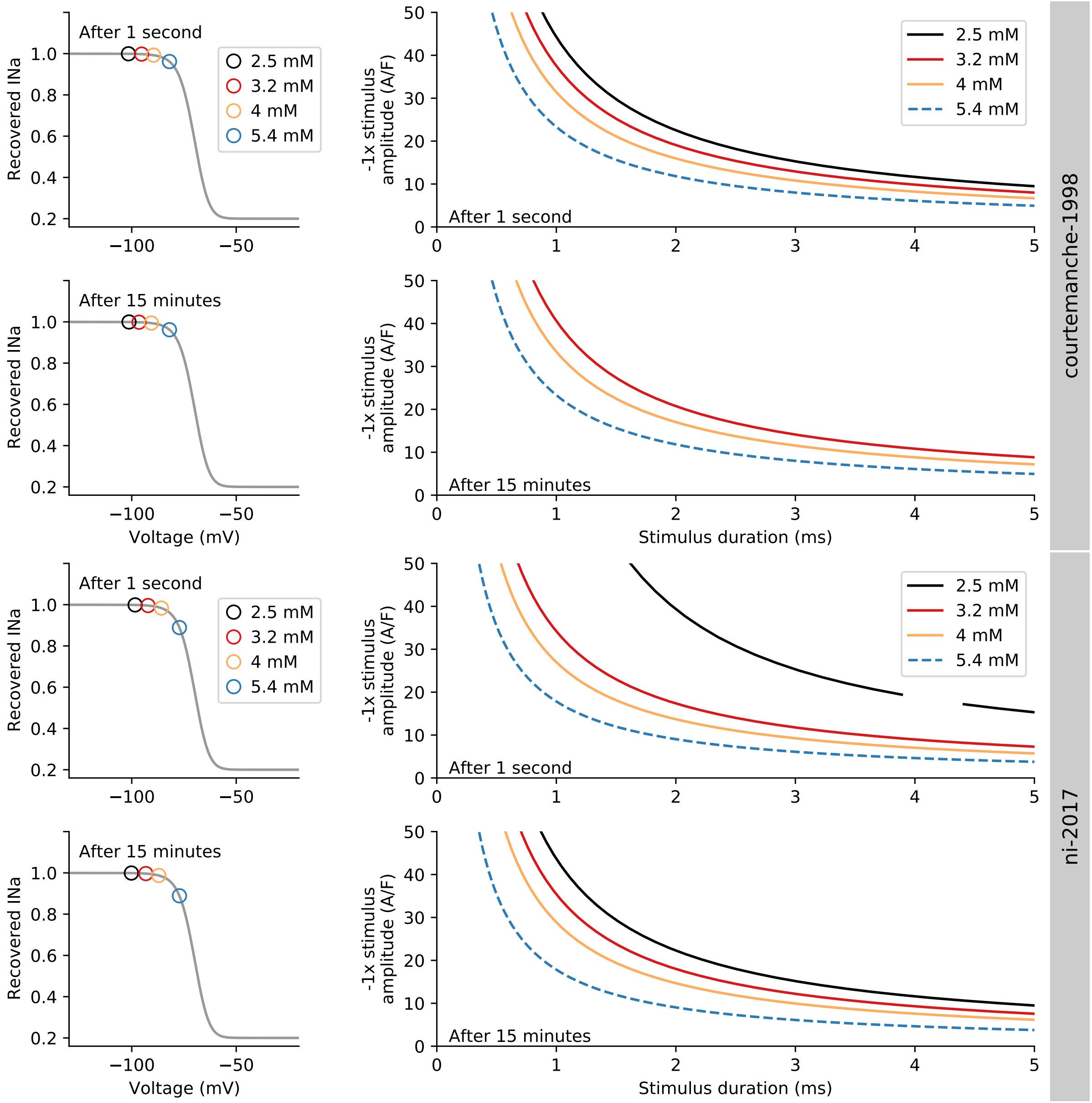

Figure S23: I_{Na} availability and SD curves for Courtemanche et al. (1998) and Ni et al. (2017). These models hyperpolarize at lowered plasma potassium levels but – in line with classical results – still require a stronger stimulus, due to the hyperpolarized V_r. In both models, this effect grows stronger after 15 minutes.

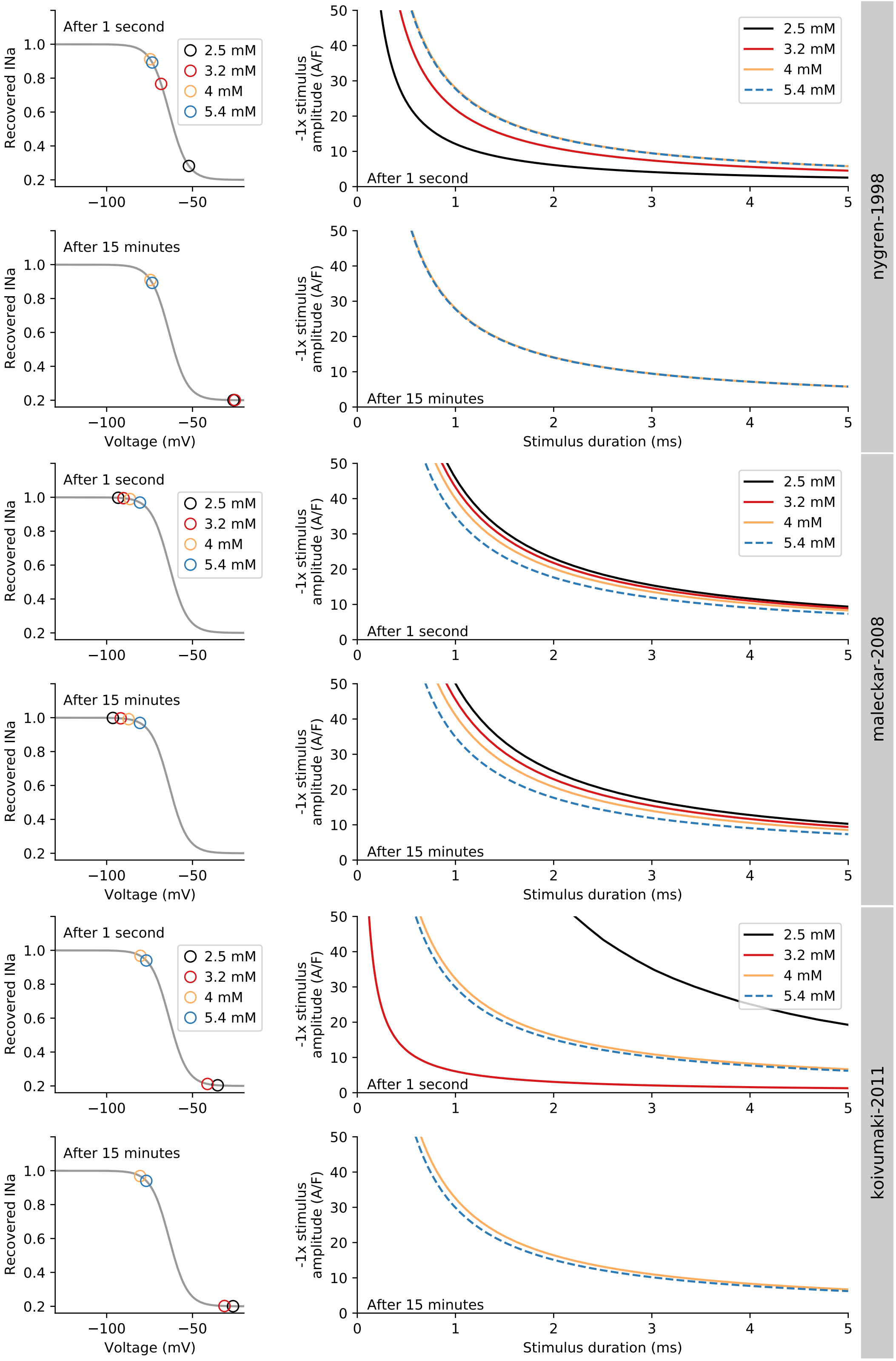

Figure S24: I_{Na} availability and SD curves for Nygren et al. (1998), Maleckar et al. (2008), and Koivumäki et al. (2011). The Nygren et al. (1998) model shows a reduced excitability shortly after the change in $[\mathrm{K}^+]_o$, but loses excitability as the membrane depolarizes further over time. The Maleckar model hyperpolarizes at the tested $[\mathrm{K}^+]_o$ levels, and so shows behaviour similar to the Courtemanche et al. (1998) and Ni et al. (2017) models. Like the Voigt et al. (2013) simulations, the Voigt et al. (2013) results clearly show a non-linear response, although excitability is lost in the longer-term simulations.

7.3 Membrane resistance estimates

Membrane resistance estimates can provide important insights about the stability of the membrane potential V_m against any perturbations (Zaniboni et al., 2000; Pouranbarani et al., 2019). A high membrane resistance implies that only small currents are needed to create a major change in membrane potential, while a low resistance implies that the V_m is more difficult to perturb. As a result, this measurement can provide information concerning the extent to which the atrial myocyte can effectively serve as a current source during the plateau phase of the AP. In addition, studying the membrane resistance at rest can provide insights into cell-to-cell conduction.

R_m values for the human atrial myocyte at selected times during the AP or the diastolic period were calculated in a similar fashion to the procedure described by Zaniboni et al. (2000). Briefly, to calculate each membrane resistance $R_m(t)$, an AP waveform was simulated up to a selected time point t, after which the transmembrane voltage, V, was clamped to preselected values: $V(t) + \Delta V$ or $V(t) - \Delta V$. Each simulation was then run for a further time period Δt. Resistance was defined as

$$R_m(t) = \frac{2\Delta V}{I(t + \Delta t, V(t) + \Delta t) - I(t + \Delta t, V(t) - \Delta t)} \tag{S29}$$

Note that, due to the time varying and non-linear nature of the underlying net ionic current, the 'resistance' estimated in this way may take on negative values (Zaniboni, 2011). Accordingly, the membrane resistance measured through this procedure is highly dependent on the chosen magnitude ΔV and duration Δt of the voltage step.

In the figures on the next two pages, we have experimented with several values, before choosing a voltage clamp perturbation of $\Delta t = 0.1$ms and $\Delta V = 10$mV. This avoids negative resistance estimates in most models under baseline conditions (5.4 mM $[K^+]_o$, and provides useful results in the model by Voigt et al. (2013) at 4.0 mM.

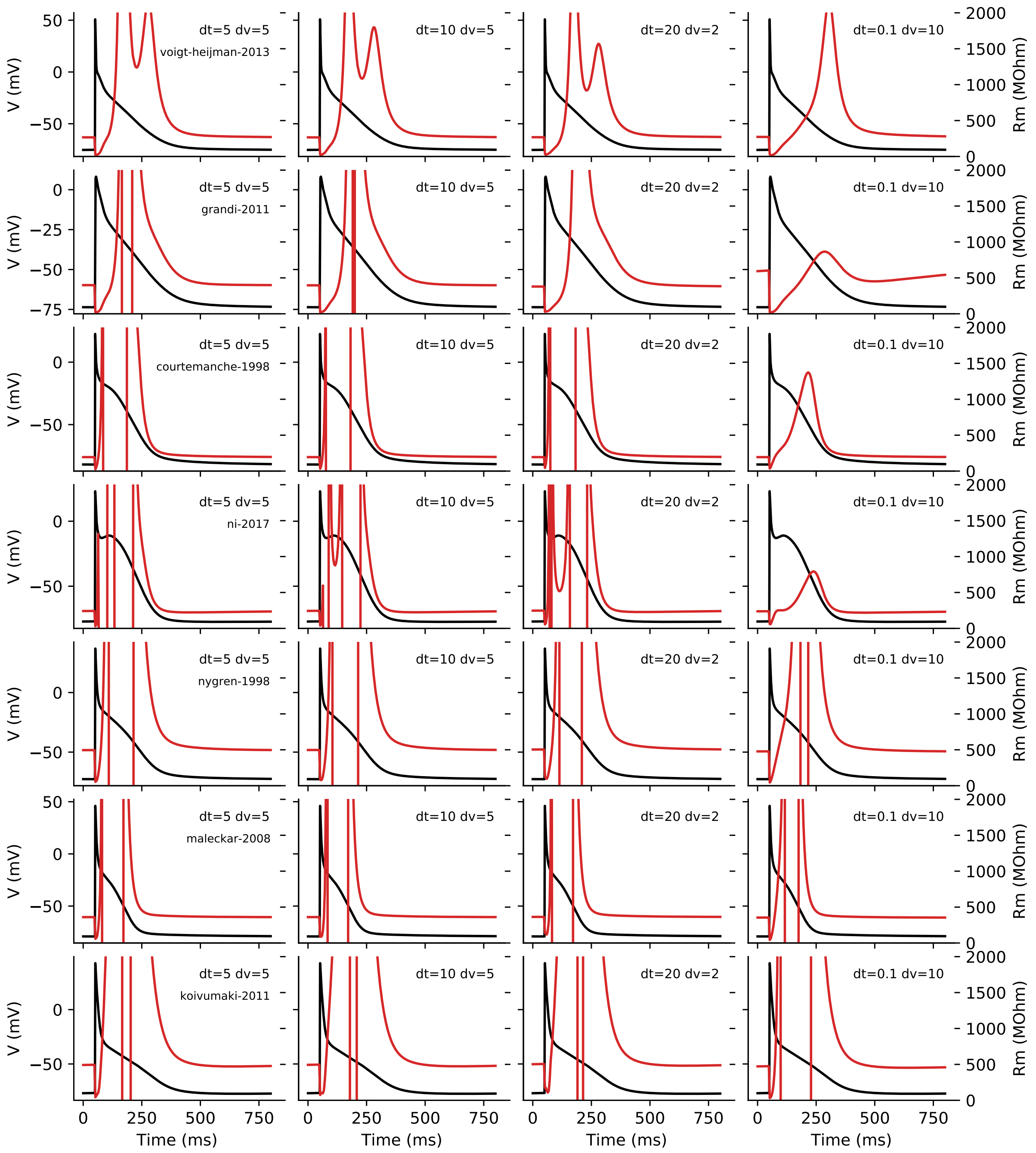

Figure S25: Membrane resistance (R_m) calculated for selected step durations Δt and magnitudes ΔV, for all models. Action potentials are shown in black, with scale information on the left-axis, while membrane resistance is shown in red, with scale information on the right-axis. Based on this figure, we selected a step of $\Delta t = 0.1$ms and $\Delta V = 10$mV, as this showed the fewest negative resistance values. (In many cases, the short time step does not allow significant current activation and feed-forward effects.)

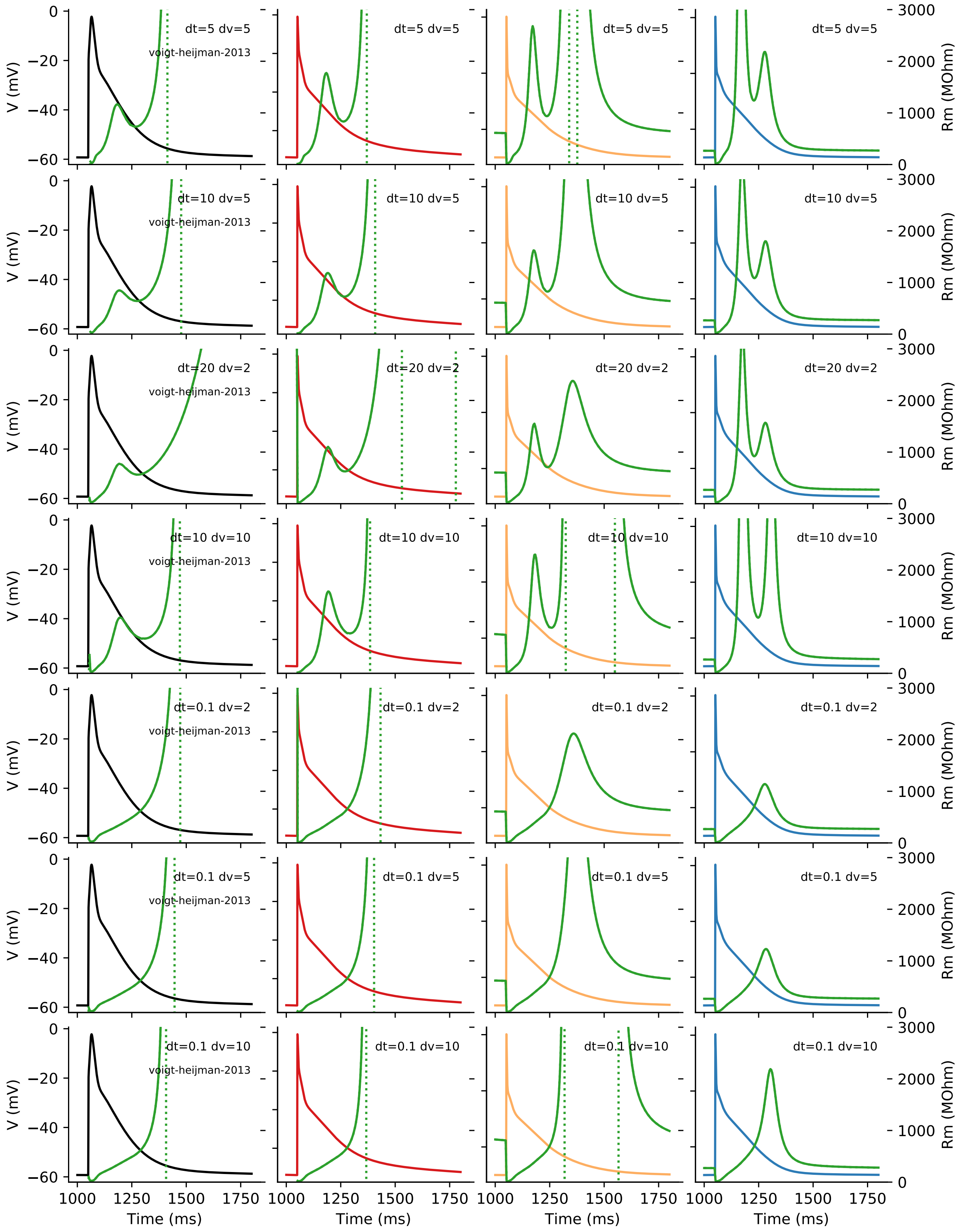

Figure S26: Membrane resistance (R_m) calculated for selected step durations Δt and magnitudes ΔV, for all external potassium levels. The first column (black APs) corresponds to 2.5mM, the second (red APs) to 3.2mM, the third (yellow APs) to 4mM, and the fourth (blue APs) to 5.4mM. R_m is shown in green throughout. This figure was less useful in choosing a step size, although again it hints at using small step sizes to prevent negative resistance estimates.

8 Single-cell stability

8.1 R_m vs. $[K^+]_o$

In this analysis we sought to determine whether in low $[K^+]_o$ settings the changes in action potential waveform, and in underlying ionic currents (particularly I_{K1}), could produce changes in R_m during the action potential that would significantly alter its stability and/or the 'source-sink' relationships (Xie et al., 2010; Weiss et al., 2017; Trenor et al., 2017) in atrial tissue in low $[K^+]_o$. Detailed investigations of relative changes in R_m during the action potential have been reported based on measurements using ventricular myocytes (Zaniboni et al., 2000; Spitzer et al., 2006). This type of analysis is less well accepted, and perhaps more difficult to interpret for determination of R_m in atrial myocytes. In these myocytes, the absence of a distinct plateau phase of the AP waveforms is a known limitation. The data below shows calculated changes in R_m during the action potential, and highlights points during the diastolic period and during the AP. The APs in Row A were obtained in each of the levels of $[K^+]_o$ that were used in this study, while the panels in Row B illustrate the corresponding R_m values. Note that changes in $[K^+]_o$ significantly alters R_m during the diastolic period in 4.0 mM $[K^+]_o$, where R_m increases in diastole, decreases at the start of the AP, but then rises.

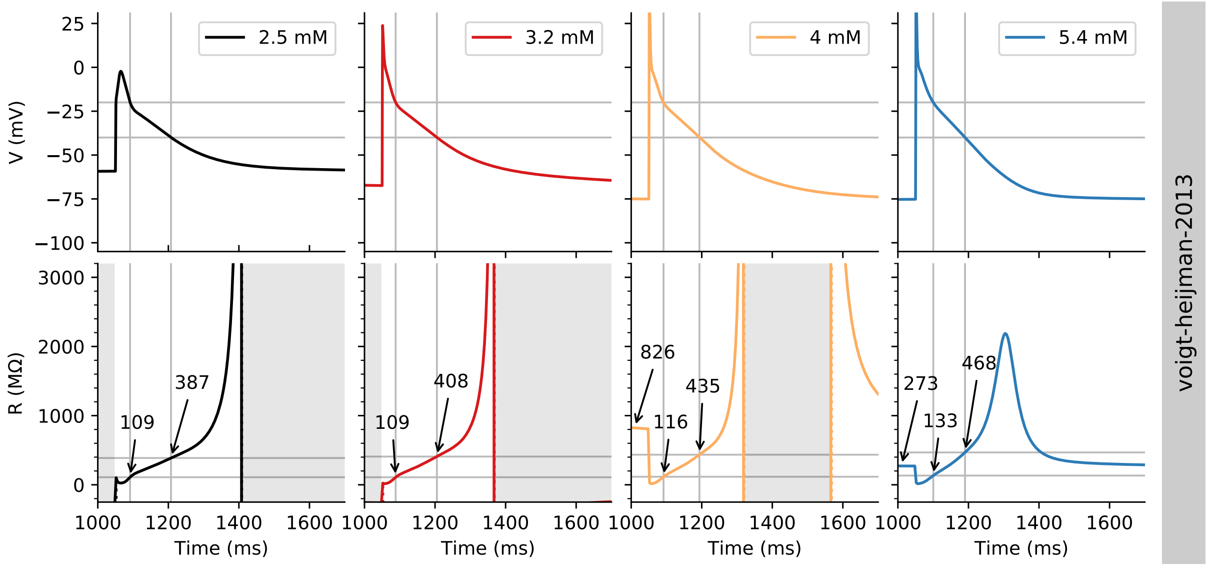

Figure S27: **Phase-dependent Changes in Membrane Resistance at Different $[K^+]_o$ Levels.** Each panel in Row A consists of one single myocyte AP simulation obtained at the $[K^+]_o$ level shown. These same AP records were superimposed in Figure 1, Row A (left) of the main manuscript. Row B shows the corresponding calculated values of R_m, with gray shading indicating the areas where a negative resistance was obtained. The numbers on each R_m trace denote calculated values i) in diastole (where possible), ii) during early repolarization and iii) at mid repolarization. Note that in each $[K^+]_o$ condition following the initial rapid depolarization of the AP there is an approximate 3-fold increase in R_m during the brief plateau and continuing well into the repolarization phase. In this model, when moving from 5.4 mM to 4mM, we observe an increased membrane resistance at V_r, indicating that small currents can cause large differences in V, and perhaps in action potential duration. Similarly, the resistance during the late phases of the AP is elevated when $[K^+]_o$ is lowered, indicating that the cell will react more strongly to small variations in current. For hypokalemic conditions, this model even predicts a negative resistance, indicating that small current can trigger feed-forward processes that amplify rather than oppose them.

Equivalent analysis were performed in the other models, where similar patterns of increased and negative R_m were seen.

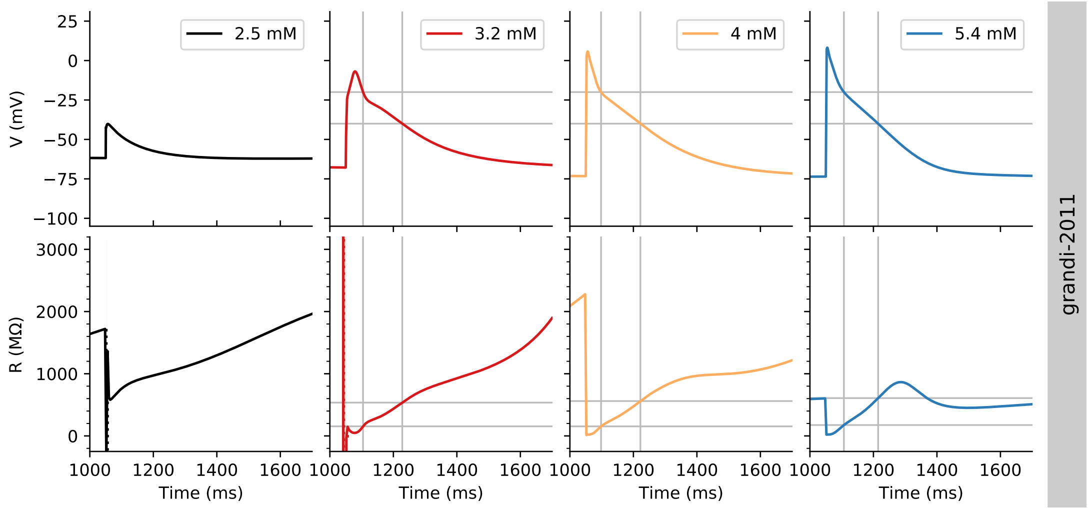

Figure S28: Action potential, I_{K1}, and membrane resistance in the model by Grandi et al. (2011).

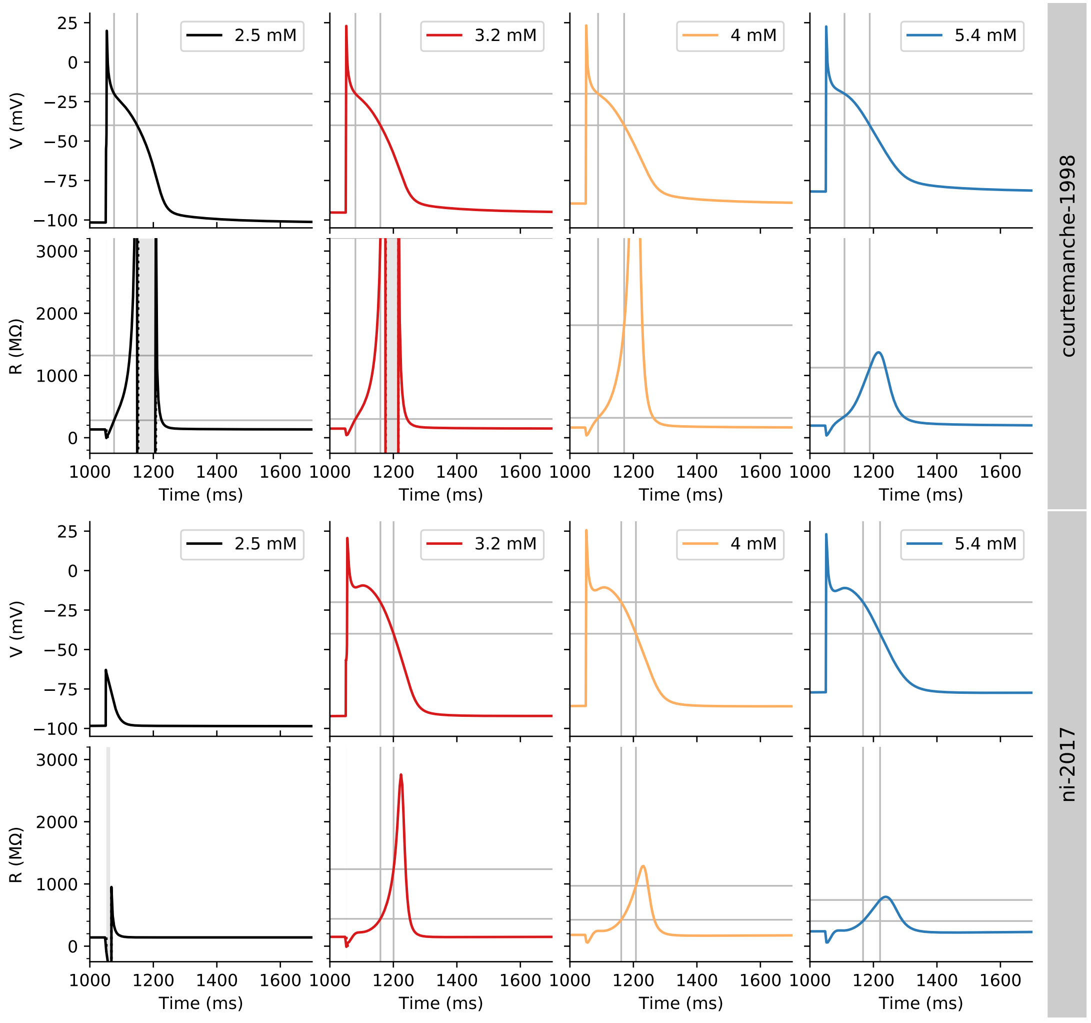

Figure S29: Action potential, I_{K1}, and membrane resistance in the models by Courtemanche et al. (1998) and Ni et al. (2017).

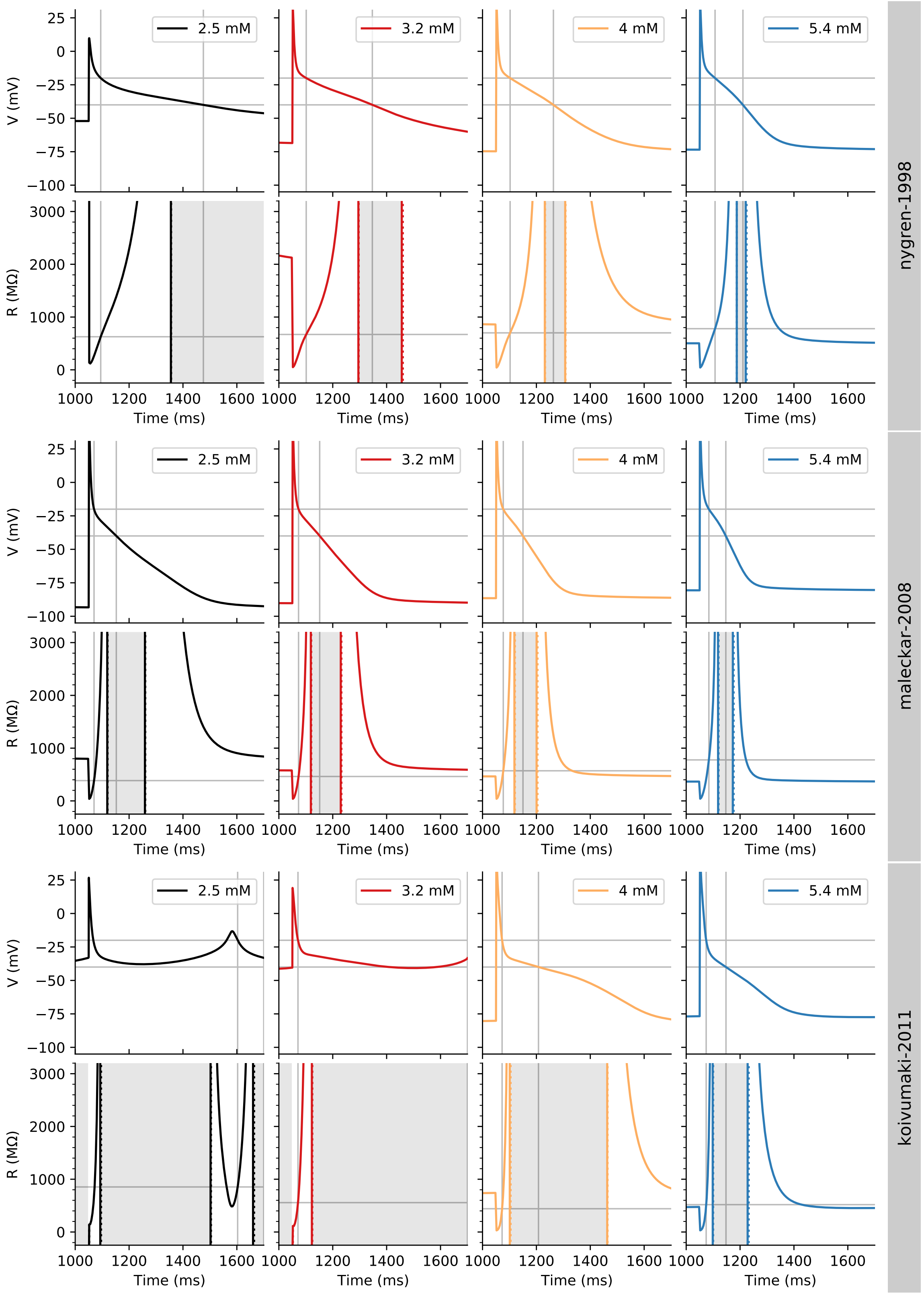

Figure S30: Action potential, I_{K1}, and membrane resistance in the models by Nygren et al. (1998), Maleckar et al. (2008), and Koivumäki et al. (2011).

8.2 Quasi-instantaneous I-V relationships

As an alternative or addition to membrane resistance estimates, we plotted 'quasi-instantaneous I-V curves' using the procedures described in Jack et al. (1975, page 250) and Fink et al. (2006). For each curve shown, an AP simulation was run up to a selected time, t; at this point, all state variables were fixed to their current values and the model's fast sodium current activation gate variable was set to its steady-state value. With these changes in place, the membrane potential was varied and the resulting current was calculated at each potential.

Similar to membrane resistance estimates, these curves provide insight into the stability of the membrane potential Zaniboni and Cacciani (see 2014): a flat slope in an instantaneous IV curve indicates that a small change in I correlates with a large change in V. Unlike the membrane resistance, these curves explicitly remove the feed-forward effects that can arise from sodium current activation.

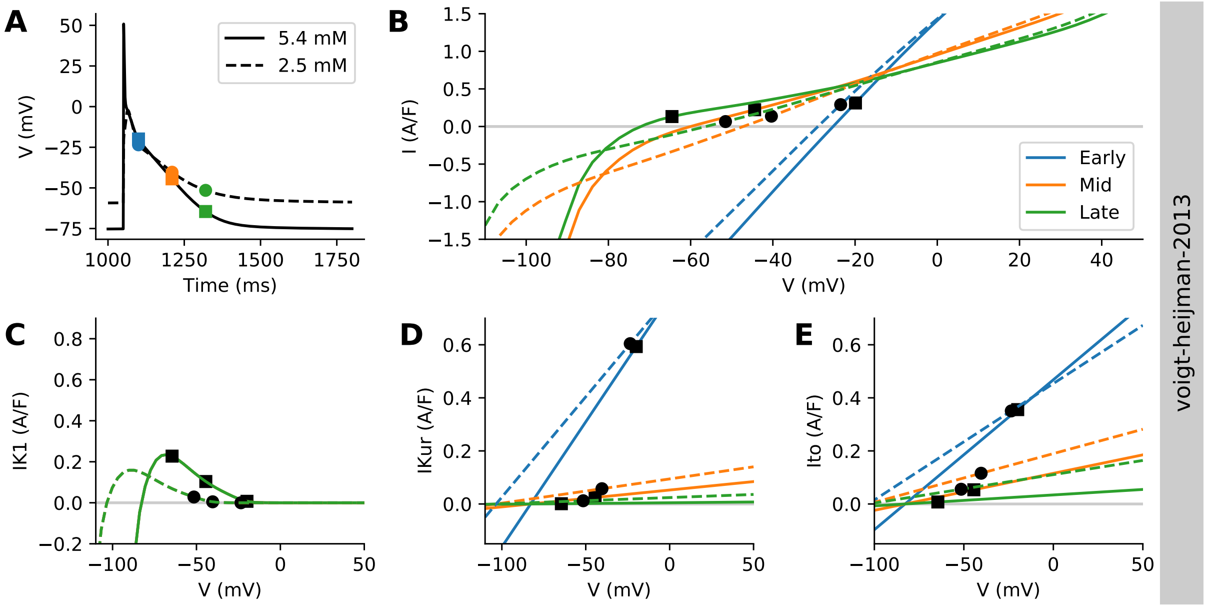

Figure S31: AP and instantaneous IV curves for net current, I_{K1}, I_{Kur}, and I_{to} in the models by Voigt et al. (2013) and . The APs are shown in black, with dashed lines denoting a hypokalemic case. Three points during repolarization are highlighted in blue (early), orange (mid), and green (late stages). IV relationships are plotted at each of these three points, shown by lines in the corresponding colors. The corresponding membrane potentials are shown with black markers and as before, the dashed lines indicate the hypokalemic case. Note the steep slope of net current I early in the AP (blue lines), corresponding to a low membrane resistance, but the significantly reduced slope in the later phases, suggesting that small changes in I are correlated with large changes in V. This shows that small changes in currents active in the late repolarization phase can have large effects on action potential and action potential duration.

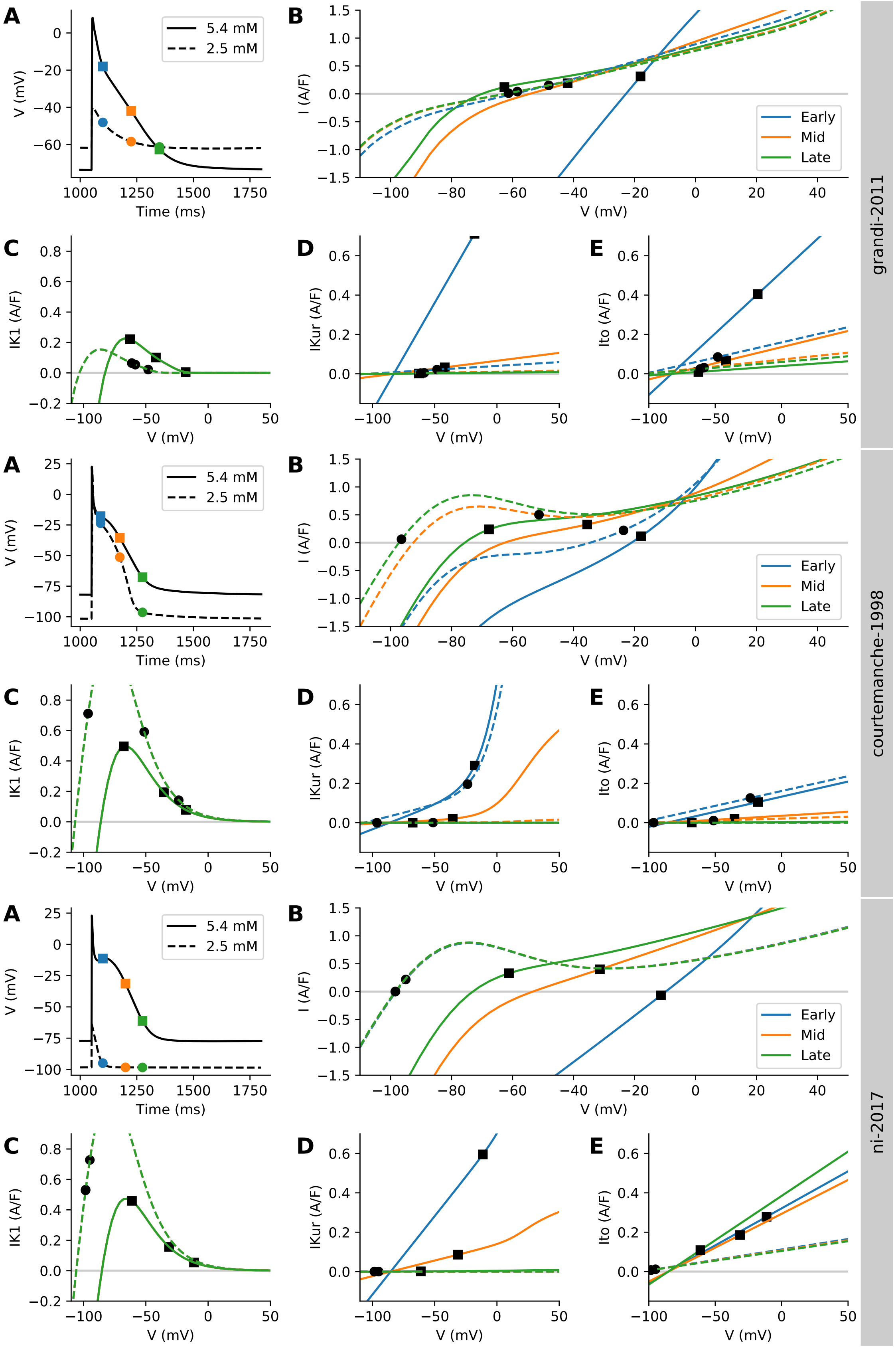

Figure S32: AP and instantaneous IV curves for net current, I_{K1}, I_{Kur}, and I_{to} in the models by Grandi et al. (2011), Courtemanche et al. (1998), and Ni et al. (2017).

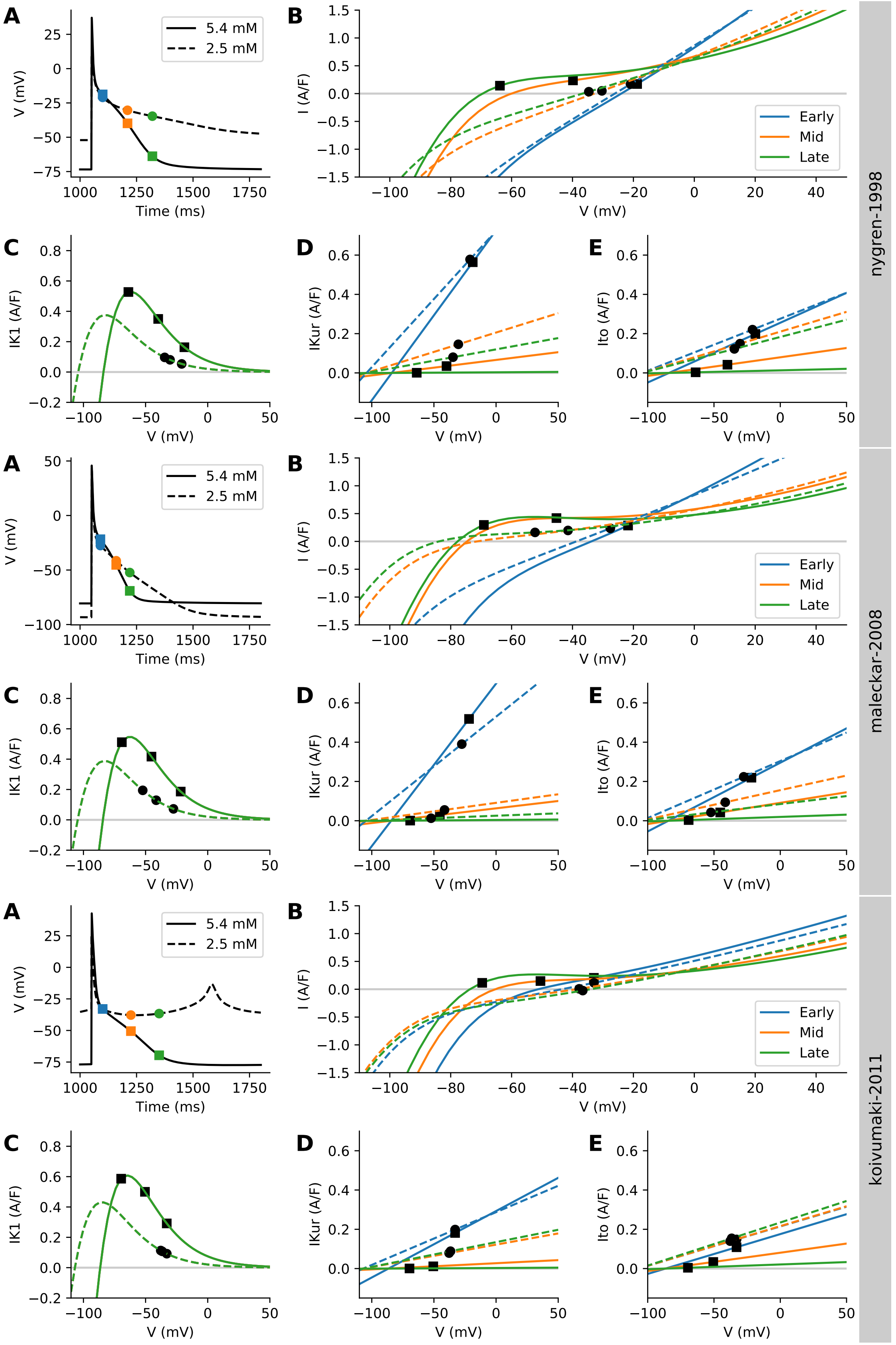

Figure S33: AP and instantaneous IV curves for net current, I_{K1}, I_{Kur}, and I_{to} in the models by Nygren et al. (1998), Maleckar et al. (2008), and Koivumäki et al. (2011).

References

Colman, M.A., Aslanidi, O.V., Kharche, S., Boyett, M.R., Garratt, C., Hancox, J.C., Zhang, H., 2013. Pro-arrhythmogenic effects of atrial fibrillation-induced electrical remodelling: insights from the three-dimensional virtual human atria. The Journal of physiology 591, 4249–4272.

Colman, M.A., Ni, H., Liang, B., Schmitt, N., Zhang, H., 2017. In silico assessment of genetic variation in kcna5 reveals multiple mechanisms of human atrial arrhythmogenesis. PLoS computational biology 13, e1005587.

Courtemanche, M., Ramirez, R.J., Nattel, S., 1998. Ionic mechanisms underlying human atrial action potential properties: insights from a mathematical model. American Journal of Physiology. Heart and Circulatory Physiology 275, H301–H321.

Fink, M., Giles, W.R., Noble, D., 2006. Contributions of inwardly rectifying K+ currents to repolarization assessed using mathematical models of human ventricular myocytes. Philosophical Transactions of the Royal Society of London A: Mathematical, Physical and Engineering Sciences 364, 1207–1222.

Gelband, H., Bush, H.L., Rosen, M.R., Myerburg, R.J., Hoffman, B.F., 1972. Electrophysiologic properties of isolated preparations of human atrial myocardium. Circulation research 30, 293–300.

Grandi, E., Pandit, S.V., Voigt, N., Workman, A.J., Dobrev, D., Jalife, J., Bers, D.M., 2011. Human atrial action potential and Ca2+ model sinus rhythm and chronic atrial fibrillation. Circulation Research 109, 1055–1066.

Grandi, E., Pasqualini, F.S., Bers, D.M., 2010. A novel computational model of the human ventricular action potential and Ca transient. Journal of Molecular and Cellular Cardiology 48, 112–121.

Jack, J.J.B., Noble, D., Tsien, R.W., 1975. Electric current flow in excitable cells. Clarendon Press Oxford.

Jacquemet, V., 2007. Steady-state solutions in mathematical models of atrial cell electrophysiology and their stability. Mathematical biosciences 208, 241–269.

Koivumäki, J.T., Korhonen, T., Tavi, P., 2011. Impact of sarcoplasmic reticulum calcium release on calcium dynamics and action potential morphology in human atrial myocytes: a computational study. PLOS Computational Biology 7, e1001067.

Lindblad, D., Murphey, C., Clark, J., Giles, W., 1996. A model of the action potential and underlying membrane currents in a rabbit atrial cell. American Journal of Physiology 271, H1666–H1696.

Lu, Z., 2004. Mechanism of rectification in inward-rectifier K+ channels. Annual Review of Physiology 66, 103–129.

Luo, C.H., Rudy, Y., 1991. A model of the ventricular cardiac action potential. depolarization, repolarization, and their interaction. Circulation Research 68, 1501–1526.

Luo, C.h., Rudy, Y., 1994. A dynamic model of the cardiac ventricular action potential. I. simulations of ionic currents and concentration changes. Circulation Research 74, 1071–1096.

Maleckar, M.M., Greenstein, J.L., Trayanova, N.A., Giles, W.R., 2008. Mathematical simulations of ligand-gated and cell-type specific effects on the action potential of human atrium. Progress in Biophysics and Molecular Biology 98, 161–170.

Ni, H., Whittaker, D.G., Wang, W., Giles, W.R., Narayan, S.M., Zhang, H., 2017. Synergistic anti-arrhythmic effects in human atria with combined use of sodium blockers and acacetin. Frontiers in physiology 8, 946.

Nygren, A., Fiset, C., Firek, L., Clark, J., Lindblad, D., Clark, R., Giles, W., 1998. Mathematical model of an adult human atrial cell the role of K+ currents in repolarization. Circulation Research 82, 63–81.

Passini, E., Genovesi, S., Severi, S., 2014. Human atrial cell models to analyse haemodialysis-related effects on cardiac electrophysiology: work in progress. Computational and mathematical methods in medicine 2014.

Pouranbarani, E., dos Santos, R.W., Nygren, A., 2019. A robust multi-objective optimization framework to capture both cellular and intercellular properties in cardiac cellular model tuning: Analyzing different regions of membrane resistance profile in parameter fitting. PloS one 14.

Shannon, T.R., Wang, F., Puglisi, J., Weber, C., Bers, D.M., 2004. A mathematical treatment of integrated Ca dynamics within the ventricular myocyte. Biophysical Journal 87, 3351–3371.

Spitzer, K.W., Pollard, A.E., Yang, L., Zaniboni, M., Cordeiro, J.M., Huelsing, D.J., 2006. Cell-to-cell electrical interactions during early and late repolarization. Journal of cardiovascular electrophysiology 17, S8–S14.

Ten Eick, R., Singer, D., 1979. Electrophysiological properties of diseased human atrium. i. low diastolic potential and altered cellular response to potassium. Circulation research 44, 545–557.

Trenor, B., Cardona, K., Saiz, J., Noble, D., Giles, W., 2017. Cardiac action potential repolarization revisited: early repolarization shows all-or-none behaviour. The Journal of Physiology 595, 6599–6612.

Van Wagoner, D.R., Pond, A.L., Lamorgese, M., Rossie, S.S., McCarthy, P.M., Nerbonne, J.M., 1999. Atrial [l]-type [ca2+] currents and human atrial fibrillation. Circulation research 85, 428–436.

Voigt, N., Heijman, J., Trausch, A., Mintert-Jancke, E., Pott, L., Ravens, U., Dobrev, D., 2013. Impaired Na+-dependent regulation of acetylcholine-activated inward-rectifier K+ current modulates action potential rate dependence in patients with chronic atrial fibrillation. Journal of molecular and cellular cardiology 61, 142–152.

Weiss, J.N., Qu, Z., Shivkumar, K., 2017. Electrophysiology of hypokalemia and hyperkalemia. Circulation: Arrhythmia and Electrophysiology 10, e004667.

Wilhelms, M., Hettmann, H., Maleckar, M.M.C., Koivumäki, J.T., Dössel, O., Seemann, G., 2013. Benchmarking electrophysiological models of human atrial myocytes. Frontiers in Physiology 3, 487.

Xie, Y., Sato, D., Garfinkel, A., Qu, Z., Weiss, J.N., 2010. So little source, so much sink: requirements for afterdepolarizations to propagate in tissue. Biophysical journal 99, 1408–1415.

Zaniboni, M., 2011. 3D current–voltage–time surfaces unveil critical repolarization differences underlying similar cardiac action potentials: A model study. Mathematical biosciences 233, 98–110.

Zaniboni, M., Cacciani, F., 2014. Instantaneous current–voltage relationships during the course of the human cardiac ventricular action potential: new computational insights into repolarization dynamics. Europace 16, 774–784.

Zaniboni, M., Pollard, A.E., Yang, L., Spitzer, K.W., 2000. Beat-to-beat repolarization variability in ventricular myocytes and its suppression by electrical coupling. American Journal of Physiology-Heart and Circulatory Physiology 278, H677–H687.
